# 2’-O-methyltransferase-deficient yellow fever virus: Restricted replication in the midgut and secondary tissues of *Aedes aegypti* mosquitoes severely limits dissemination

**DOI:** 10.1371/journal.ppat.1012607

**Published:** 2024-10-02

**Authors:** Anja vom Hemdt, Alexandra L. Thienel, Katrin Ciupka, Janett Wieseler, Hannah M. Proksch, Martin Schlee, Beate M. Kümmerer

**Affiliations:** 1 Institute of Virology, Medical Faculty, University of Bonn, Bonn, Germany; 2 Institute of Clinical Chemistry and Clinical Pharmacology, Medical Faculty, University of Bonn, Bonn, Germany; 3 German Centre for Infection Research, Partner Site Bonn-Cologne, Bonn, Germany; Colorado State University, UNITED STATES OF AMERICA

## Abstract

The RNA genome of orthoflaviviruses encodes a methyltransferase within the non-structural protein NS5, which is involved in 2’-O-methylation of the 5’-terminal nucleotide of the viral genome resulting in a cap1 structure. While a 2’-O-unmethylated cap0 structure is recognized in vertebrates by the RNA sensor RIG-I, the cap1 structure allows orthoflaviviruses to evade the vertebrate innate immune system. Here, we analyzed whether the cap0 structure is also recognized in mosquitoes. Replication analyses of 2’-O-methyltransferase deficient yellow fever virus mutants (YFV NS5-E218A) of the vaccine 17D and the wild-type Asibi strain in mosquito cells revealed a distinct downregulation of the cap0 viruses. Interestingly, the level of inhibition differed for various mosquito cells. The most striking difference was found in *Aedes albopictus*-derived C6/36 cells with YFV-17D cap0 replication being completely blocked. Replication of YFV-Asibi cap0 was also suppressed in mosquito cells but to a lower extent. Analyses using chimeras between YFV-17D and YFV-Asibi suggest that a synergistic effect of several mutations across the viral genome accompanied by a faster initial growth rate of YFV-Asibi cap1 correlates with the lower level of YFV-Asibi cap0 attenuation. Viral growth analyses in Dicer-2 knockout cells demonstrated that Dicer-2 is entirely dispensable for attenuating the YFV cap0 viruses. Translation of a replication-incompetent cap0 reporter YFV-17D genome was reduced in mosquito cells, indicating a cap0 sensing translation regulation mechanism. Further, oral infection of *Aedes aegypti* mosquitoes resulted in lower infection rates for YFV-Asibi cap0. The latter is related to lower viral loads found in the midguts, which largely diminished dissemination to secondary tissues. After intrathoracic infection, YFV-Asibi cap0 replicated slower and to decreased amounts in secondary tissues compared to YFV-Asibi cap1. These results suggest the existence of an ubiquitously expressed innate antiviral protein recognizing 5’-terminal RNA cap-modifications in mosquitoes, both in the midgut as well as in secondary tissues.

## Author summary

Arthropod-borne viruses infect both mosquito and vertebrate hosts. One factor influencing the level of viral replication in the host is the ability of the virus to evade or counteract the host’s immune system. In vertebrates, it was shown that orthoflaviviruses circumvent the innate immune system by capping their viral genome with a cap1 structure characterized by 2’-O-methylation of the 5’-terminal nucleotide. In contrast, viral genomes lacking 2’-O-methylation (cap0) are recognized as foreign and activate the vertebrate innate immune system. Here, we used yellow fever virus (YFV) strain 17D and Asibi mutants lacking 2’-O-methylation, as well as a cap0 reporter YFV genome, to analyze cap0 recognition in mosquito cells and living mosquitoes. The replication of the YFV cap0 mutants was profoundly impaired in different mosquito cells and cap0 YFV genomes showed reduced translation levels. Based on viral infection experiments in *Aedes aegypti* mosquitoes, we further demonstrate that these vector mosquitoes recognize the cap0 structure and that a potential recognizing effector protein is localized in the midgut as well as in secondary tissues. Our findings provide the basis for future studies investigating the recognition of foreign RNAs in mosquitoes and should contribute to establish methods to prevent transmission of arboviruses.

## Introduction

Yellow fever virus (YFV) is an enveloped, mosquito-borne virus belonging to the genus *Orthoflavivirus* within the family *Flaviviridae* [1]. It is primarily transmitted by *Aedes aegypti* mosquitoes and profoundly affects public health despite the existence of an efficient vaccine (YFV-17D) [2]. It is estimated that 200,000 YFV infections emerge each year [3].

Orthoflaviviruses harbor an 11 kb RNA genome with a positive polarity, which has a cap1 structure at its 5’ end (m7GpppAmp) and is not polyadenylated at its 3’ end. The genome encodes for one open-reading frame flanked by a 5’ and a 3’ untranslated region (UTR) [4]. Following the translation of the genomic RNA into a single polyprotein, the latter is co- and posttranslationally cleaved by cellular and viral proteases into the three structural proteins (SP) C, prM, and E, and the seven non-structural (NS) proteins NS1, NS2A, NS2B, NS3, NS4A, NS4B, and NS5.

While cellular RNA is capped in the nucleus, orthoflaviviral replication and RNA capping occur in the cytoplasm. The capping reaction generally consists of three steps: I) removal of the 5’ phosphate by the triphosphatase to generate a 5’ diphosphate; II) guanosine monophosphate (GMP) transfer to the dephosphorylated RNA by the guanylyltransferase (GTase); III) methylation at the guanine N-7 position by the N-7-methyltransferase (N-7-MTase) [5,6]. Orthoflaviviral RNAs are further methylated at the ribose 2’-O position of the first nucleotide by the 2’-O-methyltransferase (2’-O-MTase) to form cap1 (m7GpppNm) [7,8]. Orthoflaviviruses cannot access the cellular capping machinery and therefore encode their own capping enzymes. Both NS3 and NS5 mediate the cap formation of orthoflaviviral RNA. The NS3 protein encodes a triphosphatase [9] and the NS5 protein an RNA GTase [10] as well as a methyltransferase (MTase) able to mediate guanine N-7 and ribose 2’-O-methylation [11].

The 2’-O-MTase is highly conserved between orthoflaviviruses, and the motif NS5-K61-D146-K182-E218 forms the active site for the 2’-O-methyltransfer reaction [12]. For several orthoflaviviruses, it was shown that all four amino acids are essential for the 2’-O-MTase activity, but only D146 is essential for the N-7-MTase activity [8, 13]. Substitution experiments revealed reduced replication of 2’-O-MTase deficient viruses (E218A) in an interferon-dependent manner due to the recognition of the uncapped viruses by the receptor retinoic acid-inducible gene-I (RIG-I) and interferon induced protein with tetratricopeptide (IFIT1) [14–18]. While these data show the importance of a cap1 structure to evade the vertebrate immune system, little is known about the importance of this structure in evading the mosquito immune system.

Mosquito-borne orthoflaviviruses need the ability to replicate in vertebrate and mosquito hosts, meaning they need to overcome both immune systems. Since the cap1 structure of orthoflaviviruses seems crucial to evade the vertebrate immune system, it was tempting to speculate that this structure is also important to escape the mosquito immune system. In contrast to the vertebrate immune system, mosquitoes rely on innate immune mechanisms and lack adaptive immune mechanisms that are based on antigen recognition [19]. One of the main antiviral immune pathways in mosquitoes is the RNA interference pathway (RNAi), which involves the distinct degradation of target RNAs [20]. Depending on the RNA origin, three classes of RNAi machineries are differentiated: small interfering RNA (siRNA) pathway, micro RNA (miRNA) pathway, and PIWI-interacting (piRNA) pathway [19]. Viral dsRNAs, formed as replication intermediates, are mainly sensed through the siRNA pathway [21]. The viral dsRNA is cleaved into 21 nucleotide-long siRNAs by the RNase III-like enzyme Dicer-2 (Dcr-2) and its co-factor R2D2 [22]. The resulting siRNAs are loaded onto Argonaute-2 (Ago2) within the RNA-induced silencing complex (RISC) [23, 24]. The endonuclease Ago2 cleaves the passenger strand, which is removed from the complex. The remaining guide strand binds complementary RNAs to target them for degradation (21). Interestingly, Dcr-2 and RIG-I interact with dsRNA through their DExD/H-box helicase domain [25, 26].

In this study, we aimed to investigate the importance of the 2’-O-methylation at the first nucleotide of the viral RNA for replication of YFV in mosquitoes, using both *in vitro* and *in vivo* analyses. We show that 2’-O-methylation deficient YFV-17D and YFV-Asibi (hereafter referred to as cap0 viruses) are distinctly downregulated in mosquito cells compared to the corresponding wild-type viruses with cap 2’-O-methylation (hereafter referred to as cap1 viruses) but to different extents. While no growth was observed for YFV-17D cap0 in C6/36 mosquito cells, replication of YFV-Asibi cap0 was detectable. Chimeras between YFV-17D and YFV-Asibi revealed that a synergy of several proteins is responsible for the different replication levels of the two YFV strains lacking the 2’-O-methylation in mosquito cells. Further, it seemed that the ability of YFV-Asibi cap0 to grow in C6/36 mosquito cells correlates with an initial faster growth of the parental YFV-Asibi cap1 virus compared to YFV-17D cap1. The reduction of viruses lacking the 2’-O-methylation was entirely independent of Dcr-2. This suggests that the cap0 structure is recognized by an effector protein in mosquito cells, which is not linked to the well-characterized siRNA pathway and also not to a putative RNAi independent antiviral pathway induced by Dcr-2. Further, reduced translation of a replication-incompetent cap0 reporter YFV-17D genome in mosquito cells hints towards a cap0 sensing translation regulation mechanism, similar to the inhibition of translation of cap0 mRNAs by IFIT1 described in vertebrates [27]. *In vivo* experiments in *Aedes aegypti* showed a lower infection rate for YFV-Asibi cap0, which was associated with a lower viral load in the midgut of the mosquitoes, also strongly reducing dissemination. After intrathoracic injection, YFV-Asibi cap0 replicated slower and to decreased amounts in secondary tissues compared to YFV-Asibi cap1 suggesting that inhibition of YFV-Asibi cap0 occurs both at the midgut barrier level as well as in secondary tissues. Taken together, this study provides an exciting basis to further dissect the cap0 recognition in mosquitoes.

## Results

### Replication of 2’-O-MTase deficient YFV-17D cap0 and YFV-Asibi cap0 is impaired in mosquito cells to different extents

**Fig 1:**
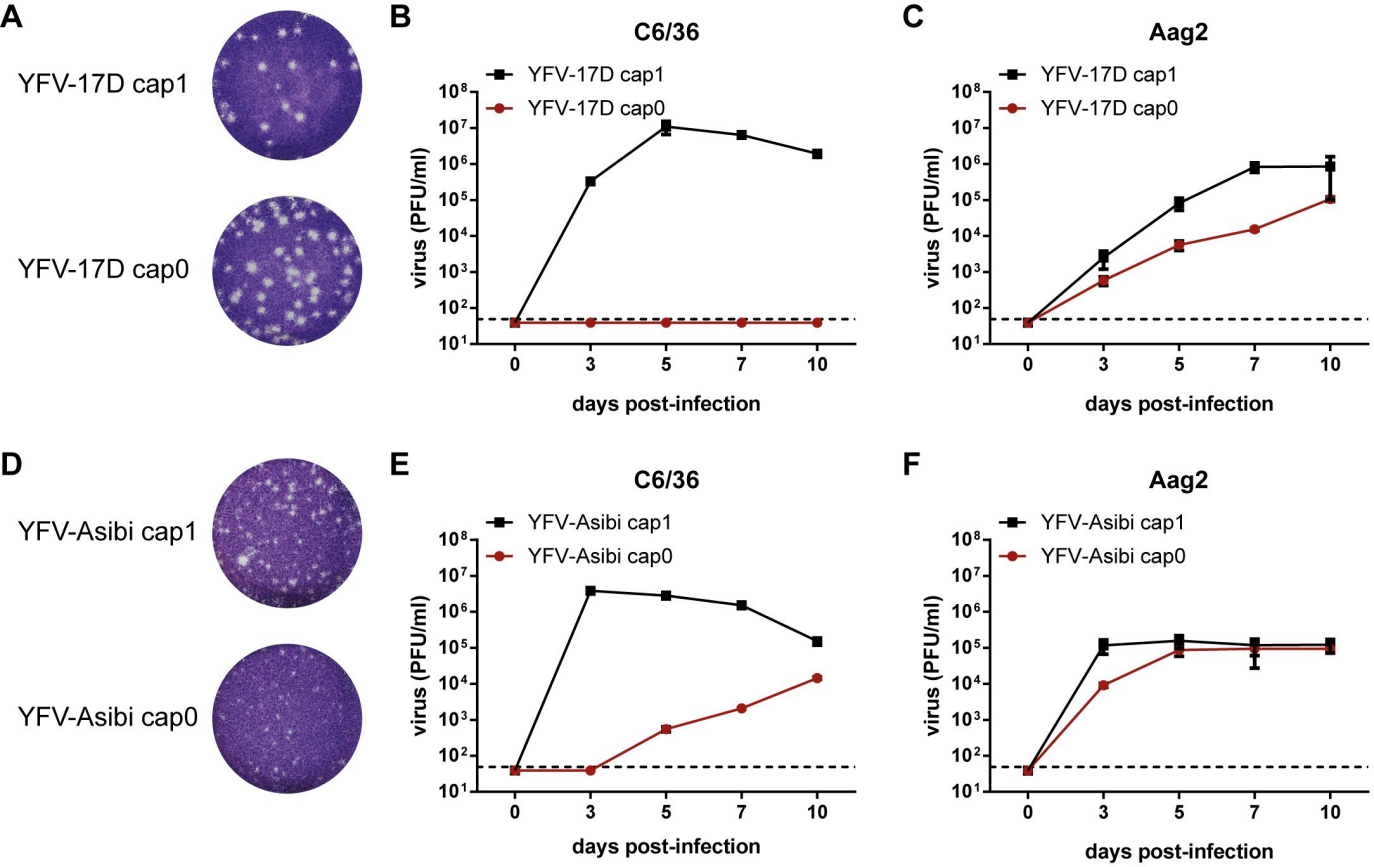
Inhibition of YFV-17D cap0 and -Asibi cap0 in different mosquito cells. (A) Plaque phenotypes of YFV-17D cap1 and cap0 and (D) YFV-Asibi cap1 and cap0 after performing an infectious center assay in BHK cells. At 3 days post-infection, cells were fixed and stained by crystal violet. (B, C) Growth kinetics of YFV-17D cap1 and cap0 in C6/36 and Aag2 cells. (E, F) Growth kinetics of YFV-Asibi cap1 and cap0 in C6/36 and Aag2 cells. Cells were infected at a multiplicity of infection (MOI) of 0.01 and viral titers were measured at 0, 3, 5, 7, and 10 days post-infection by titration on BHK cells. Data represent Mean ± SD of triplicates. Dashed lines: detection limit.

Our previous studies demonstrated that YFV-17D cap0 is attenuated in immunocompetent vertebrate cells [17]. We were further interested to analyze whether replication of a YFV genome encompassing a cap0 structure also results in reduced growth in mosquito cells. Taking advantage of the fact that orthoflaviviruses encode their own 2’-O-MTase to generate a cap1 structure at the 5’ end of their viral genome [11], we first used the YFV vaccine strain 17D in which the 2’-O-MTase activity was abrogated by mutation of NS5-E218 to A [17]. Plaques formed by YFV-17D cap1 and cap0 in BHK cells, which are deficient in type I interferon activation [28], were similar in size (Fig 1A). The replication of the viruses was further characterized by infecting different mosquito cell lines at a multiplicity of infection (MOI) of 0.01. YFV-17D cap1 readily replicated over time in *Aedes albopictus*-derived C6/36 cells and reached its peak titer five days post-infection before entering a plateau (Fig 1B). In contrast, YFV-17D cap0 was highly attenuated and did not exceed the detection limit at any tested time point (Fig 1B). Both viruses replicated in *Aedes aegypti-*derived Aag2 cells but to different endpoint titers. YFV-17D cap0 replicated to about 1 log lower titers than YFV-17D cap1 at ten days post-infection (Fig 1C). Similarly, YFV-17D cap1 also replicated to higher titers compared to YFV cap0 in the *Aedes albopictus*-derived cell line U4.4 and in the *Aedes aegypti*-derived cell line CCL-125 as measured at five days post-infection (S1 Fig). In U4.4 cells, YFV-17D cap0 was reduced by about 0.7 log compared to YFV-17D cap1 (S1A Fig). In general, CCL-125 cells supported the growth of YFV-17D to a lower extent than the other insect cells, reaching only peak titers in the range of 1 x 10^3^ PFU/ml (S1B Fig). While YFV-17D cap1 reached a titer of 667 PFU/ml at five days post-infection in CCL-125 cells, the cap0 variant did not exceed the detection limit (S1B Fig).

We further analyzed whether the complete inhibition of the cap0 variant observed in the C6/36 cells was specific to the vaccine strain or also applies to wild-type YFV strains. In addition, we aimed to analyze the replication efficiency of YFV in living mosquitoes in later experiments. As it is known that replication of the vaccine strain is inhibited in mosquitoes at the midgut barrier level [29], we established an infectious YFV-Asibi clone based on an available Asibi precursor clone (kindly provided by Charles M. Rice). Both YFV-Asibi cap1 and cap0 could be rescued successfully in BHK cells, where they replicated to comparable titers (S2A Fig). In contrast to the rather large plaque sizes of YFV-17D cap1 and cap0 (Fig 1A), the plaques formed by YFV-Asibi cap1 and cap0 displayed a smaller diameter (Fig 1D). In the immunocompetent mammalian A549 cell line, replication of the YFV-Asibi cap0 mutant was significantly reduced compared to its cap1 counterpart but was restored upon knockout (k.o.) of IFIT1 indirectly confirming the presence of the cap0 structure (S2B and S2C Fig). The infection experiments in mosquito cells revealed that in C6/36 cells an efficient growth was observed for YFV-Asibi cap1, peaking already three days post-infection (3.8 x 10^6^ PFU/ml) (Fig 1E). At this time point, YFV-Asibi cap0 did not exceed the detection limit yet, but thereafter its replication continuously increased. Still, compared to the cap1 virus, the cap0 virus was highly attenuated (Fig 1E). In Aag2 cells, YFV-Asibi cap0 produced an over 1 log lower viral titer than the cap1 virus on day three post-infection (Fig 1F). This difference neglected itself over time with equivalent endpoint titers. Taken together, these data suggest that the YFV cap0 structure is also recognized in mosquito cells resulting in attenuation of the respective YF viruses.

### Ability of YFV-Asibi cap0 to grow in C6/36 cells likely correlates with an initial faster growth rate of YFV-Asibi cap1 attributed to a synergistic effect of several mutations across the genome

YFV-Asibi cap0 was, in contrast to YFV-17D cap0, able to replicate to low titers in C6/36 cells. Therefore, we were interested to analyze whether a certain protein of YFV-Asibi is able to counteract the recognition of the cap0 structure allowing YFV-Asibi cap0 to grow.

**Fig 2:**
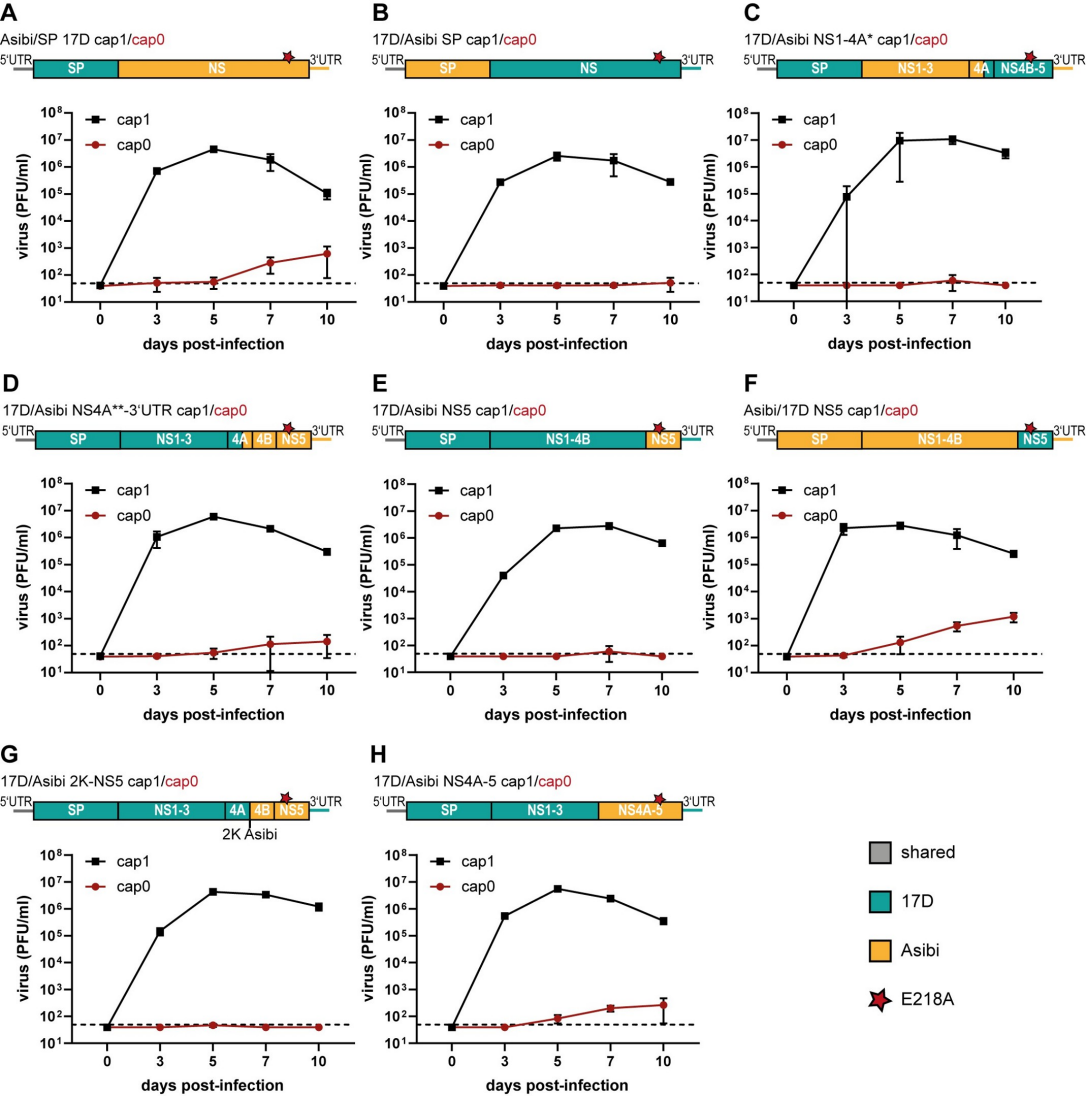
YFV-Asibi NS4A-5 promotes replication of the cap0 virus. The colors represent the sequence source (green: YFV-17D; yellow: YFV-Asibi), and shared sequences are those that do not differ between Asibi and 17D (grey). The red star represents the E218A exchange in the NS5 protein. A) Growth kinetics of Asibi/17D SP cap1 and cap0 in C6/36 cells. B) Growth kinetics of 17D/Asibi SP cap1 and cap0 in C6/36 cells. C) Growth kinetics of 17D/Asibi NS1-4A* cap1 and cap0 in C6/36 cells. D) Growth kinetics of 17/Asibi NS4A**-3’UTR cap1 and cap0 in C6/36 cells. E) Growth kinetics of 17D/Asibi NS5 cap1 and cap0 in C6/36 cells. F) Growth kinetics of Asibi/17D NS5 cap1 and cap0 in C6/36 cells. G) Growth kinetics of 17D/Asibi 2K-NS5 cap1 and cap0 in C6/36 cells. H) Growth kinetics of 17D/Asibi NS4A-NS5 cap1 and cap0 in C6/36 cells. The cells were infected at an MOI of 0.01. Viral titers were measured at 0, 3, 5, 7, and 10 days post-infection by titration on BHK cells. Data represent Mean ± SD of at least triplicates. Dashed lines: detection limit.

To this end, we established chimeras between YFV-17D and YFV-Asibi. Exchange of the structural protein (SP) region C-prM-E in YFV-Asibi against the corresponding proteins of YFV-17D resulted in an efficiently growing cap1 chimera (Asibi/SP 17D, Fig 2A). The corresponding cap0 chimera was still able to replicate in C6/36 cells, although to a slightly lower level compared to the parental YFV-Asibi cap0 virus. For the reciprocal chimera, namely 17D/Asibi SP, no growth of the cap0 virus was observed while the cap1 variant still replicated efficiently (Fig 2B). These data suggest that the structural proteins of YFV-Asibi ‐ at least on its own ‐ are not the critical component enabling the YFV-Asibi cap0 virus to grow. This hypothesis was further supported by the finding that exchanging the non-structural (NS) proteins in YFV-17D against the NS proteins of YFV-Asibi resulted in a cap0 virus (17D/Asibi NS1-NS5 cap0) still able to replicate in C6/36 cells (S3A Fig). Hence, we further focused on the NS protein region to map a potential protein involved in the counteraction of cap0 recognition.

In first experiments we took advantage of a singular NgoMIV restriction site in the YFV genome localized within the 3’-terminal third of the NS4A gene. We established YFV-17D chimeras in which either the genome region encompassing NS1 up to the NgoMIV site in NS4A was exchanged against the respective segment of YFV-Asibi (17D/Asibi NS1-4A*) or in which the 3’ terminal part downstream of the NgoMIV site was exchanged against the YFV-Asibi sequence (17D/Asibi NS4A**-3’UTR) (Fig 2C and 2D). While both cap1 variants were able to replicate, only the 17D/Asibi NS4A**-3’UTR cap0 chimera was able to grow above the detection limit at day 7 and day 10 (Fig 2D). In an attempt to narrow down crucial NS proteins, the NS5 protein of YFV-Asibi was introduced into the YFV-17D backbone. This 17D/Asibi NS5 chimera did not allow replication of the cap0 variant, whereas the cap0 version of YFV-Asibi with NS5 of YFV-17D (Asibi/17D NS5) was still able to replicate (Fig 2E and 2F). This suggests that the NS5 protein of Asibi on its own is not responsible for the growth of the Asibi cap0 variant. Still, Asibi NS5 seems to be to some extent important to counteract attenuation of the cap0 variant, since Asibi NS1-4B introduced in the YFV-17D backbone was not able to promote replication of the cap0 virus (S3B Fig). Furthermore, exchanging only the C-terminal part of NS4A or 2K-NS4B also did not allow the corresponding cap0 variants to grow (S3C and S3D Fig). Even a combined exchange of the 2K-NS5 region (17D/Asibi 2K-NS5) did not enable the cap0 variant to replicate above the detection limit, whereas introduction of the Asibi NS4A-NS5 in YFV-17D (17D/Asibi NS4A-5) resulted in some growth (Fig 2G and 2H). However, none of the cap0 chimeras was able to reach the replication efficiency of the YFV-Asibi cap0 virus (Fig 1E).

Altogether it seems that the ability to counteract the recognition of the cap0 structure cannot be linked to an individual protein in YFV-Asibi. Instead, a synergistic effect of several mutations across the genome is necessary with important mutations being localized in the NS4A-NS5 region. Comparing the viral growth kinetics of the different cap1 chimeras to the Asibi and 17D cap0 variants, it was striking that except for the 17D/Asibi NS4B variant, all cap0 viruses whose cap1 counterparts showed fast initial growth rates (high titers at day 3 p.i.) were able to slightly replicate (S4A Fig). This is in accordance with YFV-Asibi cap1 replicating faster than YFV-17D at early time points (S4B Fig). Hence, a fast initial growth rate of the cap1 viruses seems to favor the growth of the corresponding cap0 variants.

### YFV 2’-O-MTase mutants are impaired in mosquito cells in a Dcr-2-independent manner

The endonuclease Dcr-2 interacts, similar to RIG-I, with RNAs through its DExD/H domain [26]. In C6/36 cells, Dcr-2 is C-terminally truncated [30] resulting in the loss of the PAZ domain and the tandem RNase III domains, while the DExD/H domain is still retained. Due to this truncation, C6/36 cells have a non-functional siRNA pathway [31]. This suggests that neither the deleted domains nor the siRNA pathway are involved in attenuation of the cap0 viruses in mosquito cells, since C6/36 cells are able to mediate attenuation. To further investigate the importance of Dcr-2 in recognizing the 2’-O-methylation status of viral RNA, YFV-17D/-Asibi cap1 and cap0 growth curve analyses were performed on previously described Aag2 Dcr-2 k.o. cells and parental control cells [32]. For the Dcr-2 k.o. cells, CRISPR-Cas9 based k.o. was performed using a guide RNA targeting exon 1 of the Dcr-2 gene resulting in the loss of all Dcr-2 domains [32]. To validate again the nature of the knock-out cell line we co-transfected dsRNA against the Renilla luciferase gene (dsRluc) with a Rluc expressing plasmid. Transfection of dsRNA against eGFP served as control. While co-transfection of dsRluc and the Rluc plasmid in the Aag2 AF5 cell line resulted in strong down-regulation of the Rluc activity (over 18,000 fold), high Rluc levels were restored in the Aag2 AF319 cell line (S5 Fig).

**Fig 3:**
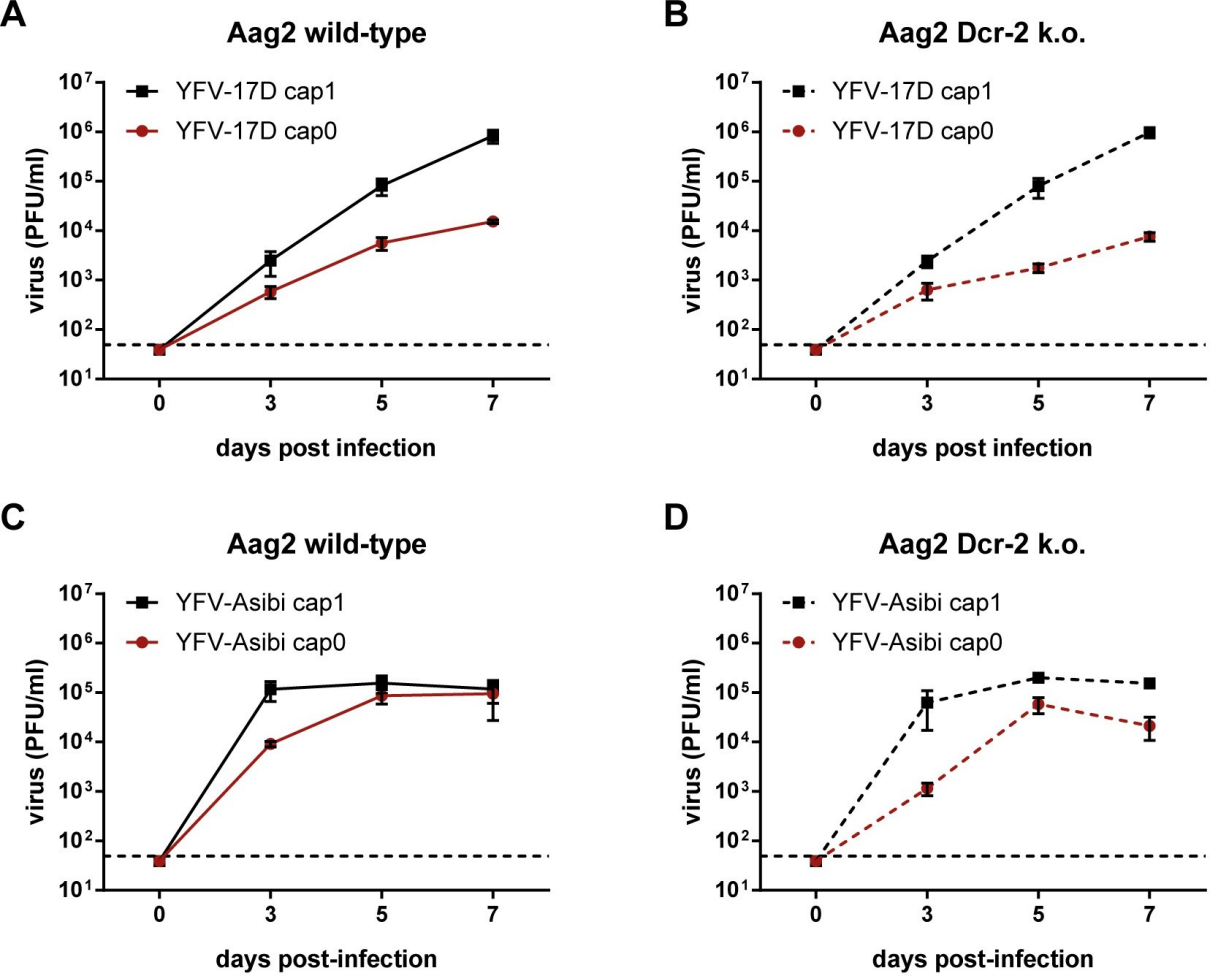
Attenuation of YFV 2’-O-MTase deficient mutants is independent of Dcr-2. (A, B) Parental Aag2 cells or Aag2 Dcr-2 knockout cells were infected with YFV-17D cap1 and cap0 or (C, D) with YFV-Asibi cap1 and cap0 at an MOI of 0.01. Viral titers were measured at 0, 3, 5, and 7 days post-infection by titration on BHK cells. Data represent Mean ± SD of triplicates. Dashed lines: detection limit.

YFV-17D cap1 and cap0 displayed efficient replication in the parental and the Dcr-2 k.o. cells. However, the overall replication of the cap0 virus was distinctly reduced in both cell lines (Fig 3A and 3B). Similarly, replication of YFV-Asibi cap1 showed a higher initial growth rate in the parental cell line than YFV-Asibi cap0. This difference decreased over time as both viruses reached a plateau (Fig 3C). Likewise, a difference in the replication efficiency was present between YFV-Asibi cap1 and cap0 at early time points in the Dcr-2 k.o. cells (Fig 3D).

In summary, the absence of Dcr-2 did not enable the cap0 viruses to grow to viral titers comparable to their cap1 counterparts. Thus, the previously observed replication difference between YFV cap1 and cap0 in mosquito cells is not mediated by the receptor Dcr-2.

### Translation of cap0 YFV RNA is reduced in mosquito cells

**Fig 4:**
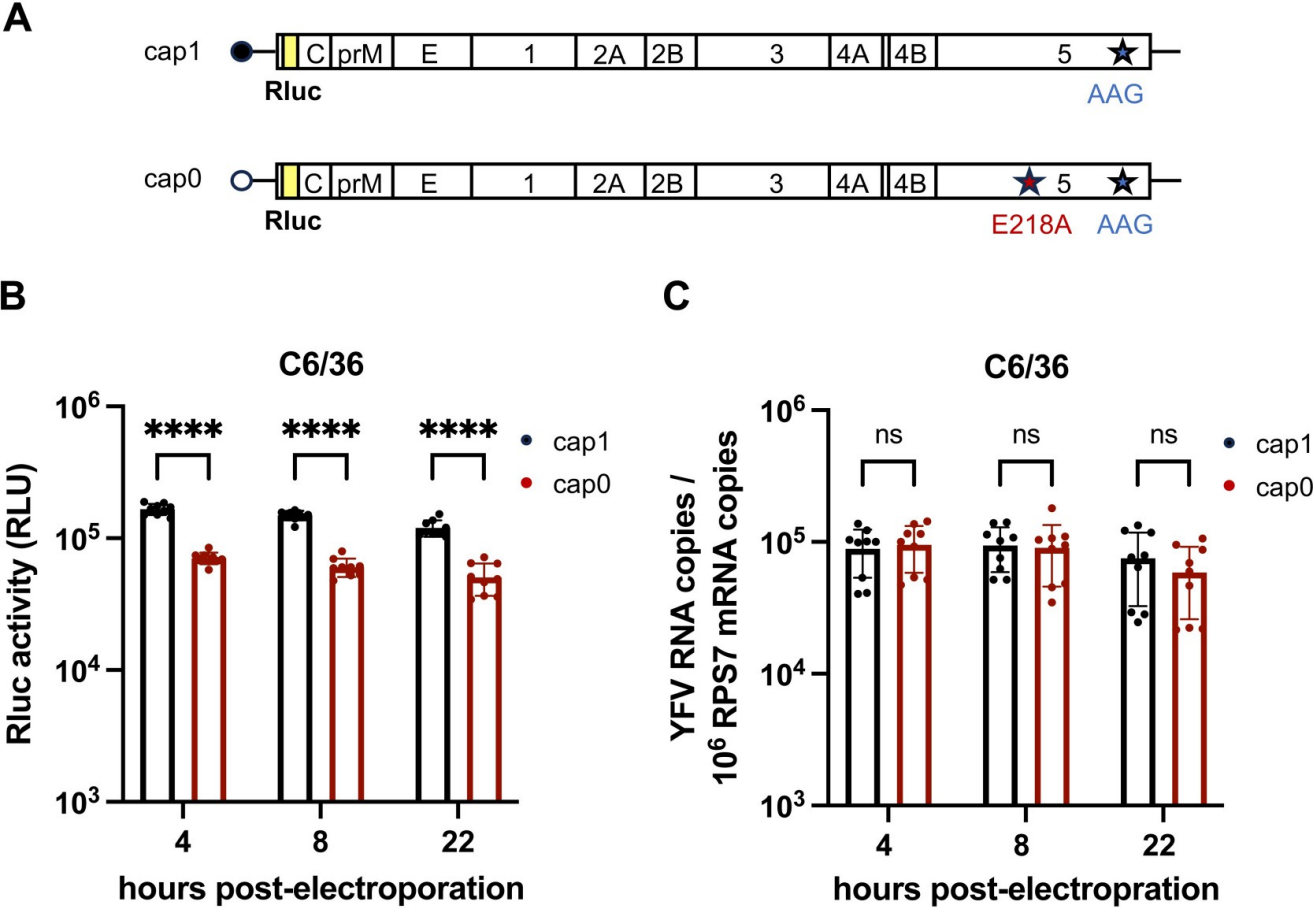
Translation efficiency of a replication-incompetent cap0 reporter YFV genome is reduced in mosquito cells. (**A**) Schematic presentation of the replication-incompetent reporter YFV-17D full-length genomes. The RNA-dependent RNA polymerase activity of YFV was knocked out via mutation (GDD > AAG). *In vitro* transcribed YFV genomes were either modified at their 5’ end by a cap1 structure (WT/AAG genome) or a cap0 structure (E218A/AAG mutant encoding genome). Rluc: Renilla luciferase (yellow box). (B, C) C6/36 cells were electroporated with cap1 or cap0 reporter YFV-17D genome transcripts expressing Rluc. At 4 h, 8 h, and 22 h post-electroporation, cells were either lysed to measure Rluc activity (B) or lysed for RNA isolation to determine the YFV genome copies (C). YFV-17D genome copies were normalized to the housekeeping gene RPS7, with the numbers of YFV-17D genome copies depicted relative to 10^6^ RPS7 gene copies. The means ± SD of n = 3 independent experiments each analyzed with three biological replicates are shown. Individual values are visualized as dots. The Mann-Whitney test was used to calculate statistical significance (**** p ≤ 0.0001, ns = not significant).

For vertebrates it was shown that cap0 mRNAs are sequestered by the IFN-induced protein IFIT1, which impairs binding of proteins required to initiate translation [27]. This results in specific inhibition of cap0 mRNA translation [27]. To analyze whether a similar mechanism occurs in mosquitoes, C6/36 cells were electroporated with replication-incompetent *in vitro* transcribed YFV-17D full-length genomes encoding Rluc (Fig 4A). The respective RNAs were either modified with a 5’ cap1 or 5’ cap0 structure (Fig 4A). Rluc activity was determined at 4, 8, and 22 h after transfection. At all three time points a significantly lower luciferase activity was measured for the cap0 compared to the cap1 YFV RNA (Fig 4B). In contrast, parallel validation of the YFV genome copies normalized to the housekeeping gene RPS7 revealed no significant difference between the cap1 and cap0 RNA levels for each time point (Fig 4C). This indicates that the reduced Rluc levels observed after transfection of the cap0 RNA indeed result from reduced translation levels and are not due to faster degradation of the cap0 RNA.

Similar to the reduced translation levels observed for the cap0 YFV RNA in C6/36 cells, cap0 RNA translation levels were also significantly reduced in Aag2 cells, albeit to a lower extent (S6 Fig). The latter finding is consistent with the viral growth curve analyses, where attenuation of the cap0 viruses was also less pronounced in Aag2 cells compared to C6/36 cells. These data suggest that a potential cap0 binding protein interferes with translation and is expressed at different levels in different mosquito cells.

### 2’-O-methylation is essential for YFV to efficiently replicate and disseminate in *Aedes aegypti*

**Fig 5:**
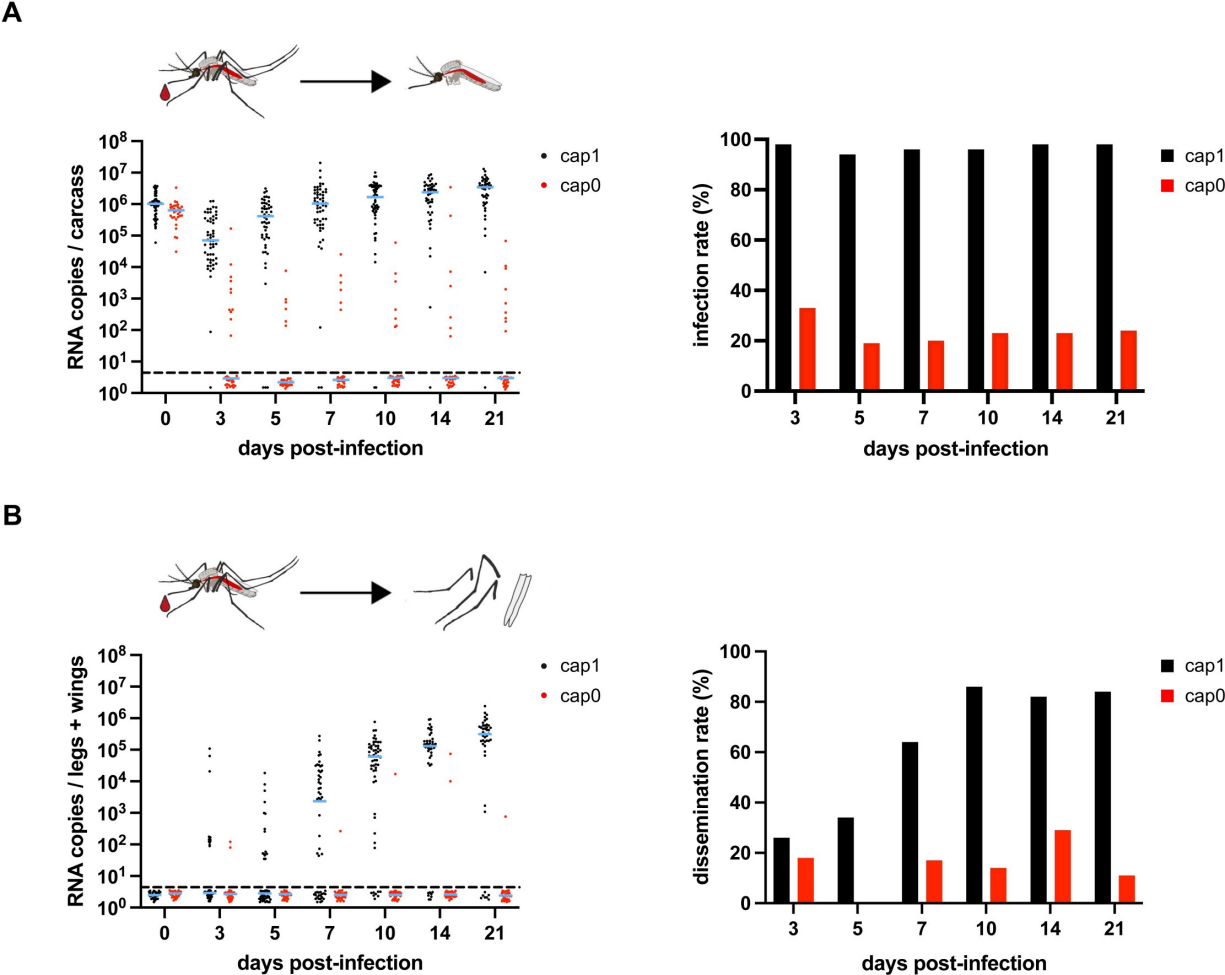
Oral infection of mosquitoes with YFV-Asibi cap0 results in reduced infection and dissemination rates. A, B) Female mosquitoes were orally fed with an infectious blood meal containing 1 x 10^7^ PFU/ml virus. Engorged mosquitoes were kept at 28°C and 80% humidity and were dissected at the indicated time points. A) Viral genome copies (left) and infection rates (right) in the mosquitoes’ carcasses (mosquitoes w/o legs and wings). The viral genome copies were measured by RT-qPCR, and the infection rates were calculated as the number of mosquitoes positive in the carcasses in relation to the total number of examined mosquitoes. B) Viral genome copies in legs plus wings (left) and dissemination rates (right) into secondary tissues. Viral genome copies were determined by RT-qPCR of legs plus wings and dissemination rates were calculated as the number of mosquitoes containing viral RNA in legs plus wings in relation to the number of infected carcasses. Each point represents a single female mosquito. The blue lines indicate the median titers. Dashed line: detection limit.

The obtained *in vitro* data indicate a potential discriminatory mechanism between 2’-O-methylated and unmethylated viral RNA in mosquito cells in a Dcr-2-independent manner affecting translation of viral RNA. To further study whether cap0 recognition also occurs *in vivo*, we compared the replication ability of YFV-Asibi cap1 and cap0 in their natural transmission vector *Aedes aegypti* [33]. Female mosquitoes were blood-fed with 1 x 10^7^ PFU/ml cap1 or cap0 virus. Following infection, the mosquitoes were dissected within three hours post-infection (d0) or at days 3, 5, 7, 10, 14, and 21 post-infection to examine the viral infection and dissemination rates via RT-qPCR. The median viral titers of the blood meal ingested at day zero reached 1 x 10^6^ and 6.3 x 10^5^ RNA copies/carcass (entire mosquito without legs and wings) for YFV-Asibi cap1 and cap0, respectively (Fig 5A). Following an eclipse phase between day zero and three, the viral replication of YFV-Asibi cap1 increased until day seven post-infection in the carcasses until entering a plateau. In line with this, the infection rates remained stable throughout the experiment, ranging between 94% to 98% (Fig 5A and S1 Table). In contrast, the median viral titers of cap0 decreased over time in the carcasses. The infection rates of YFV-Asibi cap0 ranged between 19% to 33% (Fig 5A and S1 Table). The dissemination of YFV-Asibi cap1 into secondary tissues continuously increased and reached dissemination rates of more than 80% from day ten post-infection onwards (Fig 5B and S1 Table). YFV-Asibi cap0 was not detected in secondary tissues except for single mosquitoes (Fig 5B and S1 Table). Analyzing a subset of samples also via plaque assay confirmed the presence of infectious particles in the carcasses for YFV-Asibi cap1 at all time points tested and at later time points of infection also in the legs plus wings (day 7,10, 14, and 21), while no infectious particles were observed for YFV-Asibi cap0 in both sample types over the entire period of the experiment (S7A and S7B Fig).

**Fig 6:**
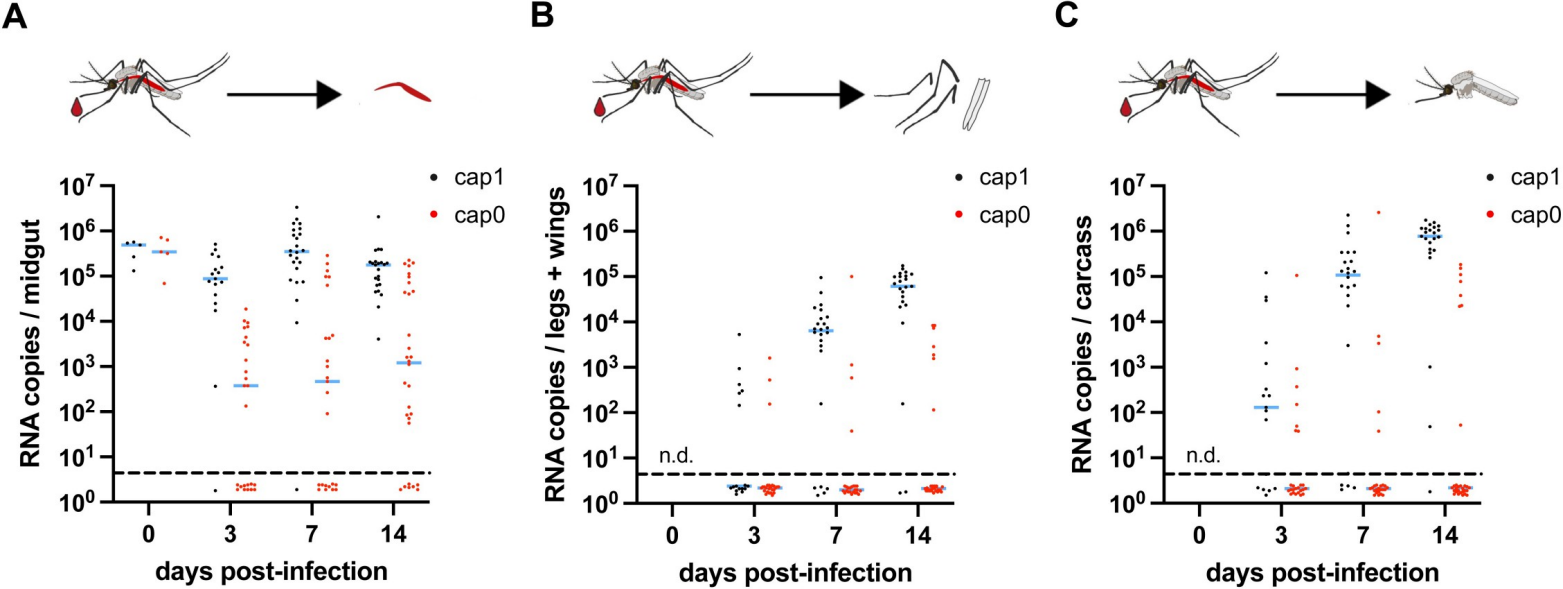
Oral infection with YFV-Asibi cap0 leads to lower viral loads in the midguts largely diminishing dissemination to secondary tissues. A, B, C) Female mosquitoes were orally fed with an infectious blood meal containing 1 x 10^7^ PFU/ml virus. Engorged mosquitoes were kept at 28°C and 80% humidity and were dissected at the indicated time points. Viral genome copies were determined by RT-qPCR in the isolated midguts (A), the legs plus wings (B), and the remaining carcasses (carcasses w/o midgut and w/o legs plus wings) (C). Each point represents a single female mosquito. The blue lines indicate the median titers. Dashed line: detection limit, n.d.: not determined.

The data presented suggest that the midgut barrier might be involved in the reduced infection and dissemination rates observed for YFV-Asibi cap0. To determine the level of replication in the mosquito midgut in more detail, we again orally infected mosquitoes with either YFV-Asibi cap1 or YFV-Asibi cap0 and determined the viral load in the isolated midguts at 0, 3, 7, and 14 days post-infection. At day zero post-infection, similar median viral titers were obtained for the ingested blood meal (Fig 6A). At later time points, the median viral genome copies determined via RT-qPCR were lower for the YFV-Asibi cap0 infected mosquitoes compared to the YFV-Asibi cap1 infected mosquitoes (Fig 6A) supporting restriction of the cap0 virus at the midgut barrier level. Again, reduced dissemination was observed for the YFV-Asibi cap0 virus as was seen when analyzing both legs plus wings as well as the remaining carcasses (mosquito w/o midgut and w/o legs and wings) (Fig 6B and 6C).

**Fig 7:**
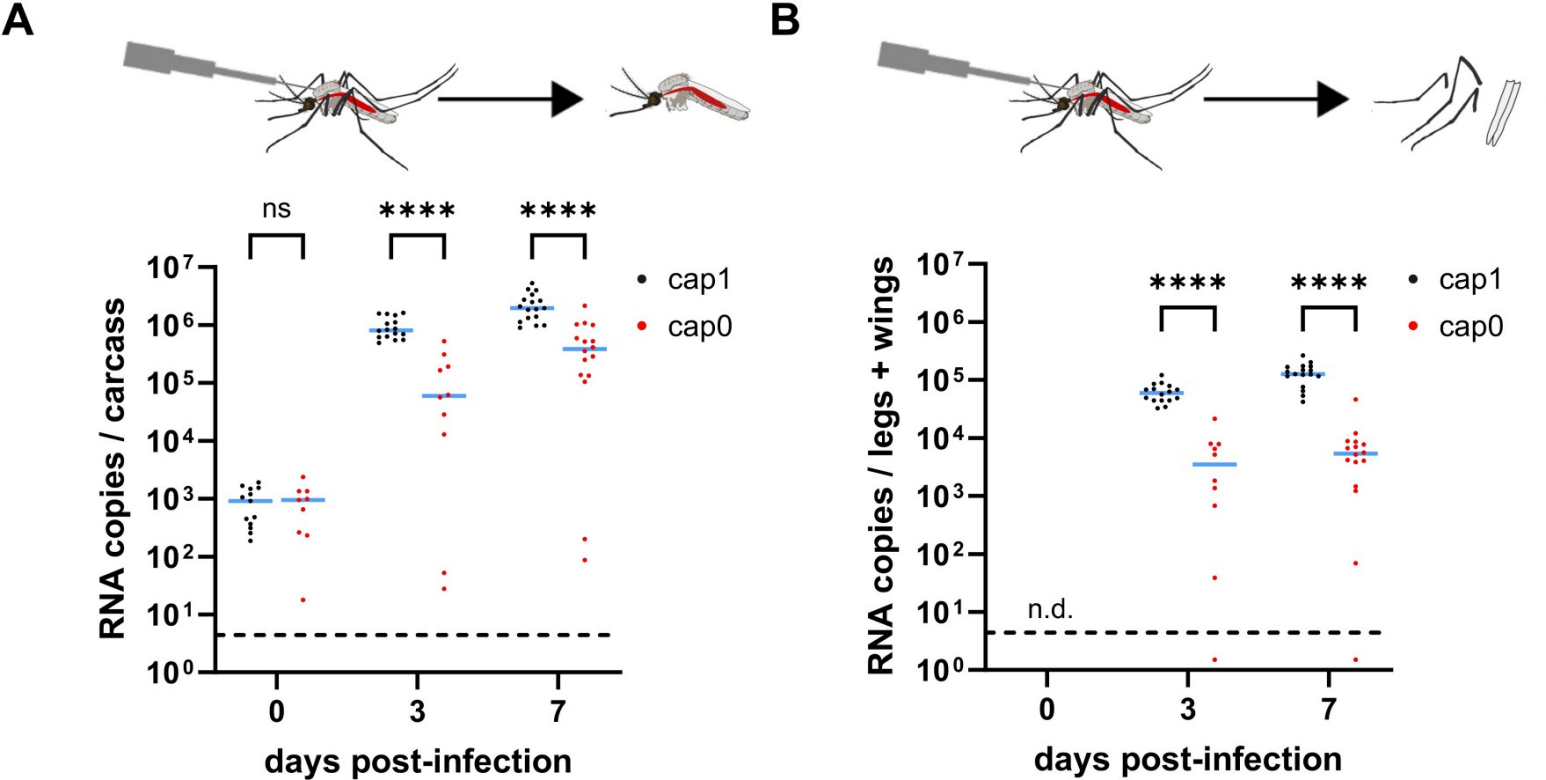
YFV-Asibi cap0 grows slower and to reduced viral titers in secondary tissues after intrathoracic injection. A, B) *Aedes aegypti* mosquitoes were intrathoracically infected with 300 PFU YFV-Asibi cap1 or cap0. At 0, 3, and 7 days post-injection, viral genome copies were determined in the carcasses (mosquitoes w/o legs and wings) (A) and in the legs plus wings (B). The blue lines indicate the median titers and stars the significance calculated by the Mann-Whitney test (**** p ≤ 0.0001; ns = not significant). Dashed line: detection limit, n.d.: not determined.

To investigate whether the cap0 structure is also recognized in tissues beyond the midgut, we intrathoracically infected *Aedes aegypti* mosquitoes with YFV-Asibi cap1 and cap0 to circumvent the midgut barrier. Injected mosquitoes were dissected at day 0, 3 and 7 into legs plus wings and the remaining carcass (mosquito w/o legs plus wings). Median viral titers for cap1 and cap0 viruses increased between day zero and seven post-injection in either type of tissue samples (Fig 7A and 7B). However, YFV-Asibi cap0 replicated slower and the viral titers for YFV-Asibi cap0 were significantly lower, both on day three and on day seven post-injection, indicating that cap0 viral replication is also impaired in the secondary tissues.

## Discussion

Previous studies including our own showed that orthoflaviviruses evade the vertebrate innate immune system by methylating their capped viral genome at the first nucleotide via a 2’-O-MTase encoded in the non-structural protein NS5 resulting in a 5’ end cap1 structure [14,16,17,34,35]. Using the YFV vaccine strain (YFV-17D cap1) and a 2’-O-MTase deficient mutant (YFV-17D cap0), we initially showed that the replication of YFV-17D cap0 is impaired in vertebrate cells due to the recognition of the cap0 structure by RIG-I [17]. As an arbovirus, YFV needs the ability to replicate in both vertebrate and insect cells. Hence, conserved mechanisms might be involved in counteracting both the vertebrate and insect immune system. The mRNA cap structure is highly conserved among higher eukaryotes and therefore represents a rational target structure for discrimination of self and foreign RNA [14].

Our studies show that growth of the 2’-O-MTase deficient (cap0) YFV is inhibited in mosquito cells with the level of inhibition varying between different mosquito cells and also between different YFV strains. Interestingly, the level of inhibition was highest in the *Aedes albopictus*-derived C6/36 cells. While growth of the YFV cap0 vaccine strain was completely blocked, YFV-Asibi cap0 reached a titer of 10^4^ PFU/ml at ten days post-infection. In Aag2 cells, the cap0 variants of both YFV strains increased over time. However, replication of the cap0 vaccine strain was attenuated over the entire period compared to the cap1 counterpart. For YFV-Asibi, which grew faster overall, the inhibition of the cap0 virus was only seen at an early stage of infection.

Which factor (s) account(s) for the different inhibition levels of the YFV cap0 variants between the C6/36 and Aag2 cell lines still needs to be elucidated. One question is whether a cellular enzyme similar to the viral 2’-O-MTase might influence the different replication rates observed since variable methyltransferase levels have been described to influence arbovirus infection outcomes in mosquitoes. For example, it is known that expression of the endogenous cytosine methyltransferase DNMT2 is upregulated in *Wolbachia*-free *Aedes* mosquitoes upon infection with Sindbis virus resulting in higher replication rates [36]. Also, ectopic expression of DNMT2 in *Aedes albopictus* mosquito cells was found to be proviral, while the presence of *Wolbachia* down-regulated DNMT2 leading to reduced 5-methylcytosine modification levels of the viral RNA and blocking of viral replication [36]. For *Drosophila*, even a cap 2’-O-methyltransferase (dCMTR1) was described, which besides cap1 RNA methylation in the nucleus also acts as a positive regulator of the small RNA pathway associated with Ago2 and has a role in siRNA biogenesis and RISC assembly [37]. Hence, dCMTR1 was described to also be active in the cytoplasm. However, there was no evidence for 2’-O-methylation of the siRNAs in the cytoplasm [37]. This is in line with the finding that human CMTR1 bound to the polymerase II mediates 2’-O-methylation only co-transcriptionally in the nucleus [38]. Hence, although CMTR1 orthologs have been found both for *Aedes aegypti* and *Aedes albopictus* (accession no. XP_001652367.1 or XP_029712309.2, respectively), it is unlikely that they will modify the methylation status of the viral RNA in the cytoplasm.

As mentioned, the level of inhibition for the cap0 viruses was not only different in various mosquito cell lines but also between the different YFV strains, whereby YFV-Asibi cap0 was less inhibited than YFV-17D cap0. We aimed to pin down which viral protein(s) is/are responsible for the ability of YFV-Asibi cap0 to replicate to low levels in the C6/36 cells. Analyzing the growth rates of chimeras between YFV-17D and YFV-Asibi indicated that the structural proteins are not the crucial determinant mediating the cap0 virus growth. Rather, the NS proteins of YFV-Asibi in the backbone of YFV-17D allowed the cap0 chimera to grow to some extent. While Asibi NS5 in the YFV-17D background did not allow replication of the cap0 chimera, Asibi NS4A-5 mediated some growth. Still, also the 17D/Asibi NS4A-5 cap0 chimera did not reach a titer comparable to the YFV-Asibi cap0 virus. This suggests that an interplay of several Asibi proteins is necessary for increased cap0 virus growth, with major determinants localized in the NS4A-5 region. Further, an initial faster replication rate, as it was observed for YFV-Asibi cap1, seemed to allow the cap0 variants to replicate above the threshold in the later course of infection. While YFV-Asibi reached its peak titer of nearly 10^7^ PFU/ ml at three days post-infection, YFV-17D replicated only up to 10^5^ to 10^6^ PFU/ml in this time frame and reached its peak titer two days later. It might well be assumed that there is a link between the initial growth rate and the importance of the NS4A-5 region. With its function as an RNA polymerase, NS5 is one of the core proteins of the replication complex [39]. NS4A is known to be involved in membrane-remodeling and for certain orthoflaviviruses like West Nile virus (WNV) or Dengue virus (DENV) a role in formation of viral replication complexes (VRCs) or anchoring of the viral VRCs at the ER membrane has been described [40]. Also, NS4B has been suggested to play a role in viral RNA replication as it localizes with other NS proteins that are involved in viral replication [39]. Different replication kinetics were also observed for YFV-17D and YFV-Asibi in vertebrate cells. Here, the difference was described to be attributed to different entry mechanisms of the two YFV strains. While clathrin-mediated endocytosis was found to be the entry mechanism for YFV-Asibi, entry of YFV-17D relies on a clathrin-independent mechanism [41]. The more efficient entry mechanism of YFV-17D is linked to a more efficient infection of vertebrate cells. Due to this, the cytokine production is higher, which then limits the growth of YFV-17D [41]. Using virus replicon particles, for which a WNV replicon was packaged *in trans* with the YFV structural proteins (17D, Asibi or chimeric 17D/Asibi), it was shown that the envelope protein is responsible for the different entry mechanisms [41]. Since in our infection experiments the growth kinetics of the YFV-17D/Asibi SP virus rather resembled the YFV-17D than the YFV-Asibi phenotype, different entry mechanisms do not seem to be responsible for the faster growth of YFV-Asibi in the mosquito cells.

While we observed a complete (17D) or strong (Asibi) inhibition for the YFV cap0 mutants in C6/36 cells, attenuation in this cell line was not as pronounced for analogous 2’-O-MTase deficient orthoflaviviruses analyzed by others. For WNV, the viral titers of the cap0 variant (NS5-E218A) steadily increased within three days, remaining up to 1–2 log below the wild-type virus titer [11]. Attenuation of the cap0 variants was even less pronounced for DENV and Japanese encephalitis virus (JEV). The DENV cap0 mutant (NS5-E217A) was only slightly reduced by less than one log over a period of 5 days compared to its cap1 counterpart [16]. The JEV cap0 mutant (NS5-E218A) even showed reduced growth behavior only within the first two days and reached the titer of the wild-type cap1 virus after three days [42]. However, when comparing the results of the different mutants, it should be noted that different MOIs were used for the infection experiments. We primarily performed our growth curve analyses using an MOI of 0.01, while MOI 0.1 was used for the DENV and WNV experiments [11, 16] and MOI 1 for the JEV analyses [42]. Still, YFV-17D cap0 also did not grow within seven days when C6/36 cells were infected at an MOI of 0.1 (S8 Fig). In addition, while the titers for wild-type YFV-Asibi obtained in our studies are comparable to the titers others observed for wild-type DENV in C6/36 cells at three days post infection (about 5 x 10^6^ PFU/ml), we could not detect any growth for YFV-Asibi cap0 at that time point, while DENV cap0 was only slightly impaired compared to DENV cap1 [16]. This suggests that the different levels of attenuation for the different 2’-O-MTase deficient orthoflaviviruses not only depend on the infection dose or the speed of replication but also involves other factors. One possibility is that certain orthoflaviviruses express viral antagonists interfering with cap0 recognition.

In vertebrates, cap0 activates RIG-I resulting in upregulation of interferon stimulated genes (ISGs) [17]. Both RIG-I as well as Dcr-2 possess a DExD/H-box helicase domain involved in RNA binding [26]. Therefore, it was interesting to analyze whether in mosquito cells Dcr-2 is involved in cap0 recognition. The fact that inhibition of the YFV cap0 viruses was most striking in the C6/36 cells, which are known to lack functional RNAi response due to a truncated Dcr-2 [30], already suggested that the siRNA pathway or other Dcr-2 dependent antiviral pathways are not involved in the antiviral activity against the cap0 variant. Nevertheless, the truncated version in C6/36 cells still retains the RNA binding helicase domain in the N-terminal part of Dcr-2 and it could not be excluded that it is sufficient for the observed inhibition of YFV cap0 in C6/36 cells. We therefore compared the growth of YFV cap1 and cap0 in Aag2 wild-type cells and Aag2 Dcr-2 k.o. cells, for which CRISP/Cas9-mediated k.o. was performed using a guide RNA targeting exon1 of the Dcr-2 mRNA resulting in k.o. of all Dcr-2 domains [32].

If Dcr-2 would play a critical role in downregulating the cap0 viruses, the growth of the respective viruses should be increased in the Dcr-2 k.o. cells compared to the wild-type cells. However, for both YFV-17D and Asibi, the cap0 viruses grew to similar titers in wild-type and k.o. cells indicating that none of the Dcr-2 domains is involved in cap0 virus restriction. We also did not see a difference in viral titers for the cap1 viruses when comparing wild-type and k.o. cells. In contrast, others observed an increased replication rate for wild-type ZIKV in Dcr-2 k.o. cells [43]. The difference might be attributed to the fact that the YFV capsid protein functions as an antagonist of the RNAi pathway [44], while for the ZIKV capsid protein no anti-exogenic siRNA function was observed [43]. Interestingly, in contrast to our observation in cell culture, namely the comparable growth of YFV in Aag2 wild-type and Aag2 Dcr-2 k.o. cells, YFV replicated to higher titers in Dcr-2 null mosquitoes compared to wild-type mosquitoes [44]. A potential explanation might be that the RNAi response is less pronounced in Aag2 cells compared to living mosquitoes, allowing the YFV capsid protein to completely counteract and downregulate the RNAi response in Aag2 cells but not in mosquitoes.

Interestingly, analyses on single cell level elucidating the different growth of wild-type and 2’-O-MTase deficient DENV in immunocompetent vertebrate cells revealed that the attenuated phenotype of the mutant DENV results from an earlier induction of the interferon response and is not linked to reduced replication fitness of the mutant [45]. Together with mathematic modeling, it was further shown that attenuation of the DENV cap0 mutant is in first place determined by the kinetics of autocrine interferon action on infected cells whereas paracrine interferon action has a negligible impact [45]. In mosquito cells the secreted peptide Vago was described to act as a functional homolog of interferon restricting WNV infection by activation of the Jak-STAT pathway [46]. Upregulation of Vago was described for infection of *Culex quinquefasciatus* (Hsu) cells with WNV or *Aedes albopictus* (RML-12) cells with DENV [46]. In contrast, no evidence for induction of Vago1 and Vago2 was observed in Aag2 cells after infection with the insect-specific cricket paralysis virus (CrPV) or stimulation with dsRNA [47]. Hence, it was assumed that Vago expression after viral infection is not a consistent general response [47]. Nevertheless, activation of Vago was described to be dependent on Dcr-2 [46,48] and our studies indicate that attenuation of the cap0 viruses is Dcr-2-independent. This suggests that Vago does not play a critical role in attenuation of the YFV cap0 variants. Besides the siRNA pathway, of which Dcr-2 is a major component, the piRNA pathway represents another RNA-targeting pathway with putative antiviral activity [19]. In contrast to siRNAs, which have a length of 21 nt, piRNAs are 24–30 nt long. Further analyses involving comparative small RNA sequencing of YFV cap1 and YFV cap0 infected mosquito cells might elucidate, whether piRNAs play a role in YFV cap0 inhibition. In addition to the RNAi machinery, three major antiviral signal transduction pathways are known to regulate the antiviral defense in mosquitoes. This includes the JAK/STAT-, the Toll-, and the Immunodeficiency (IMD) pathway, which respond to viral infection by altering the expression of key immune genes including genes of multiple antimicrobial peptides (AMPs) [19]. Comparative transcriptome analysis might provide insights as to whether one of these pathways is involved in cap0 recognition.

In vertebrate cells it was shown that IFIT1 binds to cap0 RNA and competes with the cap-binding protein EIF4E for binding to RNA templates thereby specifically blocking translation of viral cap0 RNA [27]. Similarly, translation of cap0 RNA was also reduced in mosquito cells compared to the cap1 RNA as we could show using replication-incompetent full-length YFV-17D transcripts encoding luciferase. The different degrees of inhibition observed in different mosquito cells (average reduction factor for cap0 translation of 2.5 in C6/36 cells versus 1.8 in Aag2 cells) were in agreement with the data obtained from the viral infection experiments. For both, the cap0 virus growth curve analyses and the cap0 YFV RNA translation experiment, the inhibition was more profound in C6/36 cells compared to Aag2 cells. One explanation could be that a potential receptor or protein involved in recognizing the cap0 structure is expressed to a different extent in different mosquito cells. Whether mosquitoes possess a receptor or protein able to discriminate between cap1 and cap0 similar to RIG-I or IFIT1 in vertebrates and whether it is constitutively expressed or upregulated upon infection like ISGs in vertebrates remains to be investigated. In *Drosophila melanogaster* two cGAS-like receptors were identified, which are activated in response to infection with RNA or DNA viruses [49, 50]. However, cGAS is not present in mosquitoes [51]. Similarly, based on a BLAST search, no IFIT1 ortholog or orthologs of other IFIT proteins such as IFIT2, 3, or 5 are found in mosquitoes. This leaves the question open as to whether another RNA recognizing protein, which otherwise plays a role in RNA modification or RNA stability, for example, is able to bind to the cap0 structure.

Although the detailed mechanism of attenuation for the YF cap0 viruses still needs to be elucidated, the results of the *in vivo* experiments show that the 2’-O-MTase deficient YFV is impaired at the midgut barrier level as well as in secondary tissues. While the viral titers assessed via viral RNA copies steadily increased after an eclipse phase for YFV-Asibi cap1 within three weeks after oral infection, no viral RNA or only decreasing RNA levels were observed for YFV-Asibi cap0 over time in the carcasses (mosquitoes w/o legs and wings). Further, in contrast to YFV-Asibi cap1, only few mosquitoes were positive for viral RNA in legs plus wings after oral feeding suggesting a role of the midgut barrier for the reduced infection rates observed for the cap0 variant.

With regard to the midgut barrier one can distinguish between midgut infection barrier (MIB) and midgut escape barrier (MEB). Both the MIB and the MEB as well as the salivary gland infection barrier (SGIB) and saliva gland escape barrier (SGEB) represent physical barriers that strongly affect the vector competence for a particular arbovirus [52, 53]. Overcoming the MIB and MEB is a prerequisite for dissemination to take place. For orthoflaviviruses such as DENV, it was suggested that the MIB is the major determinant of vector competence [54]. Also for the YFV-17D vaccine strain, midgut barriers were described to prevent the replication and dissemination of the virus in *Aedes aegypti* mosquitoes [29]. That the midgut barrier is also involved in the inhibition of the cap0 variant could more clearly be shown by analyzing isolated midguts. While the YFV-Asibi cap1 virus readily replicated in the midguts of the mosquitoes, the YFV-Asibi cap0 infected mosquitoes had a lower median viral midgut titer, with no virus being detectable in numerous midguts.

A MIB can either result from the fact that a virus cannot enter the epithelial midgut cells or from the fact that the virus is unable to replicate or spread after entering [52]. While the first possibility most likely is determined by virus-specific midgut epithelial receptors, the second possibility is rather linked to the initiation of innate immunity-related pathways. Since binding to the receptors occurs via the structural proteins which are not different between YFV Asibi-cap1 and -cap0, initiation of innate immune-related pathways is likely to be the factor involved in the reduced viral titers observed for the YFV cap0 virus in the midguts.

Efficient replication in the midgut is a prerequisite for dissemination. Hence, the reduced replication levels of YFV-Asibi cap0 in the midguts most likely contribute to the lower dissemination rates observed. Sequencing analyses of 16 samples that were positive for viral RNA in legs plus wings revealed that, except for one sample in which the wild type sequence was observed, all still contained the mutations encoding the NS5 E218A exchange. This indicates, that the 2’-O-MTase deficient virus is indeed able to disseminate to secondary tissues to some extent. However, if YFV-cap0 is able to overcome the midgut barrier and reaches the hemolymph, its replication is again impaired in the secondary tissues as was seen when analyzing legs plus wings after intrathoracic infection. Hemocytes, of which 75% are circulating, represent the cellular arm of the mosquito immune response [53,55]. Among others, they have been described to function in pathogen-associated molecular pattern (PAMP) recognition and hence might also be involved in recognizing the cap0 structure. Overall, it will be interesting to see whether YFV cap0 infection initiates a pathway that also affects levels of vector competence for other arboviruses.

As mentioned before, replication of DENV cap0 was only slightly reduced in C6/36 cells [16]. Still, Züst et al. described that the DENV cap0 variant was unable to infect *Aedes aegypti* mosquitoes after oral feeding, whereas for DENV cap1 24% of the mosquitoes were infected using the highest infection dose of 10^5^ PFU/ml [16]. The infection dose seemed to be very critical for a successful DENV infection of the mosquitoes, as reducing the dose by one or two log already resulted in a drop of infection rate to 1% or 6%, respectively [16]. Consequently, since the authors did not assess viral load shortly after oral feeding, it is difficult to really compare the infection rates between DENV cap1 and cap0 in mosquitoes, also because fewer cap0 infected mosquitoes than cap1 infected mosquitoes were examined. Likewise, viral growth after intrathoracic injection of the two DENV variants could have been assessed better if the viral load had been determined directly after the injection. Nevertheless, viral replication seemed to occur for both DENV cap1 and cap0 after intrathoracic infection, with cap1 growing to higher titers than cap0 [16]. The fact that the replication of YFV-Asibi cap0 was impaired in both the midguts after oral infection and in legs plus wings after intrathoracic infection indicates that a potential receptor or protein recognizing the cap0 structure is localized in the midgut as well as in secondary tissues. Since recognition of cap0 occurs in a Dcr-2-independent manner, our data suggest the existence of an innate 5’ RNA-modification recognizing effector protein in mosquitoes beyond the well-characterized siRNA pathway. Further elucidating the molecular virus-immune interactions involved in recognizing YFV cap0 in mosquitoes will be useful to establish methods preventing transmission of arboviruses thereby reducing the global arboviral disease burden.

## Materials and methods

### Cell culture

A549 (kindly provided by Stephan Günther, Hamburg, Germany) and Vero B4 (kindly provided by Ute Winke, Bonn, Germany) cells were cultured in DMEM (Gibco, Thermo Fisher) containing 10% FBS (Sigma-Aldrich). Baby Hamster Kidney cells (BHK-21/J, kindly provided by Charles M. Rice, Rockefeller University, New York, NY, USA) were cultured in MEM (Gibco, Thermo Fisher) containing 7.5% FBS, 1% L-glutamine (Gibco, Thermo Fisher), and 1% non-essential amino acids (NEAA, Gibco, Thermo Fisher). The mosquito-derived cell lines were maintained in Leibovitz’s L-15 medium (Gibco, Thermo Fisher), either supplemented with 10% FBS (C6/36, CRL-1660, ATCC) or with 10% FBS, 10% tryptose phosphate broth solution (Gibco, Thermo Fisher), and 1% NEAA (Aag2-AF5 [56] and Aag2-derived Dcr-2 k.o. Aag2-AF319 [32] cell lines, kindly provided by Kevin Maringer, University of Surrey, United Kingdom). All vertebrate cells were kept at 37°C and 5% CO_2_, and the insect cells at 28°C.

### Plasmid construction

To establish YFV-17D cap0, the NS5 E218A mutation was introduced into the pACNR/FLYF-17Dx infectious clone (pYFV-17D ic) [57] (kindly provided by Charles M. Rice, Rockefeller University, New York, NY, USA) by fusion PCR technology. To this end, two PCR fragments were amplified from pYFV-17D ic using the primers Bo795 (5’-CAATGACAATAGTCATGCTG-3’) and Bo1308 (5’-GCTCCAGACACGTAGTACATG**GC**ATGAGTGGAATTCCTGG-3’) or Bo1307 (5’-CCAGGAATTCCACTCATG**CC**ATGTACTACGTGTCTGGAGC-3’) and Bo721 (5’- TCAACAAAGCCACGTTGTGT -3’). The exchanged nucleotides are marked in bold. The generated PCR fragments were fused with primers Bo795 and Bo721 and the resulting fragment was cut with the enzymes NgoMIV and XhoI. Afterwards, the fragment was ligated into the pYFV-17D ic cut with the same enzymes. Introduction of the desired mutations and absence of unwanted mutations in the cloned PCR fragment were verified via Sanger sequencing.

The YFV-Asibi infectious clone (pYFV-Asibi ic) was established based on a precursor YFV-Asibi* clone kindly provided by Charles M. Rice, Rockefeller University, New York, NY, USA. Nucleotide exchanges in the 3’ UTR corresponding to YFV-17D, three mutations resulting in amino acid exchanges, and silent mutations still present in the precursor YFV-Asibi* clone were exchanged using fusion PCR technology combined with classical cloning. The resulting sequence of the YFV-Asibi clone (pYFV-Asibi ic) corresponds to the YFV-Asibi sequence published by Hahn and colleagues [58] except for one nucleotide which was not exchanged (nt position 2142 ccording to amino acids 714) (S2 Table). The primers used for fusion PCRs and details on cloning are available upon request.

The corresponding YFV-Asibi E218A mutant was constructed as follows: two PCR fragments were amplified from the pYFV-Asibi ic using the primers Bo795 and Bo1308 or Bo1307 and Bo1181 (CATATGGCACGGCTTCCCTTTGC). The amplified PCR fragments were fused with the outer primers Bo795 and Bo1181 and cut with the enzymes NgoMIV and AatII. The resulting fragment was ligated into the pYFV-Asibi ic, cut with the same enzymes. The plasmid was sequenced up to and across cloning junctions to verify the accuracy of the desired mutations and the absence of unwanted mutations.

For establishing the YFV-17D/Asibi chimeras, the pYFV-17D ic and the pYFV-Asibi ic were used as the backbones for all constructs. The different chimeras were constructed using PCR-based mutagenesis combined with classical cloning via enzyme restriction sites. The PCR reactions were performed using the Phusion High-Fidelity polymerase (Thermo Fisher Scientific) according to the manufacturer’s protocol. The primers and details on cloning are available upon request.

To establish a replication incompetent YFV-17D reporter virus expressing Renilla luciferase (YFV-17D Rluc_AAG), an MluI-XhoI fragment was cut out from a plasmid encoding a YFV-17D Rluc replicon, in which the polymerase activity was knocked out via mutation (GDD-AAG) [59]. The respective fragment was inserted into a previously described plasmid encoding a Renilla luciferase expressing YFV-17D virus (YF_Ren) [60]. To establish YFV-17D Rluc_E218A_AAG, an MluI-XhoI fragment was excised from a YFV-17D reporter replicon plasmid, which contains mutations for both the E218A and GAA-AAG exchanges, and inserted into YF_Ren cut with the same restriction enzymes.

### *In vitro* transcription and recovery of recombinant virus

All infectious cDNA clones were linearized with *XhoI*, and *in vitro* transcription was performed using the mMESSAGE mMACHINE SP6 Transcription Kit (Invitrogen, Thermo Fisher Scientific) according to the manufacturer’s instructions. The transcription efficiency was verified by gel electrophoresis in an ethidium bromide gel. Recombinant viruses were recovered by electroporating the *in vitro* transcribed RNA into BHK-J/21 cells as previously described [59].

### Growth kinetic studies in cell culture

Cells were seeded 24 h before infection at a density of 2.5 x 10^5^ cells/ well (C6/36, Aag2), 1 x 10^5^ cells/ well (A549), or 8 x 10^4^ cells/ well (Vero, BHK-21/J) in a 24-well plate. Infection was performed at a multiplicity of infection (MOI) of 0.01 for 1 h at 28°C for insect cells or 37°C and 5% CO_2_ for vertebrate cells. After infection, the cells were washed twice with PBS and once with medium without supplements before adding the corresponding cell culture medium. Supernatant containing the virus was collected at the indicated time points. The aliquots were stored at -80°C until further use. Viral titers were determined via plaque assay.

### Infectious center assay and plaque assay

For infectious center assays, tenfold serial dilutions of transfected BHK-21/J cells were mixed with 5 x 10^5^ untransfected cells and seeded in 6-well plates. After attachment for 4 to 6 h, the medium was removed and cells were covered with 3 ml overlay containing 1.2% agarose (Biozym Scientific GmbH) mixed 1:1 with 2x MEM (Bio&Sell) supplemented with 4% FBS and 2% Pen/Strep (Gibco, Thermo Fisher). The cells were incubated for 3 days at 37°C and 5% CO_2_, fixed with 6% formaldehyde, and stained with crystal violet (0.2% crystal violet (Merck Millipore) in 20% EtOH). Viral titers were determined by plaque assay on BHK-21/J cells. The cells were seeded 24 h prior to titration at a density of 3 x 10^5^ cells/ well in 6-well plates. Cell monolayers were infected with 200 μl tenfold serial dilutions of the cell culture supernatant prepared in PBS containing 1% FBS. Following inoculation for 1 h at 37°C, cells were overlaid with agarose, incubated for three days, fixed, and stained as described above.

### DsRNA production and transfection of nucleic acids

T7 promotor-flanked primers were used to perform PCR reactions on the gene of interest. To amplify a gene section of the Renilla luciferase (Rluc) gene, primers Bo1356 (GTAATACGACTCACTATAGGGGTGGTGGGCTCGCTGCAAGCAAATG) and Bo1357 (GTAATACGACTCACTATAGGGGGACTCGATCACGTCCACGACACTC) were used. Amplification of a GFP gene region was performed using primers Bo1267 (GTAATACGACTCACTATAGGGATGGTGAGCAAGGGCGAGGAGCTG) and Bo1268 (GTAATACGACTCACTATAGGGCCTCCTTGAAGTCGATGCCCTTCAG). The resulting PCR fragments were used as template for *in vitro* transcription using the MEGAscript RNAi kit (Thermo Fisher Scientific) to produce the respective dsRNAs. After DNAse I and RNase digestion of the produced dsRNAs, co-transfection of 200 ng dsRNA with 200 ng of a Rluc expressing plasmid (pIZ-Rluc) was performed using DharmaFECT2 transfection reagent (Horizon). Rluc read out was performed at 24 h using the Renilla Luciferase Assay System (Promega).

### Capping and methylation of *in vitro* transcribed RNA

The replication-deficient YFV reporter constructs were linearized with XhoI, and 2 μg of linearized DNA were used in an *in vitro* transcription reaction using the SP6-Scribe Standard RNA IVT Kit according to the manufacturer’s protocol (Cellscript, Wisconsin, USA). After DNase I treatment, the transcription product was purified by ammonium acetate precipitation using 5 M ammonium acetate as described by the manufacturer. To modify the *in vitro* transcripts with either a 5’ cap1 or a 5’ cap0 structure, the ScriptCap Cap1 Capping System (Cellscript, Wisconsin, USA) was used according to the manufacturer’s protocol.

### Reporter YFV genome transfection

C6/36 or Aag2 cells were electroporated with 3 μg *in vitro* transcripts of replication-incompetent YFV-17D genomes carrying either a 5’ cap1 or 5’ cap0 structure. Electroporation of the mosquito cells was performed as described previously for C6/36 cells [61]. The electroporated cells were seeded into 24 wells at a concentration of 5 x 10^5^ per well. At the indicated time points, cells were either lysed in 100 μl 1 x Lysis Juice (PJK Biotech, Kleinblittersdorf, Germany) for Renilla luciferase measurement or resuspended in 500 μl TRIzol (Thermo Fisher Scientific) for RNA isolation. 20 μl cell lysate were used to determine the luciferase levels with the Gaussia-Juice Luciferase Assay Kit (PJK Biotech, Kleinblittersdorf, Germany) using a Junior LB 9509 device (Berthold Technologies, Bad Wildbad, Germany). RNA isolation and measurement of YFV genomes were performed as described below.

### Mosquito rearing

The *Aedes aegypti* Liverpool strain was kindly provided by Sanjay Basu (Pirbright Institute, UK). The mosquitoes were kept under standardized conditions at 28°C and 80% humidity in a 12:12 h day/night cycle. For synchronized hatching, filter papers with eggs were placed in a desiccator, and vacuum was applied for 1 h. Following hatching, 300–400 first instar larvae were collected and placed in a tray with 1 l deionized water. Larvae and pupae were fed with crushed nutritionally balanced fish food (TetraMin) once a day. Hatched pupae were counted and transferred to new 30 cm x 30 cm x 30 cm cages (width x depth x height). Emerging adult mosquitoes were kept in the same cages as the pupae and were maintained with 10% sucrose solution *ad libitum*. Twice a week, mosquitoes were fed with sheep blood (defibrinated, supplemented with 5 mM ATP (Sigma-Aldrich)) using a Hemotek membrane feeding device to maintain the colony.

### Infection of mosquitoes

For oral and intrathoracic infections, pupae were collected for 5–7 days to obtain the closest emerging date possible. Female mosquitoes were separated from the males 48 h prior to the experiments. Up to 20 females were collected per plastic tray covered with mesh. On average, the mosquitoes were 7–14 days old on the day of infection or injection. Mosquitoes reared for *in vivo* experiments were fed with 10% sucrose twice a week. After viral infection feeding with 10% sucrose was performed daily. All *in vivo* infection experiments were performed in a BSL-3 laboratory. To allow mosquitoes to acclimate to the negative pressure in the BSL-3, they were moved to the laboratory at least 48 h before the experiment.

For oral infections, mosquitoes were starved for 24 hours and were then allowed to feed on defibrinated sheep blood (Fiebig, Xebios Diagnostics GmbH) supplemented with the respective virus for 1 hour. Feeding was performed using a Hemotek membrane feeding device (Hemotek Ltd.). The blood meal had a total volume of 3 ml and consisted of 50% blood and 50% virus supplemented with 5 mM ATP (Sigma-Aldrich). The final virus concentration was 1 x 10^7^ PFU/ml. Following feeding, mosquitoes were anesthetized on ice and visually inspected for blood uptake. Engorged mosquitoes were transferred to new cylindrical containers covered with mesh.

Intrathoracic injection was performed according to Rosen and Gubler [62]. Briefly, the mosquitoes were anesthetized on ice, and single mosquitoes were arranged on their site. The viral solution containing 300 PFU virus in 27.6 nl 0.9% sodium chloride solution was injected into the mosquito thorax. The injection was performed with fine glass capillaries using a NanoJect II device (Drummond Scientific Company). Injected mosquitoes were transferred to new cylindrical containers covered with mesh.

Following infection or injection, mosquitoes were kept under standardized conditions at 28°C and 80% humidity in a 12:12 h day/night cycle. They were monitored daily and fed with 10% sucrose.

### Mosquito processing

Orally infected mosquitoes were anesthetized using ice and placed under a Leica DMS1000 microscope (Leica, Wetzlar, Germany) for dissection into legs, wings, and carcass. In case the midgut was prepared, the carcass was separated into thorax and abdomen and the midgut was separated from the other organs. Dissection was performed within 3 h post-infection or on days 3, 5, 7, 10, 14, or 21. Legs and wings of single mosquitoes were pooled and placed in tubes filled with 8 to 10 1.4 mm zirconium oxide beads (Bertin Technologies, Montigny-le-Bretonneux, France). The carcasses (either mosquito w/o legs and wings or mosquito w/o legs, wings, and midgut, as indicated) and midguts were also placed in tubes filled with 8 to 10 1.4 mm zirconium oxide beads. Intrathoracic infected mosquitoes were dissected into legs plus wings and the remaining carcass and samples were placed in tubes filled with 8 to 10 1.4 mm zirconium oxide beads at 0, 3 or 7 days post-infection. All samples were stored at -80°C until further use.

### Nucleic acid extraction and real-time RT-PCRs

For RNA extraction from mosquitoes, the samples were thawed and mosquitoes or mosquito body parts were mixed with 300 μl MEM medium without supplements. The tubes were placed into a tissue lyser adaptor and stored at -20°C for 8 min before the samples were shredded twice for 30 sec with a frequency of 30 Hz using a TissueLyser (Qiagen, Hilden, Germany). Subsequently, the samples were centrifuged at 2,500 rpm for 10 min at 4°C. For RNA isolation, 140 μl supernatant was mixed with 300 μl RAV1 buffer (NucleoSpin RNA virus kit, Macherey-Nagel, Macherey-Nagel, Düren, Germany). The samples were heated at 70°C for 10 min before the RNA was extracted according to the manufacturer’s instructions except for the first step. Due to the reduced amount of RAV1 buffer, the samples were mixed with 300 μl 100% EtOH before loading onto a column. The viral RNA was eluted in 60 μl H_2_O and stored at -80°C until further use.

Viral genome copies were quantified by real-time RT-PCR (SuperScript III One-Step RT-PCR System with Platinum Taq DNA Polymerase, Invitrogen) using primers Bo1590 (5’-TCCCTGAGCTTTACGACCAGA-3’) and Bo1591 (5’-AATCGAGTTGCTAGGCAATAAACAC-3’) and the probe Bo1592 (5’-6-carboxyfluorescein FAM/ATCGTTCGT/ZEN/ TGAGCGATTAGCAG/3IABkFQ-3‘). Each 20 μl reaction contained 3 μl RNA, 1 x Reaction Mix, 2.5 mM additional MgSO_4_, 0.25 μM each of the primers and the probe, and 0.4 μl SuperScript III RT/Platinum Taq Mix. RT-PCR started with reverse transcription for 30 min at 45°C and pre-denaturation at 95°C for 5 min, followed by 45 cycles of denaturation at 95°C for 10 sec, and annealing-extension at 57°C for 30 sec. Ten-fold dilutions of *in vitro* transcribed RNA from the target region served as a standard for each separate run. Samples were run in a LightCycler 480 Instrument II (Roche, Basel, Switzerland).

To determine YFV genome copies normalized to the *Aedes aegypti* housekeeping gene RPS7 in electroporated cells, RNA extraction was performed using TRIzol (Thermo Fisher Scientific) according to the manufacturer’s instructions. The RNA isolated from a 24 well was dissolved in 25 μl of water, and 4 μl RNA were analyzed in a multiplex RT-qPCR using the SuperScript III One-Step RT-PCR System with Platinum Taq DNA Polymerase. In addition to the 4 μl RNA, each 20 μl reaction contained besides the reaction components described above for the YFV RT-qPCR 0.125 μM of primers Bo1882 (5’-AGATGAACTCGGACCTGAAG-3’) and Bo1883 (5’ GGGACGTAGATCACGATAGC-3’), as well as 0.125 μM of the probe Bo1884 (5’-/5HEX/CGTGATCTG/ZEN/TACATCACCCGCGCT/3IABkFQ/-3’). The reaction was run on a LightCycler 480 Instrument II using the program described above. Serial ten-fold dilutions of *in vitro* transcribed RNAs from both target regions served as standards. In addition, standard curves were used to validate primer efficacies for both the YFV and RPS7 reactions.

### Statistics

Statistical significance was determined with GraphPad Prism 10 (GraphPad Software Inc., San Diego, CA, USA). Comparison of translation and stability of YFV genomes as well as differences of viral replication in intrathoracically infected mosquitoes were calculated using the Mann-Whitney test. To evaluate statistical significance of differences for viral replication in vertebrate cells, the unpaired t-test was used. For all tests a *p* < 0.05 was considered significant (* p ≤ 0.05; ** p ≤ 0.01; *** p ≤ 0.001; **** p ≤ 0.0001; ns: not significant).

## Supporting information

S1 Fig Replication of YFV-17D cap1 and cap0 in different insect cells. (A) Growth of YFV-17D cap1 and cap0 in U4.4 cells. (B) Growth of YFV-17D cap1 and cap0 in CCL-125 cells. Cells were infected at a multiplicity of infection (MOI) of 0.01 and viral titers were measured at day 5 post-infection by titration on BHK cells. Data represent Mean ± SD of triplicates. Dashed lines: detection limit.
(TIF)

S2 Fig Replication of YFV-Asibi cap1 and cap0 in different vertebrate cells. (A) Growth of YFV-Asibi cap1 and cap0 in BHK cells. (B, C) Growth of YFV-Asibi cap1 and cap0 A549 wild-type and IFIT k.o. cells. Cells were infected at a multiplicity of infection (MOI) of 0.01, and viral titers were measured at day 3 post-infection by titration on BHK cells. Data represent Mean ± SD of five replicates (BHK) or triplicates (A549). Unpaired t-test was used to calculate statistical significance (* p ≤ 0.05). Dashed lines: detection limit.
(TIF)

S3 Fig Growth kinetics of YFV-17D/Asibi chimeras in C6/36 cells. The colors represent the sequence source (green: YFV-17D; yellow: YFV-Asibi), and shared sequences are those that do not differ between Asibi and 17D (grey). The red star represents the E218A exchange in the NS5 protein. A) Growth kinetics of 17D/Asibi NS1-5 cap1 and cap0 in C6/36 cells. B) Growth kinetics of 17D/Asibi NS1-4B cap1 and cap0 in C6/36 cells. C) Growth kinetics of 17D/Asibi NS4A** cap1 and cap0 in C6/36 cells. D) Growth kinetics of 17/Asibi 2K-NS4B cap1 and cap0 in C6/36 cells. The cells were infected in triplicates at an MOI of 0.01. Viral titers were measured at 0, 3, 5, 7, and 10 days post-infection by titration on BHK cells. Data represent Mean ± SD of triplicates. Dashed lines: detection limit.
(TIF)

S4 Fig Chimera and wild-type virus replication at early time points. (A) Replication of the different 17D/Asibi cap1 chimeras three days post-infection. The chimeras are grouped into clusters depending on the replication ability of the corresponding cap0 chimera. On the left are the cap1 chimeras where the cap0 chimera exceeded the detection limit, and on the right are the cap1 chimeras where the cap0 chimera did not exceed the detection limit. (B) Replication of YFV-Asibi cap1 and YFV-17D cap1 at early time points post-infection. C6/36 cells were infected at an MOI of 0.01. Viral titers were measured at 0, 12, 24, 48, and 72 hours post-infection by titration on BHK-21/J cells. Data represent Mean ± SD of triplicates. Dashed lines: detection limit.
(TIF)

S5 Fig Validation of Dcr-2 knockout in the Aag2 319 cell line. The parental Aag2 AF5 cell line and the Dcr-2 k.o. cell line Aag2 AF319 were co-transfected with a Rluc expressing construct (pIZ-Rluc) and dsRNA against the Rluc gene (dsRluc). Co-transfection of dsRNA against the eGFP gene (dsGFP) was used as control. At 24 h post transfection cells were lysed to determine the Rluc expression levels.
(TIF)

S6 Fig Translation efficiency of a replication-incompetent cap0 reporter YFV genome is reduced in Aag2 cells. Aag2 cells were electroporated with cap1 or cap0 reporter YFV-17D genome transcripts expressing Rluc. At 4 h, 8 h, and 22 h post-electroporation, cells were lysed to measure Rluc activity. The means ± SD of n = 3 independent experiments each analyzed with three biological replicates are shown. Individual values are visualized as dots. The Mann-Whitney test was used to calculate statistical significance (**** p ≤ 0.0001, *** p ≤ 0.001 ns = not significant).
(TIF)

S7 Fig Determination of infectious viral particles after oral infection. A subset of female mosquitoes shown in Fig 5 that were orally fed with an infectious blood meal containing 1 x 10^7^ PFU/ml virus was analyzed for infectious particles. Infectious virus particles in the mosquitoes’ carcacasses (A) and legs plus wings (B) were determined by titration on BHK cells. The blue line indicates the median titers. Dashed line: detection limit.
(TIF)

S8 Fig Replication of YFV-17D cap1 and cap0 in C6/36 cells. Growth of YFV-17D cap1 and cap0 in C6/36 cells. Cells were infected at a multiplicity of infection (MOI) of 0.1 and viral titers were measured at the indicated time points by titration on BHK cells. Data represent Mean ± SD of duplicates. Dashed lines: detection limit.
(TIF)

S1 Table Infection and dissemination rates for YFV-Asibi cap1 and cap0 at different time points post-infection. Infection rate: mosquitoes positive in the carcasses (entire mosquito without legs and wings) in relation to the total number of examined mosquitoes; dissemination rate: mosquitoes containing viral RNA in legs plus wings in relation to the number of positive carcasses.
(PDF)

S2 Table Summary of differences between pYFV-17D and pYFV-Asibi. Amino acids in bold indicate aa exchanges between YFV-17D and YFV-Asibi.
(PDF)

S1 Data Raw data obtained during the study. (PDF)
