# Supplementary figures and images for: 2’-O-methyltransferase-deficient yellow fever virus: Restricted replication in the midgut and secondary tissues of Aedes aegypti mosquitoes severely limits dissemination
Source: PLoS Pathog. 2024 Oct 2;20(10):e1012607. doi: 10.1371/journal.ppat.1012607 (PMC11472933; doi:10.1371/journal.ppat.1012607)

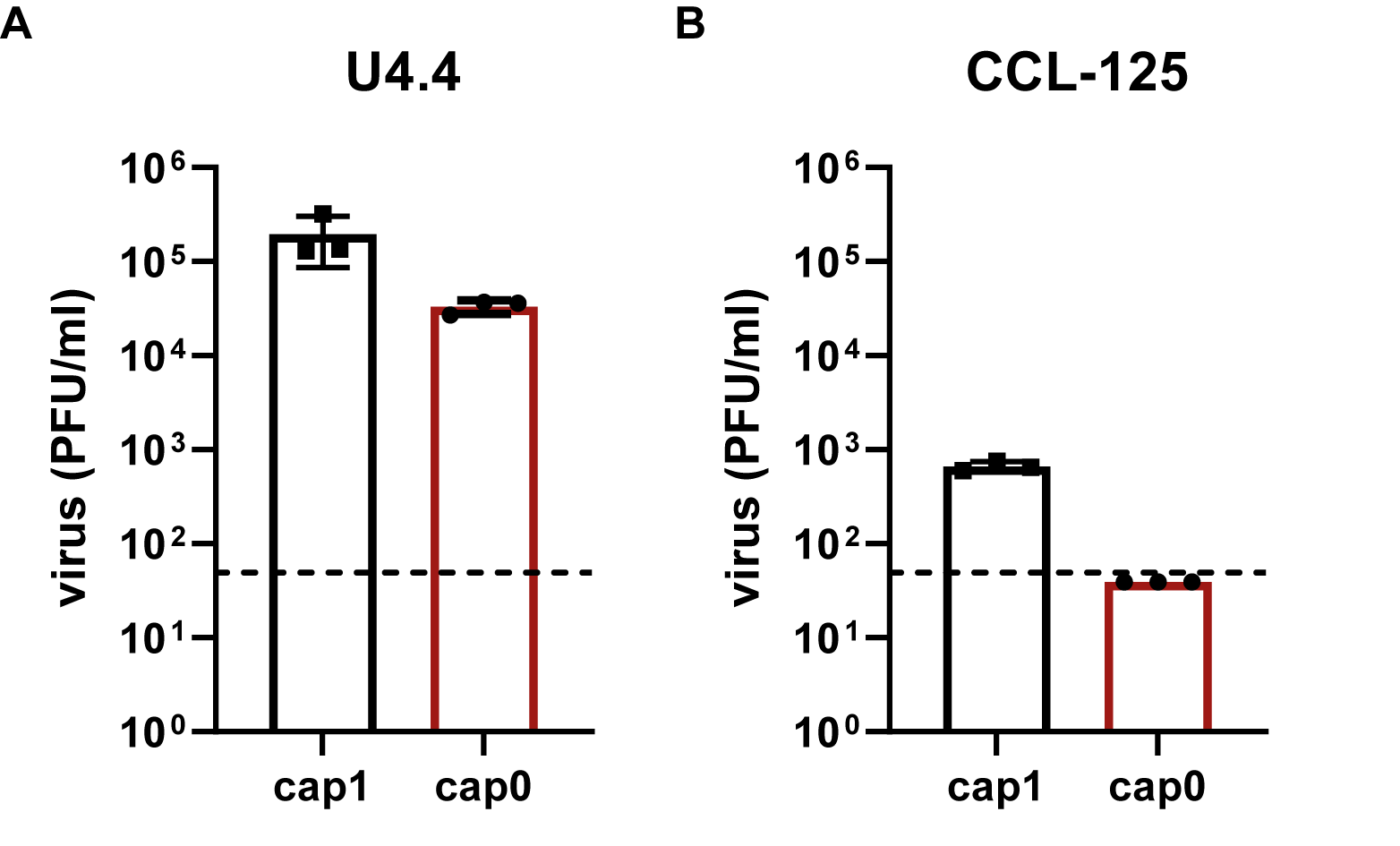

Supplement: S1 Fig — (A) Growth of YFV-17D cap1 and cap0 in U4.4 cells. (B) Growth of YFV-17D cap1 and cap0 in CCL-125 cells. Cells were infected at a multiplicity of infection (MOI) of 0.01 and viral titers were measured at day 5 post-infection by titration on BHK cells. Data represent Mean ± SD of triplicates. Dashed lines: detection limit. (TIF) [file ppat.1012607.s001.tif]

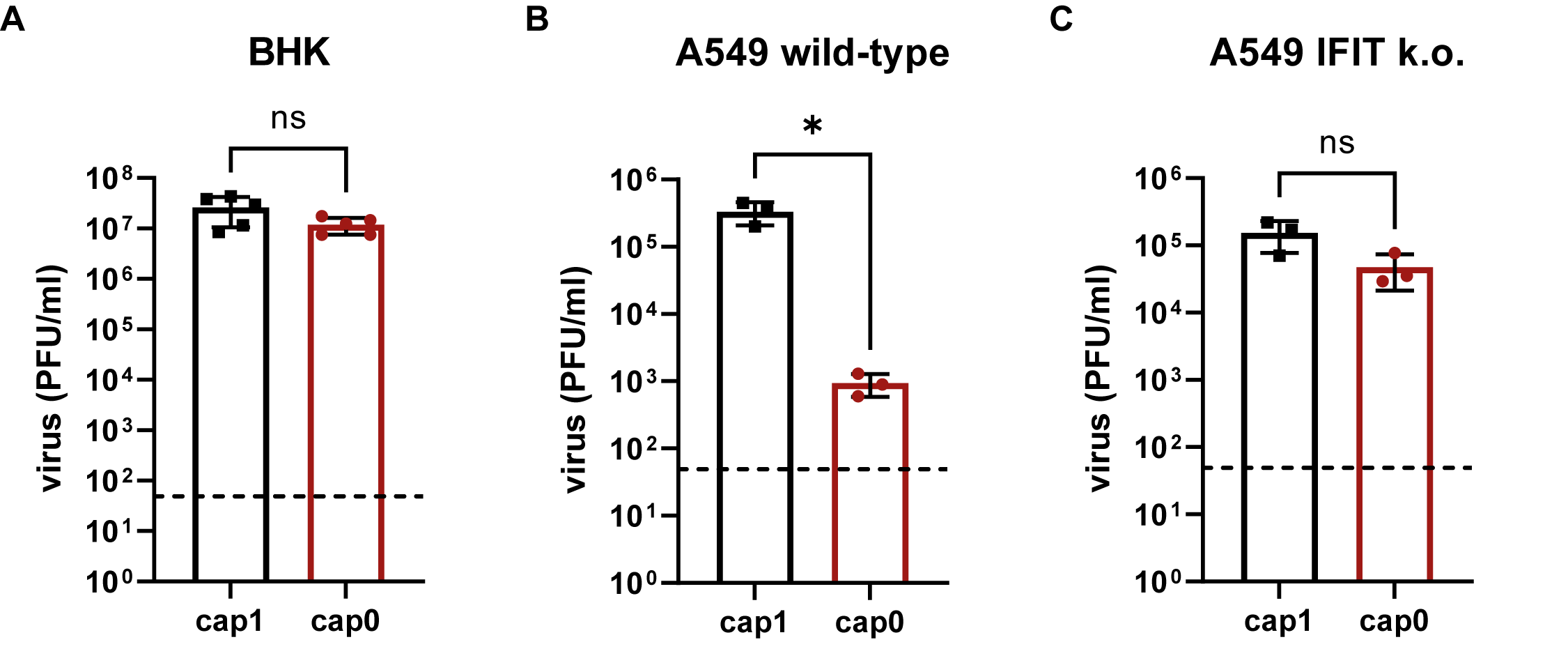

Supplement: S2 Fig — (A) Growth of YFV-Asibi cap1 and cap0 in BHK cells. (B, C) Growth of YFV-Asibi cap1 and cap0 A549 wild-type and IFIT k.o. cells. Cells were infected at a multiplicity of infection (MOI) of 0.01, and viral titers were measured at day 3 post-infection by titration on BHK cells. Data represent Mean ± SD of five replicates (BHK) or triplicates (A549). Unpaired t-test was used to calculate statistical significance (* p ≤ 0.05). Dashed lines: detection limit. (TIF) [file ppat.1012607.s002.tif]

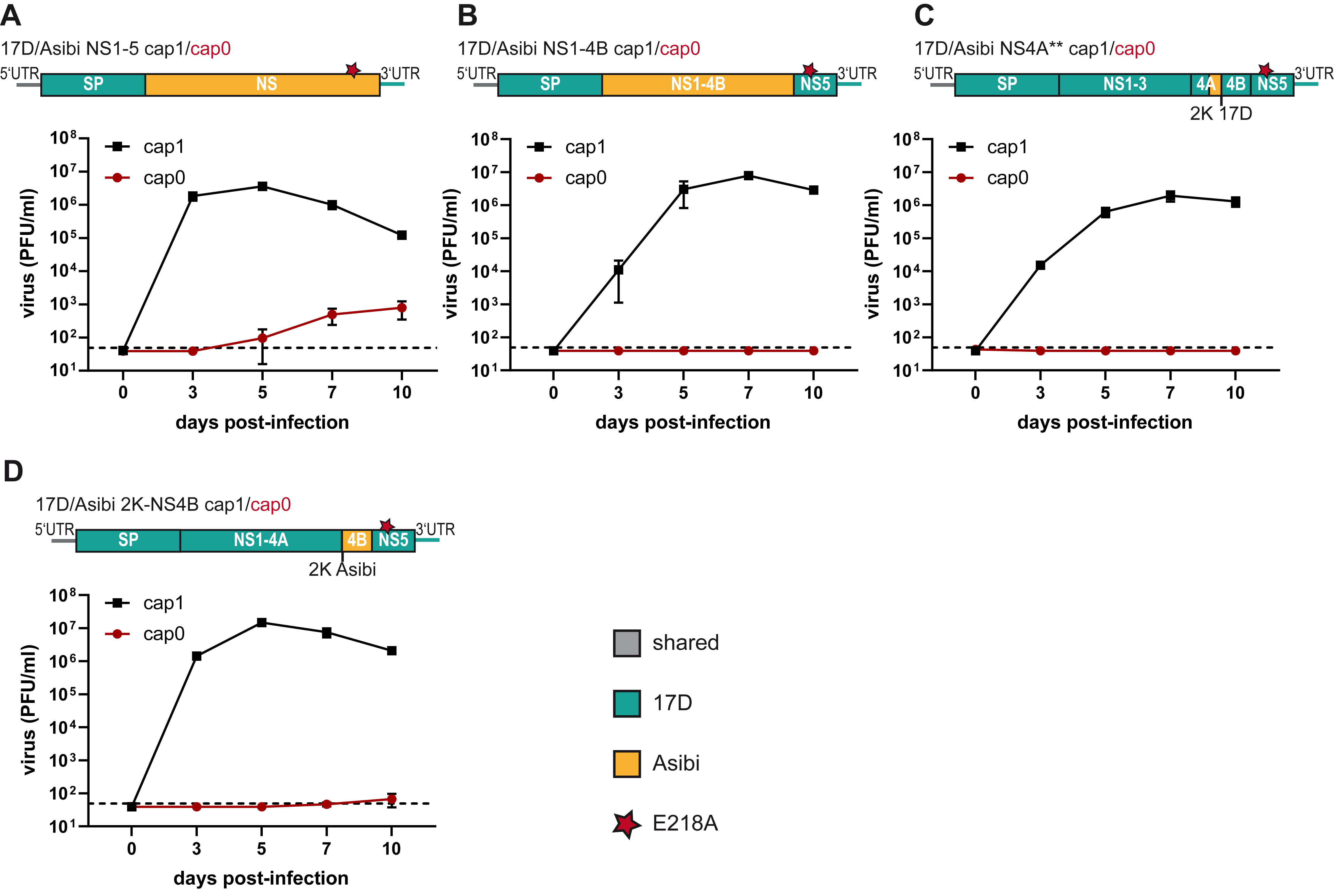

Supplement: S3 Fig — The colors represent the sequence source (green: YFV-17D; yellow: YFV-Asibi), and shared sequences are those that do not differ between Asibi and 17D (grey). The red star represents the E218A exchange in the NS5 protein. A) Growth kinetics of 17D/Asibi NS1-5 cap1 and cap0 in C6/36 cells. B) Growth kinetics of 17D/Asibi NS1-4B cap1 and cap0 in C6/36 cells. C) Growth kinetics of 17D/Asibi NS4A** cap1 and cap0 in C6/36 cells. D) Growth kinetics of 17/Asibi 2K-NS4B cap1 and cap0 in C6/36 cells. The cells were infected in triplicates at an MOI of 0.01. Viral titers were measured at 0, 3, 5, 7, and 10 days post-infection by titration on BHK cells. Data represent Mean ± SD of triplicates. Dashed lines: detection limit. (TIF) [file ppat.1012607.s003.tif]

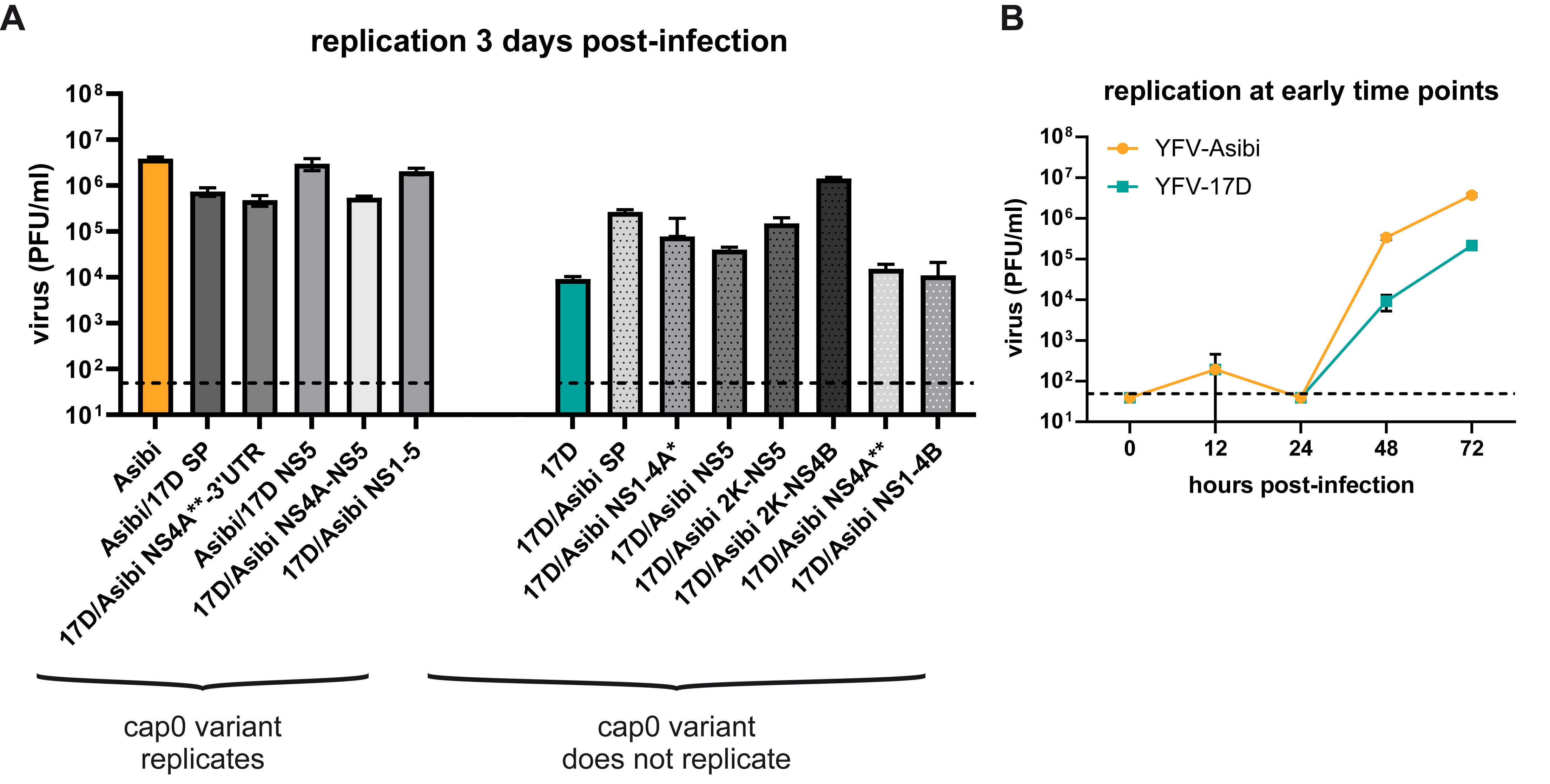

Supplement: S4 Fig — (A) Replication of the different 17D/Asibi cap1 chimeras three days post-infection. The chimeras are grouped into clusters depending on the replication ability of the corresponding cap0 chimera. On the left are the cap1 chimeras where the cap0 chimera exceeded the detection limit, and on the right are the cap1 chimeras where the cap0 chimera did not exceed the detection limit. (B) Replication of YFV-Asibi cap1 and YFV-17D cap1 at early time points post-infection. C6/36 cells were infected at an MOI of 0.01. Viral titers were measured at 0, 12, 24, 48, and 72 hours post-infection by titration on BHK-21/J cells. Data represent Mean ± SD of triplicates. Dashed lines: detection limit. (TIF) [file ppat.1012607.s004.tif]

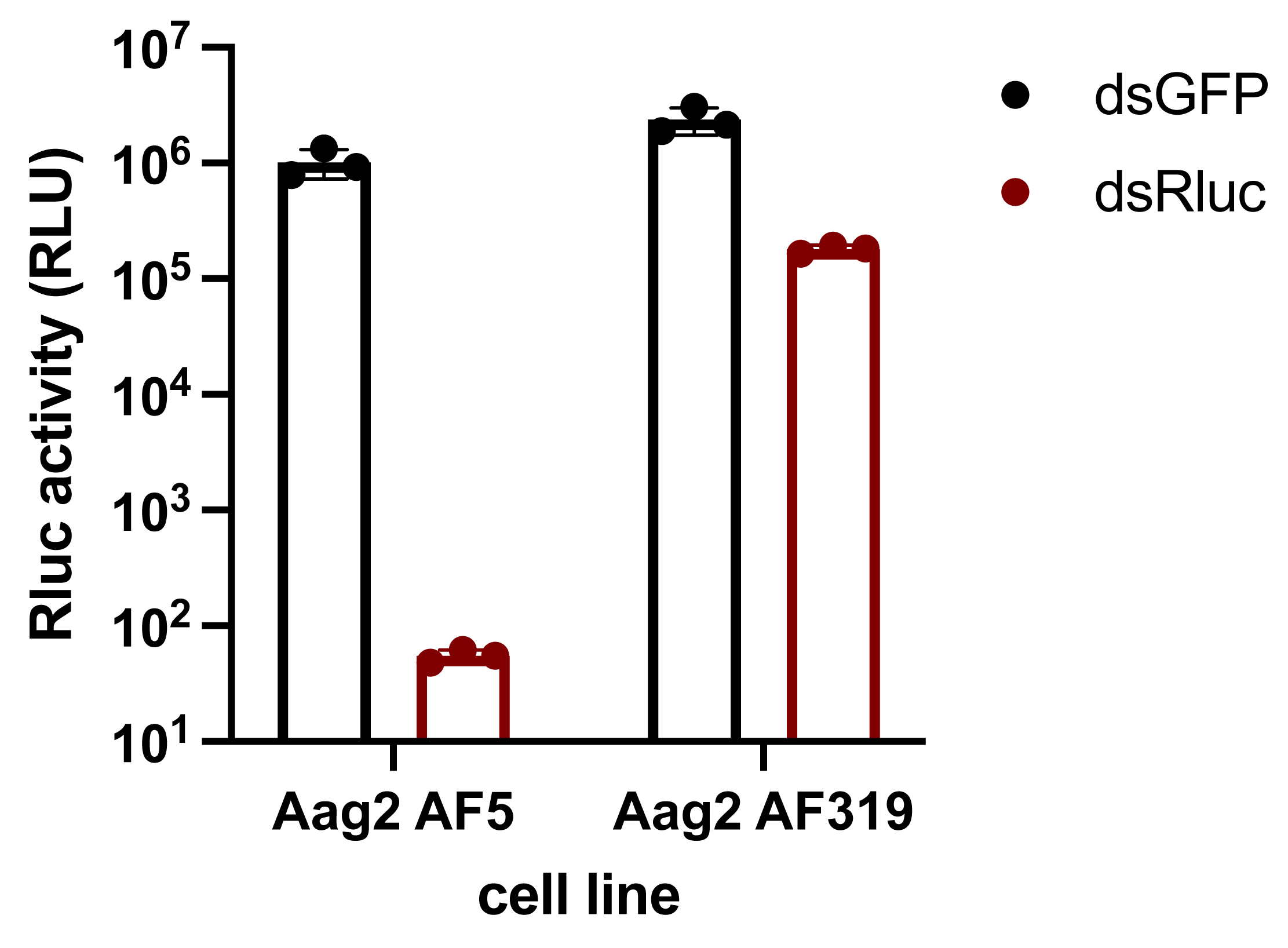

Supplement: S5 Fig — The parental Aag2 AF5 cell line and the Dcr-2 k.o. cell line Aag2 AF319 were co-transfected with a Rluc expressing construct (pIZ-Rluc) and dsRNA against the Rluc gene (dsRluc). Co-transfection of dsRNA against the eGFP gene (dsGFP) was used as control. At 24 h post transfection cells were lysed to determine the Rluc expression levels. (TIF) [file ppat.1012607.s005.tif]

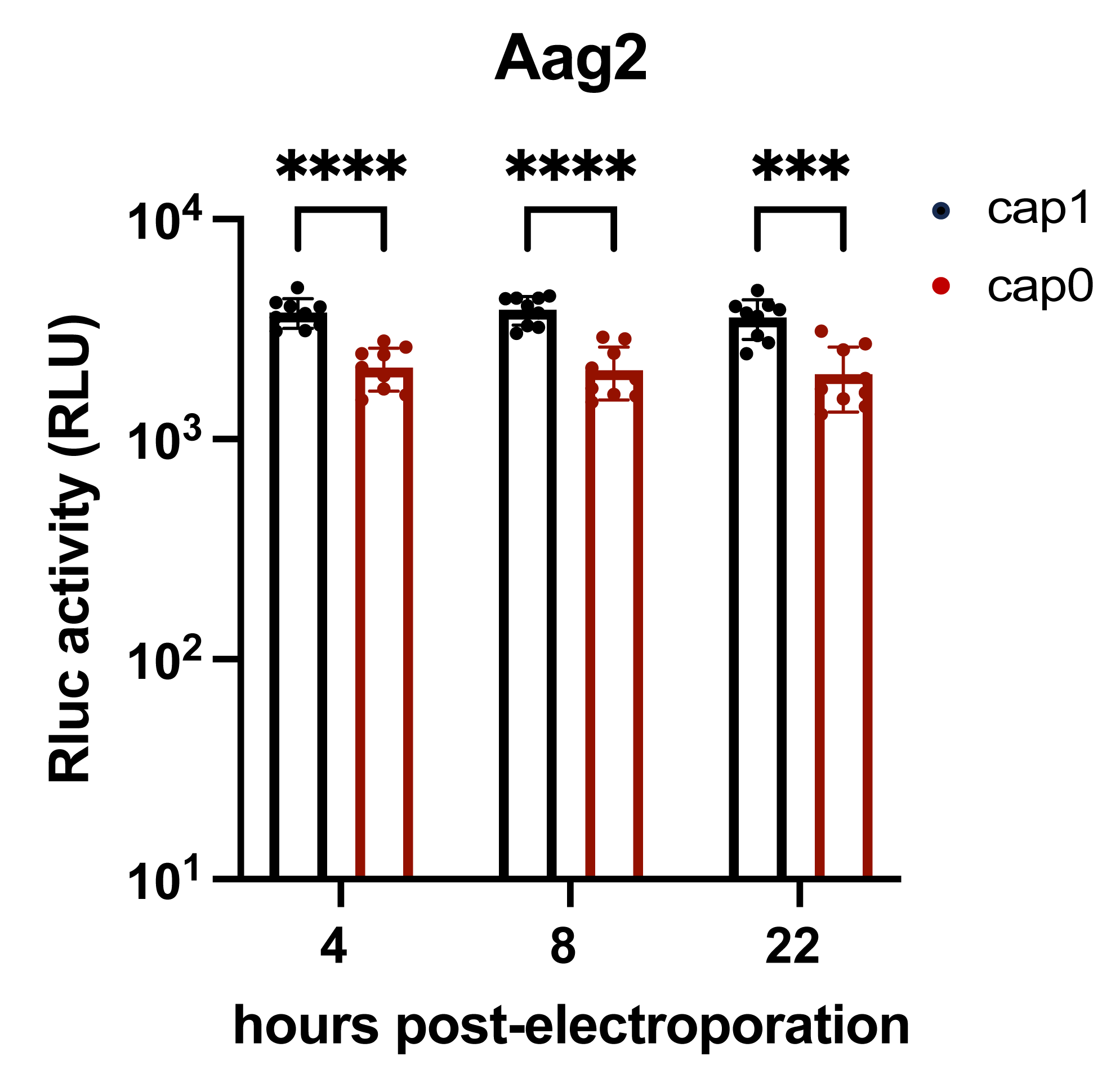

Supplement: S6 Fig — Aag2 cells were electroporated with cap1 or cap0 reporter YFV-17D genome transcripts expressing Rluc. At 4 h, 8 h, and 22 h post-electroporation, cells were lysed to measure Rluc activity. The means ± SD of n = 3 independent experiments each analyzed with three biological replicates are shown. Individual values are visualized as dots. The Mann-Whitney test was used to calculate statistical significance (**** p ≤ 0.0001, *** p ≤ 0.001 ns = not significant). (TIF) [file ppat.1012607.s006.tif]

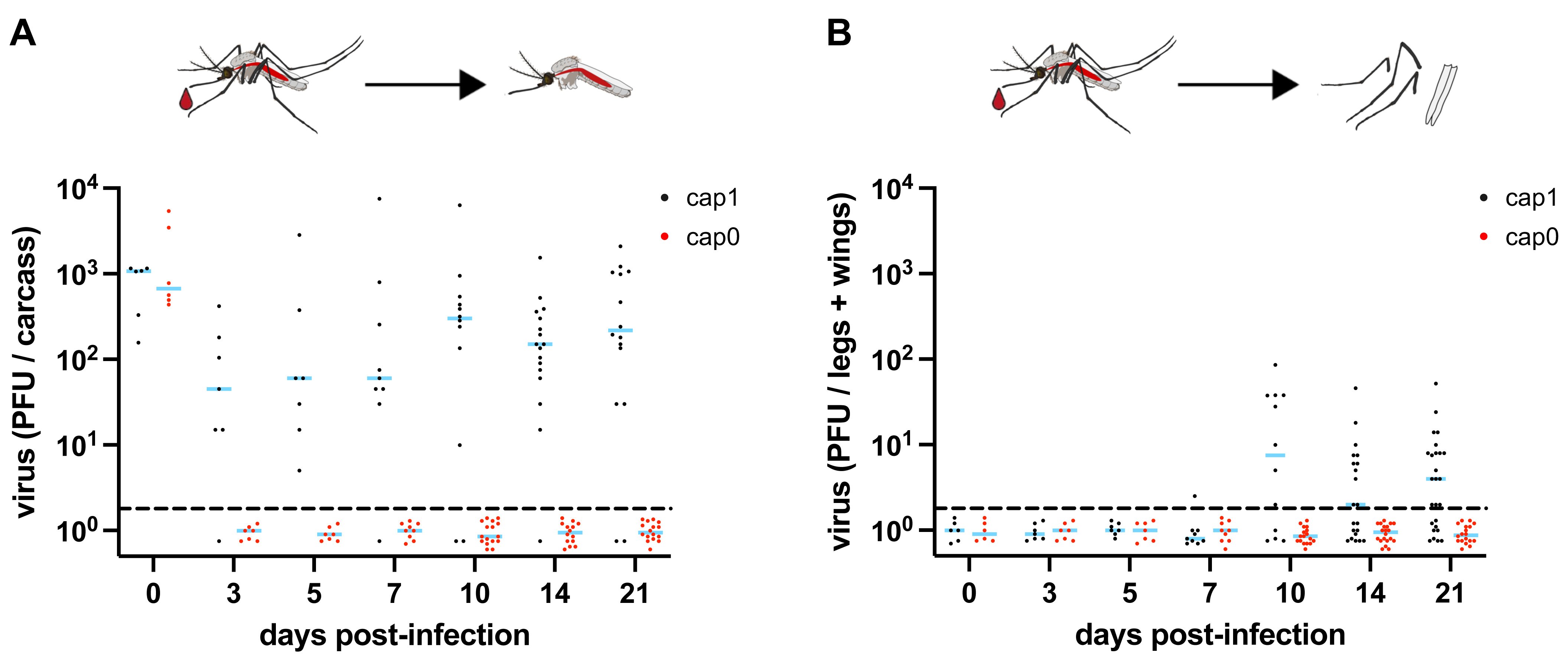

Supplement: S7 Fig — A subset of female mosquitoes shown in Fig 5 that were orally fed with an infectious blood meal containing 1 x 10^7^ PFU/ml virus was analyzed for infectious particles. Infectious virus particles in the mosquitoes’ carcacasses (A) and legs plus wings (B) were determined by titration on BHK cells. The blue line indicates the median titers. Dashed line: detection limit. (TIF) [file ppat.1012607.s007.tif]

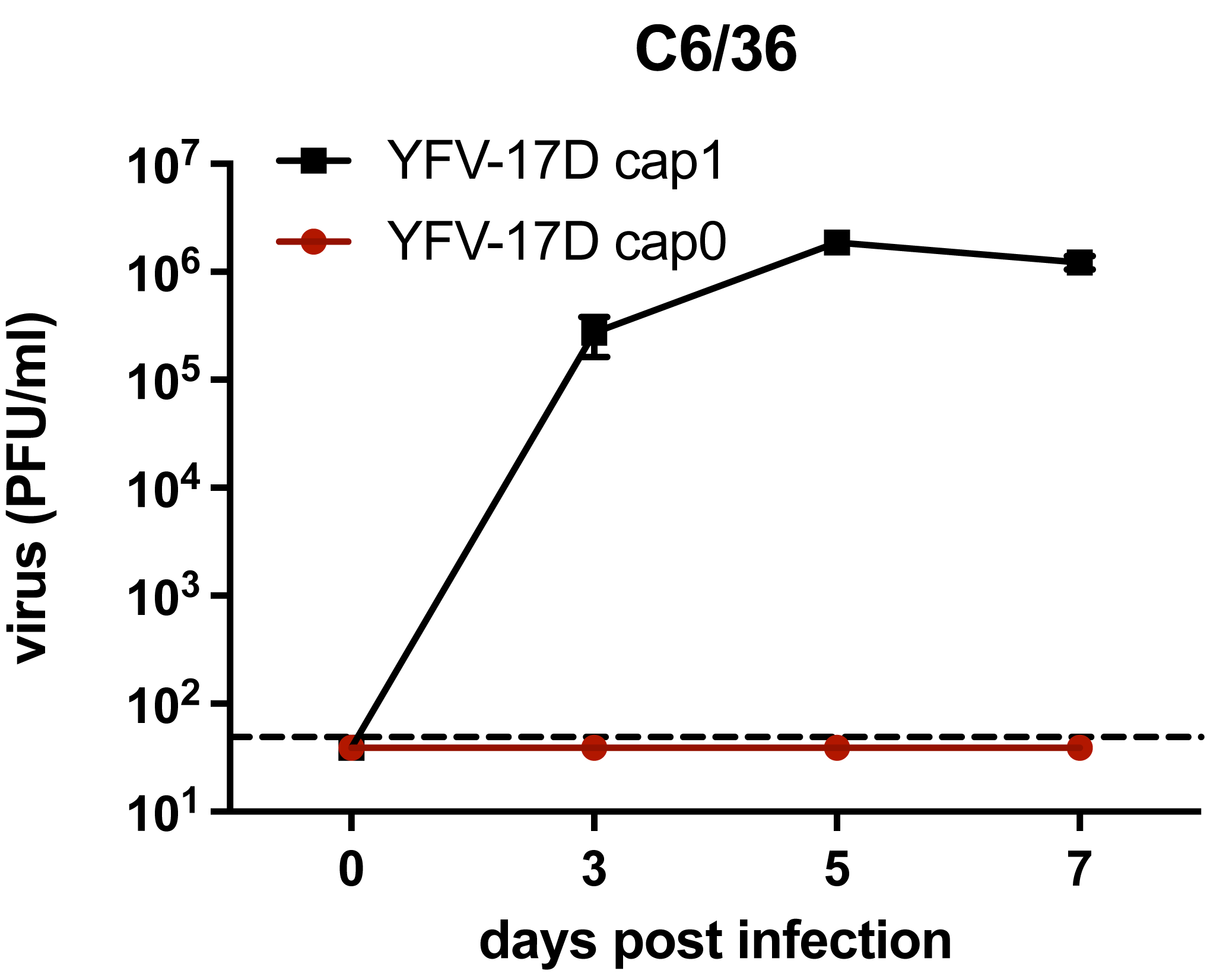

Supplement: S8 Fig — Growth of YFV-17D cap1 and cap0 in C6/36 cells. Cells were infected at a multiplicity of infection (MOI) of 0.1 and viral titers were measured at the indicated time points by titration on BHK cells. Data represent Mean ± SD of duplicates. Dashed lines: detection limit. (TIF) [file ppat.1012607.s008.tif]
