# Supplementary material for: 2’-O-methyltransferase-deficient yellow fever virus: Restricted replication in the midgut and secondary tissues of Aedes aegypti mosquitoes severely limits dissemination
Source: PLoS Pathog. 2024 Oct 2;20(10):e1012607. doi: 10.1371/journal.ppat.1012607 (PMC11472933; doi:10.1371/journal.ppat.1012607)
Supplement: S1 Table — Infection rate: mosquitoes positive in the carcasses (entire mosquito without legs and wings) in relation to the total number of examined mosquitoes; dissemination rate: mosquitoes containing viral RNA in legs plus wings in relation to the number of positive carcasses. (PDF) [file ppat.1012607.s009.pdf]

S1 Table: Infection and dissemination rates for YFV-Asibi cap1 and cap0 at different time points post-infection.

Infection rate: mosquitoes positive in the carcasses (entire mosquito without legs and wings) in relation to the total number of examined mosquitoes; dissemination rate: mosquitoes containing viral RNA in legs plus wings in relation to the number of positive carcasses.

virus	days post-infection	infection rate (%)	dissemination rate (%)
cap1	3	53/54<br>98%	14/53<br>26%
	5	50/53<br>94%	18/50<br>36%
	7	53/55<br>96%	35/53<br>66%
	10	55/57<br>96%	49/55<br>89%
	14	44/45<br>98%	37/44<br>84%
	21	44/45<br>98%	38/44<br>86%
cap0	3	11/33<br>33%	2/11<br>18%
	5	6/31<br>19%	0/6<br>0%
	7	6/30<br>20%	1/6<br>17%
	10	7/30<br>23%	1/7<br>14%
	14	7/31<br>23%	2/7<br>29%
	21	9/37<br>24%	1/9<br>11%
