# Supplementary material for: 2’-O-methyltransferase-deficient yellow fever virus: Restricted replication in the midgut and secondary tissues of Aedes aegypti mosquitoes severely limits dissemination
Source: PLoS Pathog. 2024 Oct 2;20(10):e1012607. doi: 10.1371/journal.ppat.1012607 (PMC11472933; doi:10.1371/journal.ppat.1012607)
Supplement: S2 Table — Amino acids in bold indicate aa exchanges between YFV-17D and YFV-Asibi. (PDF) [file ppat.1012607.s010.pdf]

S2 Table. Summary of differences between pYFV-17D and pYFV-Asibi. Amino acids in bold indicate aa exchanges between YFV-17D and YFV-Asibi.

Protein	Position	nt exchange		aa in the respective virus	
		YFV-17D	YFV-Asibi	YFV-17D	YFV-Asibi
C	304	A	G	T	T
	370	C	T	V	V
prM	854	T	C	**F**	**L**
	883	G	A	T	T
E	1127	A	G	**R**	**G**
	1140	T	C	**V**	**A**
	1482	T	C	**V**	**A**
	1491	T	C	**I**	**T**
	1572	C	A	**T**	**K**
	1750	T	C	T	T
	1819	T	C	S	S
	1870	A	G	**I**	**M**
	1887	T	C	**F**	**S**
	1946	T	C	**S**	**P**
	1965	G	A	**R**	**K**
	2112	G	C	**R**	**T**
	2219	A	G	**T**	**A**
	2356	T	C	L	L
NS1	2687	T	C	**F**	**L**
	2704	G	A	V	V
	3274	A	G	E	E
	3371	G	A	**V**	**I**
NS2A	3613	A	G	V	V
	3860	G	A	**V**	**M**
	4007	G	A	**A**	**T**
	4013	T	C	**F**	**L**
	4022	G	A	**A**	**T**
	4054	T	C	**N**	**N**
	4056	T	C	**F**	**S**
NS2B	4289	C	A	**L**	**I**
	4387	G	A	G	G
	4505	C	A	**L**	**I**
	4507	C	T	L	L

S2 Table: Summary of differences between pYFV-17D and pYFV-Asibi (continued).

Protein	Position	nt exchange		aa in the respective virus	
		YFV-17D	YFV-Asibi	YFV-17D	YFV-Asibi
NS3	4612	C	T	I	I
	4873	G	T	A	A
	5153	G	A	**V**	**I**
	5194	C	T	F	F
	5431	T	C	I	I
	5473	T	C	A	A
	5641	A	G	L	L
	6013	T	C	P	P
	6023	A	G	**N**	**D**
NS4A	6448	T	G	A	A
	6529	C	T	F	F
	6758	G	A	**V**	**I**
2K	6829	C	T	D	D
	6876	C	T	**A**	**V**
NS4B	7171	G	A	**M**	**I**
	7571	A	C	R	R
	7580	C	T	**H**	**Y**
NS5	7642	C	T	S	S
	7701	G	A	**R**	**Q**
	7945	T	C	F	F
	8008	C	T	I	I
	8629	T	C	Y	Y
	9605	G	A	**D**	**N**
	10142	A	G	**K**	**E**
	10243	A	G	L	L
	10285	C	T	Y	Y
	10312	G	A	R	R
	10338	T	C	**L**	**P**
3'UTR	10367	C	T	-	-
	10418	C	T	-	-
	10454	G	A	-	-
	10550	C	T	-	-
	10800	A	G	-	-
	10847	C	A	-	-
