# Supplementary material for: 2’-O-methyltransferase-deficient yellow fever virus: Restricted replication in the midgut and secondary tissues of Aedes aegypti mosquitoes severely limits dissemination
Source: PLoS Pathog. 2024 Oct 2;20(10):e1012607. doi: 10.1371/journal.ppat.1012607 (PMC11472933; doi:10.1371/journal.ppat.1012607)
Supplement: S1 Data — (PDF) [file ppat.1012607.s011.pdf]

Fig. 1B

C6/36 cells

days	YFV-17D cap0			YFV-17D cap1		
0	39	39	39	39	39	39
3	39	39	39	340000	320000	325000
5	39	39	39	16000000	7500000	9500000
7	39	39	39	7050000	5850000	6350000
10	39	39	39	1850000	1600000	2400000

39 = below detection limit

Fig. 1C

Aag2 cells

days	YFV-17D cap0			YFV-17D cap1		
0	39	39	39	39	39	39
3	650	700	400	1700	1800	4000
5	5000	7500	4400	85000	110000	50000
7	16500	15500	14000	1000000	550000	950000
10	110000	90000	120000	1650000	750000	160000

39 = below detection limit

Fig. 1E

C6/36 cells

days	YFV-Asibi cap0			YFV-Asibi cap1		
0	39	39	39	39	39	39
3	39	39	39	4200000	3550000	3750000
5	600	450	600	2900000	2400000	3150000
7	2100	2100	2050	1400000	1750000	1450000
10	14500	13500	15500	165000	115000	180000

39 = below detection limit

Fig. 1F

Aag2 cells

days	YFV-Asibi cap0			YFV-Asibi cap1		
0	39	39	39	39	39	39
3	10500	8500	8500	120000	165000	65000
5	95000	55000	110000	150000	100000	220000
7	90000	30000	165000	185000	85000	85000
10	95000	75000	115000	70000	125000	170000

39 = below detection limit

Fig. 2A

days	cap1					cap0				
0	39	39	39	50	39	39	39	39	39	39
3	600000	700000	900000	710000	625000	39	39	39	39	100
5	4400000	4050000	4450000	3750000	5850000	39	39	50	100	50
7	1300000	900000	900000	2850000	3300000	200	50	350	300	500
10	150000	85000	75000			600	150	150	1450	700

39 = below detection limit

Fig. 2B

days	cap1					cap0				
0	39	39	39	39	39	39	39	39	39	39
3	235000	300000	260000	250000	315000	39	39	39	50	39
5	1650000	2100000	2150000	3400000	3550000	39	39	36	50	39
7	1000000	750000	750000	2500000	3550000	39	50	39	39	39
10	265000	295000	275000			39	39	39	39	100

39 = below detection limit

Fig. 2C

days	cap1			cap0		
0	39	39	39	39	39	39
3	9500	11000	210000	39	39	39
5	4000000	4300000	20000000	39	39	39
7	12500000	13500000	6500000	39	39	100
10	2850000	4650000	2350000	39	39	39

39 = below detection limit

Fig. 2D

days	cap1						cap0					
0	50	39	39	39	39	39	39	39	39	39	39	39
3	1500000	1650000	1750000	485000	600000	350000	39	39	50	39	39	39
5	6250000	6550000	6000000	7300000	4600000	5100000	39	39	100	50	50	50
7	2150000	2150000	1850000	2850000	1500000	2250000	50	100	150	39	39	300
10	270000	205000	295000	290000	315000	420000	50	50	250	100	100	300

39 = below detection limit

Fig. 2E

days	cap1			cap0		
0	39	39	39	39	39	39
3	41000	34000	44500	39	39	39
5	2550000	1850000	2400000	39	39	39
7	3100000	3250000	2050000	39	39	100
10	500000	750000	650000	39	39	39

39 = below detection limit

Fig. 2F

days	cap1						cap0					
0	39	39	39	39	39	39	39	39	39	39	39	39
3	1200000	1650000	1700000	3300000	2000000	3650000	50	39	50	39	39	39
5	2550000	2550000	1750000	3900000	3050000	3100000	100	50	250	39	200	150
7	375000	535000	510000	2150000	1650000	2150000	600	200	600	800	500	500
10	130000	255000	290000	290000	260000	300000	500	800	1250	1750	1300	1500

39 = below detection limit

Fig. 2G

days	cap1			cap0		
0	39	39	39	39	39	39
3	200000	145000	105000	39	39	39
5	4250000	3850000	4750000	50	50	39
7	3150000	3850000	3150000	39	39	39
10	1400000	800000	1450000	39	39	39

39 = below detection limit

Fig. 2H

days	cap1			cap0		
0	39	39	39	39	39	39
3	490000	580000	560000	39	39	39
5	4650000	5650000	6200000	100	100	50
7	2300000	2550000	2350000	200	250	150
10	265000	450000	355000	39	300	450

39 = below detection limit

Fig. 3A

days	YFV-17D cap0			YFV-17D cap1		
0	39	39	39	39	39	39
3	650	700	400	1700	1800	4000
5	5000	7500	4400	85000	110000	50000
7	16500	15500	14000	1000000	550000	950000

39 = below detection limit

Fig. 3B

days	YFV-17D cap0			YFV-17D cap1		
0	39	39	39	39	39	39
3	900	450	550	1700	2900	2700
5	1800	1400	2100	60000	60000	120000
7	8000	6000	9000	750000	1200000	1000000

39 = below detection limit

Fig. 3C

days	YFV-Asibi cap0			YFV-Asibi cap1		
0	39	39	39	39	39	39
3	10500	8500	8500	120000	165000	65000
5	95000	55000	110000	150000	100000	220000
7	90000	30000	165000	185000	85000	85000

39 = below detection limit

Fig. 3D

days	YFV-Asibi cap0			YFV-Asibi cap1		
0	39	39	39	39	39	39
3	800	1450	1200	115000	29000	45000
5	35000	65000	75000	195000	205000	200000
7	22000	10500	31500	150000	130000	180000

39 = below detection limit

Fig. 4B

C6/36

hours	cap1								
4	149895	151208	167783	175976	141513	172123	162090	185781	188631
8	140755	157157	121779	151128	163575	155137	156039	145299	152346
22	120860	107940	106072	152511	136796	111652	108407	102704	129991

hours	cap0								
4	57866	69185	66536	84721	74190	71883	70643	67529	74481
8	53062	47852	52372	69883	61517	79874	60368	59252	60565
22	34406	36066	36573	71853	64365	64609	51253	46460	49095

p value

<0,0001
<0,0001
<0,0001

Fig 4C

YFV genome copies

Experiment: 2024.07.23 wdh cap1 and cap0 probes with YF-Standard and RPS7-Standard Selected Filter: FAM (465-510)

Include	Color	Pos	Name	Cp	Concentration	Standard	Status
True	255	A1	4h EP#195/3 870 cap1 3.4	22.63	2.94E5	0	
True	255	A2	4h EP#195/3 870 cap1 3.5	22.36	3.54E5	0	
True	255	A3	4h EP#195/3 870 cap1 3.6	24.78	6.63E4	0	
True	255	A4	4h EP#195/4 871 cap0 4.4	29.33	2.83E3	0	
True	255	A5	4h EP#195/4 871 cap0 4.5	26.35	2.24E4	0	
True	255	A6	4h EP#195/4 871 cap0 4.6	26.82	1.61E4	0	
True	255	A7	8h EP#195/3 870 cap1 3.4	21.02	8.99E5	0	E - Extrapolated concentration in standard curve
True	255	A8	8h EP#195/3 870 cap1 3.5	20.93	9.53E5	0	E - Extrapolated concentration in standard curve
True	255	A9	8h EP#195/3 870 cap1 3.6	21.42	6.81E5	0	E - Extrapolated concentration in standard curve
True	255	A10	8h EP#195/4 871 cap0 4.4	21.14	8.23E5	0	E - Extrapolated concentration in standard curve
True	255	A11	8h EP#195/4 871 cap0 4.5	20.84	1.01E6	0	E - Extrapolated concentration in standard curve
True	255	A12	8h EP#195/4 871 cap0 4.6	21.48	6.52E5	0	E - Extrapolated concentration in standard curve
True	255	B1	22h EP#195/3 870 cap1 3.4	24.82	6.43E4	0	
True	255	B2	22h EP#195/3 870 cap1 3.5	25.12	5.22E4	0	
True	255	B3	22h EP#195/3 870 cap1 3.6	22.56	3.08E5	0	
True	255	B4	22h EP#195/4 871 cap0 4.4	25.17	5.07E4	0	
True	255	B5	22h EP#195/4 871 cap0 4.5	25.32	4.55E4	0	
True	255	B6	22h EP#195/4 871 cap0 4.6	30.33	1.41E3	0	
True	65280	B7	4h EP#195/5 C6/36 ZK 5.4			0	
True	65280	B8	22h EP#195/5 C6/36 ZK 5.4			0	
True	65280	B9	MM			0	
True	65280	B10	MM			0	
True	255	B11	4h EP#15/1 C6/36 ZK 5.4	37.91	7.40E0	0	E - Extrapolated concentration in standard curve
True	255	B12	22h EP#15/1 C6/36 ZK 5.4	40.07	1.66E0	0	E - Extrapolated concentration in standard curve
True	255	C1	4h EP#15/1 870 cap1 1.4	23.26	1.91E5	0	
True	255	C2	4h EP#15/1 870 cap1 1.5	20.77	1.07E6	0	E - Extrapolated concentration in standard curve
True	255	C3	4h EP#15/1 870 cap1 1.6	20.76	1.08E6	0	E - Extrapolated concentration in standard curve
True	255	C4	4h EP#16/2 870 cap1 2.4	21.82	5.14E5	0	
True	255	C5	4h EP#16/2 870 cap1 2.5	21.12	8.36E5	0	E - Extrapolated concentration in standard curve
True	255	C6	4h EP#16/2 870 cap1 2.6	21.41	6.84E5	0	E - Extrapolated concentration in standard curve
True	255	C7	4h EP#15/3 871 cap0 3.4	21.45	6.67E5	0	E - Extrapolated concentration in standard curve
True	255	C8	4h EP#15/3 871 cap0 3.5	20.64	1.17E6	0	E - Extrapolated concentration in standard curve
True	255	C9	4h EP#15/3 871 cap0 3.6	20.44	1.34E6	0	E - Extrapolated concentration in standard curve
True	255	C10	4h EP#16/4 871 cap0 4.4	22.11	4.22E5	0	
True	255	C11	4h EP#16/4 871 cap0 4.5	21.50	6.43E5	0	E - Extrapolated concentration in standard curve
True	255	C12	4h EP#16/4 871 cap0 4.5	21.97	4.64E5	0	
True	255	D1	8h EP#15/1 870 cap1 1.4	21.46	6.63E5	0	E - Extrapolated concentration in standard curve
True	255	D2	8h EP#15/1 870 cap1 1.5	19.98	1.85E6	0	E - Extrapolated concentration in standard curve
True	255	D3	8h EP#15/1 870 cap1 1.6	20.36	1.42E6	0	E - Extrapolated concentration in standard curve
True	255	D4	8h EP#16/2 870 cap1 2.4	21.31	7.35E5	0	E - Extrapolated concentration in standard curve
True	255	D5	8h EP#16/2 870 cap1 2.5	23.10	2.12E5	0	
True	255	D6	8h EP#16/2 870 cap1 2.6	21.03	8.93E5	0	E - Extrapolated concentration in standard curve
True	255	D7	8h EP#15/3 871 cap0 3.4	20.37	1.40E6	0	E - Extrapolated concentration in standard curve
True	255	D8	8h EP#15/3 871 cap0 3.5	20.60	1.20E6	0	E - Extrapolated concentration in standard curve
True	255	D9	8h EP#15/3 871 cap0 3.6	20.17	1.62E6	0	E - Extrapolated concentration in standard curve
True	255	D10	8h EP#16/4 871 cap0 4.4	21.86	5.02E5	0	
True	255	D11	8h EP#16/4 871 cap0 4.5	21.04	8.87E5	0	E - Extrapolated concentration in standard curve
True	255	D12	8h EP#16/4 871 cap0 4.5	20.75	1.08E6	0	E - Extrapolated concentration in standard curve
True	255	E1	22h EP#15/1 870 cap1 1.4	19.78	2.12E6	0	E - Extrapolated concentration in standard curve
True	255	E2	22h EP#15/1 870 cap1 1.5	19.68	2.26E6	0	E - Extrapolated concentration in standard curve
True	255	E3	22h EP#15/1 870 cap1 1.6	19.10	3.38E6	0	E - Extrapolated concentration in standard curve
True	255	E4	22h EP#16/2 870 cap1 2.4	21.57	6.12E5	0	E - Extrapolated concentration in standard curve
True	255	E5	22h EP#16/2 870 cap1 2.5	19.98	1.84E6	0	E - Extrapolated concentration in standard curve
True	255	E6	22h EP#16/2 870 cap1 2.6	19.64	2.33E6	0	E - Extrapolated concentration in standard curve
True	255	E7	22h EP#15/3 871 cap0 3.4	20.11	1.69E6	0	E - Extrapolated concentration in standard curve
True	255	E8	22h EP#15/3 871 cap0 3.5	19.17	3.22E6	0	E - Extrapolated concentration in standard curve
True	255	E9	22h EP#15/3 871 cap0 3.6	19.86	2.00E6	0	E - Extrapolated concentration in standard curve
True	255	E10	22h EP#16/4 871 cap0 4.4	21.32	7.28E5	0	E - Extrapolated concentration in standard curve
True	255	E11	22h EP#16/4 871 cap0 4.5	20.70	1.12E6	0	E - Extrapolated concentration in standard curve
True	255	E12	22h EP#16/4 871 cap0 4.5	20.24	1.54E6	0	E - Extrapolated concentration in standard curve
True	128	F1	YF-Standard 10-7	21.88	4.93E5	588000	
True	128	F2	YF-Standard 10-8	24.89	6.12E4	58800	
True	128	F3	YF-Standard 10-9	28.21	6.17E3	5880	
True	128	F4	YF-Standard 10-10	31.89	4.80E2	588	
False	128	F5	YF-Standard 10-11			58.8	
True	65280	F6	YF-Standard 10-12			0	
True	128	F7	YF-Standard 10-7	21.48	6.52E5	588000	
True	128	F8	YF-Standard 10-8	24.96	5.86E4	58800	
True	128	F9	YF-Standard 10-9	28.16	6.39E3	5880	
True	128	F10	YF-Standard 10-10	31.43	6.61E2	588	
True	128	F11	YF-Standard 10-11	34.93	5.85E1	58.8	
True	65280	F12	YF-Standard 10-12			0	
True	65280	G1	YF-Standard 10-13			0	
True	65280	G2	H2O			0	
True	65280	G3	YF-Standard 10-13			0	
True	65280	G4	H2O			0	
True	65280	G5	Standard rps7_B 10e1			0	
True	65280	G6	Standard rps7_B 10e0			0	
True	65280	G7	H2O			0	
True	65280	G8	Standard rps7_B 10e1			0	
True	65280	G9	Standard rps7_B 10e0			0	
True	65280	G10	H2O			0	
False	10789024	G11	MM			0	
True	65280	G12	MM			0	
True	65280	H1	Standard rps7_B 10e7			0	
True	255	H2	Standard rps7_B 10e6	40.02	1.72E0	0	E - Extrapolated concentration in standard curve
True	65280	H3	Standard rps7_B 10e5			0	
True	65280	H4	Standard rps7_B 10e4			0	
True	65280	H5	Standard rps7_B 10e3			0	
True	65280	H6	Standard rps7_B 10e2			0	
True	65280	H7	Standard rps7_B 10e7			0	
True	65280	H8	Standard rps7_B 10e6			0	
True	65280	H9	Standard rps7_B 10e5			0	
True	65280	H10	Standard rps7_B 10e4			0	
True	65280	H11	Standard rps7_B 10e3			0	
True	65280	H12	Standard rps7_B 10e2			0	

PRS7 mRNA copies

Experiment: 2024.07.23 wdh cap1 and cap0 probes with YF-Standard and RPS7-Standard Selected Filter: VIC / HEX / Yellow555 (533-580)

Include	Color	Pos	Name	Cp	Concentration	Standard	Status
True	255	A1	4h EP#195/3 870 cap1 3.4	19.54	7.28E6	0	
True	255	A2	4h EP#195/3 870 cap1 3.5	19.30	8.60E6	0	
True	255	A3	4h EP#195/3 870 cap1 3.6	22.12	1.24E6	0	
True	255	A4	4h EP#195/4 871 cap0 4.4	26.68	5.46E4	0	
True	255	A5	4h EP#195/4 871 cap0 4.5	23.72	4.15E5	0	
True	255	A6	4h EP#195/4 871 cap0 4.6	24.00	3.42E5	0	
True	255	A7	8h EP#195/3 870 cap1 3.4	18.26	1.74E7	0	
True	255	A8	8h EP#195/3 870 cap1 3.5	18.19	1.84E7	0	
True	255	A9	8h EP#195/3 870 cap1 3.6	18.95	1.09E7	0	
True	255	A10	8h EP#195/4 871 cap0 4.4	18.20	1.82E7	0	
True	255	A11	8h EP#195/4 871 cap0 4.5	18.00	2.09E7	0	
True	255	A12	8h EP#195/4 871 cap0 4.6	18.16	1.88E7	0	
True	255	B1	22h EP#195/3 870 cap1 3.4	21.24	2.27E6	0	
True	255	B2	22h EP#195/3 870 cap1 3.5	21.34	2.12E6	0	
True	255	B3	22h EP#195/3 870 cap1 3.6	19.00	1.05E7	0	
True	255	B4	22h EP#195/4 871 cap0 4.4	21.24	2.27E6	0	
True	255	B5	22h EP#195/4 871 cap0 4.5	21.35	2.11E6	0	
True	255	B6	22h EP#195/4 871 cap0 4.6	26.45	6.41E4	0	
True	255	B7	4h EP#195/5 C6/36 ZK 5.4	24.85	1.92E5	0	
True	255	B8	22h EP#195/5 C6/36 ZK 5.4	22.87	7.44E5	0	
True	65280	B9	MM			0	
True	65280	B10	MM			0	
True	255	B11	4h EP#15/1 C6/36 ZK 5.4	19.15	9.53E6	0	
True	255	B12	22h EP#15/1 C6/36 ZK 5.4	18.57	1.42E7	0	
True	255	C1	4h EP#15/1 870 cap1 1.4	21.56	1.83E6	0	
True	255	C2	4h EP#15/1 870 cap1 1.5	19.28	8.70E6	0	
True	255	C3	4h EP#15/1 870 cap1 1.6	19.43	7.83E6	0	
True	255	C4	4h EP#16/2 870 cap1 2.4	20.04	5.17E6	0	
True	255	C5	4h EP#16/2 870 cap1 2.5	19.35	8.27E6	0	
True	255	C6	4h EP#16/2 870 cap1 2.6	19.63	6.83E6	0	
True	255	C7	4h EP#15/3 871 cap0 3.4	20.19	4.65E6	0	
True	255	C8	4h EP#15/3 871 cap0 3.5	19.31	8.51E6	0	
True	255	C9	4h EP#15/3 871 cap0 3.6	18.85	1.17E7	0	
True	255	C10	4h EP#16/4 871 cap0 4.4	20.34	4.20E6	0	
True	255	C11	4h EP#16/4 871 cap0 4.5	19.61	6.95E6	0	
True	255	C12	4h EP#16/4 871 cap0 4.5	20.41	4.00E6	0	
True	255	D1	8h EP#15/1 870 cap1 1.4	19.87	5.81E6	0	
True	255	D2	8h EP#15/1 870 cap1 1.5	18.66	1.33E7	0	
True	255	D3	8h EP#15/1 870 cap1 1.6	19.06	1.01E7	0	
True	255	D4	8h EP#16/2 870 cap1 2.4	19.12	9.71E6	0	
True	255	D5	8h EP#16/2 870 cap1 2.5	21.37	2.07E6	0	
True	255	D6	8h EP#16/2 870 cap1 2.6	19.36	8.24E6	0	
True	255	D7	8h EP#15/3 871 cap0 3.4	18.73	1.27E7	0	
True	255	D8	8h EP#15/3 871 cap0 3.5	18.87	1.15E7	0	
True	255	D9	8h EP#15/3 871 cap0 3.6	19.23	8.98E6	0	
True	255	D10	8h EP#16/4 871 cap0 4.4	20.17	4.71E6	0	
True	255	D11	8h EP#16/4 871 cap0 4.5	19.07	1.00E7	0	
True	255	D12	8h EP#16/4 871 cap0 4.5	18.89	1.14E7	0	
True	255	E1	22h EP#15/1 870 cap1 1.4	18.23	1.79E7	0	
True	255	E2	22h EP#15/1 870 cap1 1.5	18.31	1.69E7	0	
True	255	E3	22h EP#15/1 870 cap1 1.6	17.58	2.78E7	0	
True	255	E4	22h EP#16/2 870 cap1 2.4	19.15	9.53E6	0	
True	255	E5	22h EP#16/2 870 cap1 2.5	17.80	2.40E7	0	
True	255	E6	22h EP#16/2 870 cap1 2.6	17.50	2.95E7	0	
True	255	E7	22h EP#15/3 871 cap0 3.4	18.11	1.94E7	0	
True	255	E8	22h EP#15/3 871 cap0 3.5	17.46	3.03E7	0	
True	255	E9	22h EP#15/3 871 cap0 3.6	17.52	2.89E7	0	
True	255	E10	22h EP#16/4 871 cap0 4.4	18.43	1.56E7	0	
True	255	E11	22h EP#16/4 871 cap0 4.5	18.16	1.88E7	0	
True	255	E12	22h EP#16/4 871 cap0 4.5	18.37	1.62E7	0	
True	65280	F1	YF-Standard 10-7			0	
True	65280	F2	YF-Standard 10-8			0	
True	65280	F3	YF-Standard 10-9			0	
True	65280	F4	YF-Standard 10-10			0	
True	65280	F5	YF-Standard 10-11			0	
True	65280	F6	YF-Standard 10-12			0	
True	65280	F7	YF-Standard 10-7			0	
True	65280	F8	YF-Standard 10-8			0	
True	65280	F9	YF-Standard 10-9			0	
True	65280	F10	YF-Standard 10-10			0	
True	65280	F11	YF-Standard 10-11			0	
True	65280	F12	YF-Standard 10-12			0	
True	65280	G1	YF-Standard 10-13			0	
True	65280	G2	H2O			0	
True	65280	G3	YF-Standard 10-13			0	
True	65280	G4	H2O			0	
True	128	G5	Standard rps7_B 10e1	37.27	3.84E1	40	
True	65280	G6	Standard rps7_B 10e0			0	
True	65280	G7	H2O			0	
True	128	G8	Standard rps7_B 10e1	36.27	7.63E1	40	
True	255	G9	Standard rps7_B 10e0	40.65	3.80E0	0	E - Extrapolated concentration in standard curve
True	65280	G10	H2O			0	
True	65280	G11	MM			0	
True	65280	G12	MM			0	
True	128	H1	Standard rps7_B 10e7	16.97	4.24E7	40000000	
True	128	H2	Standard rps7_B 10e6	20.32	4.25E6	4000000	
True	128	H3	Standard rps7_B 10e5	23.73	4.12E5	400000	
True	128	H4	Standard rps7_B 10e4	27.06	4.21E4	40000	
True	128	H5	Standard rps7_B 10e3	30.50	3.98E3	4000	
True	128	H6	Standard rps7_B 10e2	34.15	3.26E2	400	
True	128	H7	Standard rps7_B 10e7	16.99	4.18E7	40000000	
True	128	H8	Standard rps7_B 10e6	20.26	4.43E6	4000000	
True	128	H9	Standard rps7_B 10e5	24.05	3.30E5	400000	
True	128	H10	Standard rps7_B 10e4	27.11	4.07E4	40000	
True	128	H11	Standard rps7_B 10e3	30.79	3.27E3	4000	
True	128	H12	Standard rps7_B 10e2	34.39	2.78E2	400	

Fig. 5A

Cap1

Genome copies per 3µl RNA

Carcass															
days	1	2	3	4	5	6	7	8	9	10	11	12	13	14	15
0	22600.00	10600.00	11700.00	30600.00	28300.00	4030.00	26200.00	29500.00	28200.00	14900.00	21800.00	18700.00	20300.00	22200.00	31000.00
3	8230.00	1720.00	2740.00	13500.00	2.06	591.00	3300.00	0	206.00	13100.00	382.00	254.00	681.00	2210.00	440.00
5	5710.00	13300.00	4510.00	0	2440.00	21800.00	25100.00	3480.00	1170.00	17300.00	9630.00	222.00	73600.00	60000.00	19800.00
7	29400.00	13200.00	37500.00	69100.00	55300.00	1440.00	908.00	0	475000.00	44500.00	7540.00	21500.00	26600.00	24900.00	7720.00
10	2810.00	73900.00	38400.00	92500.00	1960.00	89900.00	89300.00	337.00	106000.00	1650.00	175000.00	176000.00	59200.00	70800.00	38700.00
14	3.23E+04	5.55E+04	1.28E+04	6.20E+04	0	2.76E+04	6.67E+04	1.27E+04	5.42E+03	3.58E+04	2.04E+05	1.96E+04	1.01E+05	7.44E+04	1.28E+05
21	1.74E+04	2.39E+04	1.09E+05	9.89E+04	7.47E+04	7.68E+03	4.16E+04	3.06E+05	1.28E+05	4.73E+04	9.91E+03	1.03E+05	8.95E+04	2.32E+03	0

Carcass															
days	16	17	18	19	20	21	22	23	24	25	26	27	28	29	30
0	24100.00	19300.00	6680.00	20200.00	7300.00	25700.00	8040.00	5650.00	7710.00	17600.00	23800.00	14400.00	28800.00	22900.00	7700.00
3	18900.00	589.00	28800.00	2470.00	10700.00	408.00	16300.00	18300.00	8170.00	1030.00	1200.00	12800.00	326.00	209.00	9450.00
5	696.00	25400.00	2100.00	686.00	1970.00	38500.00	9780.00	16600.00	11000.00	31600.00	40200.00	2060.00	6100.00	54800.00	12900.00
7	1710.00	4990.00	5150.00	17400.00	67700.00	6650.00	69300.00	5200.00	80800.00	21200.00	268000.00	7870.00	36300.00	2.82	56500.00
10	23300.00	88100.00	82100.00	30700.00	68200.00	2670.00	94800.00	10400.00	57100.00	236000.00	14200.00	94100.00	21300.00	5390.00	43000.00
14	8.95E+04	2.96E+04	1.51E+05	5.95E+04	6.81E+04	9.40E+04	1.89E+05	2.74E+04	3.92E+03	1.56E+05	7.06E+04	9.61E+04	4.99E+04	6.42E+04	6.53E+04
21	9.18E+04	3.82E+03	4.50E+04	9.01E+04	1.64E+05	8.07E+04	4.40E+04	1.27E+05	3.59E+04	2.63E+04	2.11E+04	2.64E+04	7.25E+04	1.45E+05	9.00E+04

Carcass														
days	31	32	33	34	35	36	37	38	39	40	41	42	43	44
0	1390.00	4450.00	9170.00	7.24E+04	2.42E+04	3.31E+04	5.02E+04	8.09E+04	8.12E+04	2.27E+04	6.23E+04	2.85E+04	8.94E+04	5.28E+04
3	251.00	1570.00	179.00	29000.00	275.00	2.99E+02	5.79E+03	2.03E+03	9.48E+02	6.43E+03	1.63E+04	1.28E+03	7.27E+03	1.23E+03
5	8960.00	618.00	16400.00	1770.00	9.61E+03	1.89E+04	2.43E+04	1.73E+04	7.76E+03	3.40E+04	3.94E+04	2.52E+03	9.71E+02	6.93E+01
7	33400.00	3990.00	105000.00	3350.00	81900.00	24300.00	8.63E+04	1.04E+03	2.92E+04	6.45E+04	2.01E+04	3.54E+04	5.15E+04	4.11E+03
10	40100.00	91300.00	90300.00	64500.00	0	115000.00	8300.00	17200.00	8.03E+04	1.33E+04	1.06E+05	1.71E+04	1.39E+04	1.76E+04
14	8.84E+04	9.00E+04	1.17E+05	4.63E+04	3.29E+04	1.02E+05	7.32E+03	5.42E+04	9.17E+02	8.20E+04	1.24E+01	2.52E+04	5.15E+02	6.72E+03
21	7.43E+04	1.16E+05	8.21E+04	1.61E+02	1.42E+05	1.66E+05	1.49E+05	6.85E+04	1.43E+04	1.08E+05	4.03E+04	2.59E+05	1.96E+05	9.12E+04

Carcass														
days	45	46	47	48	49	50	51	52	53	54	55	56	57	58
0	5.24E+03	7.53E+04	4.34E+04	5.80E+04	4.36E+04	8.64E+04	2.44E+04	3.36E+04	2.47E+04					
3	1.17E+02	2.42E+03	5.79E+02	6.83E+03	3.95E+02	1.30E+04	1.58E+03	2.98E+03	8.33E+03	1.24E+03				
5	6.31E+03	0	4.26E+03	2.88E+04	1.03E+04	1.33E+04	0	3.05E+02	6.01E+03					
7	1.65E+05	1.81E+04	1.03E+05	4.76E+04	1.35E+04	5.97E+03	7.95E+03	0	1.46E+04	5.64E+04	4.65E+04			
10	2.72E+04	1.24E+04	1.64E+04	1.62E+04	6.00E+02	8.30E+04	0	2.01E+04	4.83E+04	1.91E+04	1.97E+04	4.45E+04	8.23E+04	
14	1.08E+04													
21	9.71E+04													

Genome copies per Moskito

Carcass															
days	1	2	3	4	5	6	7	8	9	10	11	12	13	14	15
0	9.69E+05	4.54E+05	5.01E+05	1.31E+06	1.21E+06	1.73E+05	1.12E+06	1.26E+06	1.21E+06	6.39E+05	9.34E+05	8.01E+05	8.70E+05	9.51E+05	1.33E+06
3	3.53E+05	7.37E+04	1.17E+05	5.79E+05	8.83E+01	2.53E+04	1.41E+05	0.00E+00	8.83E+03	5.61E+05	1.64E+04	1.09E+04	2.92E+04	9.47E+04	1.89E+04
5	2.45E+05	5.70E+05	1.93E+05	0.00E+00	1.05E+05	9.34E+05	1.08E+06	1.49E+05	5.01E+04	7.41E+05	4.13E+05	9.51E+03	3.15E+06	2.57E+06	8.49E+05
7	1.26E+06	5.66E+05	1.61E+06	2.96E+06	2.37E+06	6.17E+04	3.89E+04	0.00E+00	2.04E+07	1.91E+06	3.23E+05	9.21E+05	1.14E+06	1.07E+06	3.31E+05
10	1.20E+05	3.17E+06	1.65E+06	3.96E+06	8.40E+04	3.85E+06	3.83E+06	1.44E+04	4.54E+06	7.07E+04	7.50E+06	7.54E+06	2.54E+06	3.03E+06	1.66E+06
14	1.38E+06	2.38E+06	5.49E+05	2.66E+06	0.00E+00	1.18E+06	2.86E+06	5.44E+05	2.32E+05	1.53E+06	8.74E+06	8.40E+05	4.33E+06	3.19E+06	5.49E+06
21	7.46E+05	1.02E+06	4.67E+06	4.24E+06	3.20E+06	3.29E+05	1.78E+06	1.31E+07	5.49E+06	2.03E+06	4.25E+05	4.41E+06	3.84E+06	9.94E+04	0.00E+00

Carcass															
days	16	17	18	19	20	21	22	23	24	25	26	27	28	29	30
0	1.03E+06	8.27E+05	2.86E+05	8.66E+05	3.13E+05	1.10E+06	3.45E+05	2.42E+05	3.30E+05	7.54E+05	1.02E+06	6.17E+05	1.23E+06	9.81E+05	3.30E+05
3	8.10E+05	2.52E+04	1.23E+06	1.06E+05	4.59E+05	1.75E+04	6.99E+05	7.84E+05	3.50E+05	4.41E+04	5.14E+04	5.49E+05	1.40E+04	8.96E+03	4.05E+05
5	2.98E+04	1.09E+06	9.00E+04	2.94E+04	8.44E+04	1.65E+06	4.19E+05	7.11E+05	4.71E+05	1.35E+06	1.72E+06	8.83E+04	2.61E+05	2.35E+06	5.53E+05
7	7.33E+04	2.14E+05	2.21E+05	7.46E+05	2.90E+06	2.85E+05	2.97E+06	2.23E+05	3.46E+06	9.09E+05	1.15E+07	3.37E+05	1.56E+06	1.21E+02	2.42E+06
10	9.99E+05	3.78E+06	3.52E+06	1.32E+06	2.92E+06	1.14E+05	4.06E+06	4.46E+05	2.45E+06	1.01E+07	6.09E+05	4.03E+06	9.13E+05	2.31E+05	1.84E+06
14	3.84E+06	1.27E+06	6.47E+06	2.55E+06	2.92E+06	4.03E+06	8.10E+06	1.17E+06	1.68E+05	6.69E+06	3.03E+06	4.12E+06	2.14E+06	2.75E+06	2.80E+06
21	3.93E+06	1.64E+05	1.93E+06	3.86E+06	7.03E+06	3.46E+06	1.89E+06	5.44E+06	1.54E+06	1.13E+06	9.04E+05	1.13E+06	3.11E+06	6.21E+06	3.86E+06

Carcass														
days	31	32	33	34	35	36	37	38	39	40	41	42	43	44
0	5.96E+04	1.91E+05	3.93E+05	3.10E+06	1.04E+06	1.42E+06	2.15E+06	3.47E+06	3.48E+06	9.73E+05	2.67E+06	1.22E+06	3.83E+06	2.26E+06
3	1.08E+04	6.73E+04	7.67E+03	1.24E+06	1.18E+04	1.28E+04	2.48E+05	8.70E+04	4.06E+04	2.76E+05	6.99E+05	5.49E+04	3.12E+05	5.27E+04
5	3.84E+05	2.65E+04	7.03E+05	7.59E+04	4.12E+05	8.10E+05	1.04E+06	7.41E+05	3.33E+05	1.46E+06	1.69E+06	1.08E+05	4.16E+04	2.97E+03
7	1.43E+06	1.71E+05	4.50E+06	1.44E+05	3.51E+06	1.04E+06	3.70E+06	4.46E+04	1.25E+06	2.76E+06	8.61E+05	1.52E+06	2.21E+06	1.76E+05
10	1.72E+06	3.91E+06	3.87E+06	2.76E+06	0.00E+00	4.93E+06	3.56E+05	7.37E+05	3.44E+06	5.70E+05	4.54E+06	7.33E+05	5.96E+05	7.54E+05
14	3.79E+06	3.86E+06	5.01E+06	1.98E+06	1.41E+06	4.37E+06	3.14E+05	2.32E+06	3.93E+04	3.51E+06	5.31E+02	1.08E+06	2.21E+04	2.88E+05
21	3.18E+06	4.97E+06	3.52E+06	6.90E+03	6.09E+06	7.11E+06	6.39E+06	2.94E+06	6.13E+05	4.63E+06	1.73E+06	1.11E+07	8.40E+06	3.91E+06

Carcass														
days	45	46	47	48	49	50	51	52	53	54	55	56	57	58
0	2.25E+05	3.23E+06	1.86E+06	2.49E+06	1.87E+06	3.70E+06	1.05E+06	1.44E+06	1.06E+06					
3	5.01E+03	1.04E+05	2.48E+04	2.93E+05	1.69E+04	5.57E+05	6.77E+04	1.28E+05	3.57E+05	5.31E+04				
5	2.70E+05	0.00E+00	1.83E+05	1.23E+06	4.41E+05	5.70E+05	0.00E+00	1.31E+04	2.58E+05					
7	7.07E+06	7.76E+05	4.41E+06	2.04E+06	5.79E+05	2.56E+05	3.41E+05	0.00E+00	6.26E+05	2.42E+06	1.99E+06			
10	1.17E+06	5.31E+05	7.03E+05	6.94E+05	2.57E+04	3.56E+06	0.00E+00	8.61E+05	2.07E+06	8.19E+05	8.44E+05	1.91E+06	3.53E+06	
14	4.63E+05													
21	4.16E+06													

Cap0

Genome copies per 3µl RNA

Carcass																			
days	1	2	3	4	5	6	7	8	9	10	11	12	13	14	15	16	17	18	19
0	1.84E+04	2.23E+04	2.70E+04	7.81E+04	1.51E+04	1.48E+04	1.54E+04	1.92E+04	1.01E+04	2.07E+04	1.67E+04	1.41E+04	9.06E+03	1.65E+04	2.25E+04	2.09E+03	1.55E+04	9.90E+03	2.12E+04
3	1.05E+01	0	0	0	0	1.15E+02	0	0	0	0	8.74E+00	0	0	0	0	1.02E+01	4.69E+01	0	8.39E+01
5	1.78E+01	0	0	0	4.40E+00	0	1.77E+02	0	0	0	3.17E+00	0	0	0	0	0	0	0	0
7	0	0	0	0	0	0	5.88E+02	0	0	0	7.53E+01	0	4.38E+01	0	0	0	0	0	1.28E+02
10	0	0	0	0	0	0	0	0	0	0	0	0	0	0	8.12E+01	3.19E+00	0	0	5.42E+00
14	0	0	0	0	0	0	0	0	0	5.87E+00	0	0	0	0	0	0	0	0	0
21	0	0	0	8.38E+00	0	0	0	0	0	0	0	0	4.37E+00	0	5.57E+00	0	0	0	1.65E+01

Carcass																		
days	20	21	22	23	24	25	26	27	28	29	30	31	32	33	34	35	36	37
0	8.98E+03	1.32E+04	5.18E+03	1.30E+04	1.94E+04	1.84E+04	8.72E+03	7.72E+03	9.95E+03	2.83E+04	1.14E+04	1.98E+03	7.22E+02	3.89E+03				
3	0	5.16E+00	3.64E+01	0	2.82E+02	0	0	0	0	0	0	1.55E+00	0	3.88E+03				
5	2.20E+01	1.10E+01	0	0	0	0	0	0	0	0	0	0						
7	1.02E+01	0	0	1.74E+01	0	0	0	0	0	0	0							
10	0	0	0	1.46E+02	0	1.05E+01	0	1.40E+03	0	0	2.97E+00							
14	1.68E+02	0	2.69E+00	1.00E+04	0	5.84E+01	0	0	7.98E+04	0	0	1.48E+00						
21	1.57E+03	4.59E+01	0	0	0	0	0	0	2.51E+02	0	0	0	0	0	0	2.11E+02	0	2.14E+00

Genome copies per Moskito

Carcass																			
days	1	2	3	4	5	6	7	8	9	10	11	12	13	14	15	16	17	18	19
0	7.89E+05	9.56E+05	1.16E+06	3.35E+06	6.47E+05	6.34E+05	6.60E+05	8.23E+05	4.33E+05	8.87E+05	7.16E+05	6.04E+05	3.88E+05	7.07E+05	9.64E+05	8.96E+04	6.64E+05	4.24E+05	9.09E+05
3	4.50E+02	0	0	0	0	4.93E+03	0	0	0	0	3.75E+02	0	0	0	0	4.37E+02	2.01E+03	0	3.60E+03
5	7.63E+02	0	0	0	1.89E+02	0	7.59E+03	0	0	0	1.36E+02	0	0	0	0	0	0	0	0
7	0	0	0	0	0	0	2.52E+04	0	0	0	3.23E+03	0	1.88E+03	0	0	0	0	0	5.49E+03
10	0	0	0	0	0	0	0	0	0	0	0	0	0	0	3.48E+03	1.37E+02	0	0	2.32E+02
14	0	0	0	0	0	0	0	0	0	2.52E+02	0	0	0	0	0	0	0	0	0
21	0	0	0	3.59E+02	0	0	0	0	0	0	0	0	1.87E+02	0	2.39E+02	0	0	0	7.07E+02

Carcass																		
days	20	21	22	23	24	25	26	27	28	29	30	31	32	33	34	35	36	37
0	3.85E+05	5.66E+05	2.22E+05	5.57E+05	8.31E+05	7.89E+05	3.74E+05	3.31E+05	4.26E+05	1.21E+06	4.89E+05	8.49E+04	3.09E+04	1.67E+05				
3	0	2.21E+02	1.56E+03	0	1.21E+04	0	0	0	0	0	0	6.64E+01	0	1.66E+05				
5	9.43E+02	4.71E+02	0	0	0	0	0	0	0	0	0	0						
7	4.37E+02	0	0	7.46E+02	0	0	0	0	0	0	0							
10	0	0	0	6.26E+03	0	4.50E+02	0	6.00E+04	0.00E+00	0	1.27E+02							
14	7.20E+03	0	1.15E+02	4.29E+05	0	2.50E+03	0	0	3.42E+06	0	0.00E+00	6.34E+01						
21	6.73E+04	1.97E+03	0	0	0	0	0	0	1.08E+04	0	0	0	0	0	0	9.04E+03	0	9.17E+01

Fig. 5B

Cap1

Genome copies per 3µl RNA

legs + wings																													
days	1	2	3	4	5	6	7	8	9	10	11	12	13	14	15	16	17	18	19	20	21	22	23	24	25	26	27	28	29
0	0	0	0	0	0	0	0	0	0	0	0	0	0	0	3.36E+01	0	0	0	0	0	7.01E+00	2.57E+00	0	1.74E+00	0	2.96E+00	0	2.89E+00	0
3	0	0	0	0	0	0	0	3.37E+00	0	0	0	0	3.99E+00	3.02E+00	3.91E+00	1.48E+03	0	0	0	2.24E+00	0	2.07E+00	2.52E+03	0	0	3.29E+00	0	3.50E+00	0
5	0	0	0	3.65E+00	2.60E+00	0	0	3.43E+00	0	0	3.58E+00	0	2.73E+00	0	1.77E+02	0	0	0	4.22E+00	0	3.12E+00	2.58E+00	1.82E+01	0	0	0	0	0	1.24E+01
7	1.07E+02	0	1.59E+02	1.50E+03	1.73E+02	0	0	0	5.71E+03	1.06E+02	0	0	5.91E+02	4.50E+01	2.97E+00	0	0	0	0	3.63E+02	0	7.30E+01	3.21E+00	6.15E+02	2.06E+02	4.08E+03	1.06E+02	1.96E+02	1.08E+01
10	1.64E+01	3.43E+03	1.10E+03	2.20E+02	0	2.56E+03	1.02E+03	2.62E+00	3.04E+03	0	2.00E+03	3.04E+03	2.23E+03	4.16E+03	1.80E+03	5.25E+02	5.80E+02	1.46E+03	2.39E+02	7.99E+02	1.80E+00	2.77E+03	0	2.26E+03	6.58E+03	1.42E+03	3.50E+03	7.79E+02	2.15E+01
14	1.53E+03	2.47E+03	7.98E+02	8.04E+03	0	1.91E+03	1.06E+04	0	0	3.03E+03	9.90E+03	4.32E+03	1.13E+04	2.62E+03	4.48E+03	6.96E+03	3.00E+03	3.70E+03	3.01E+03	1.31E+03	2.49E+03	1.23E+04	2.71E+03	0	3.41E+03	2.14E+03	2.82E+03	4.42E+03	3.47E+03
21	8.41E+03	2.06E+03	3.44E+04	6.46E+03	3.56E+03	0	4.40E+03	1.38E+04	1.07E+04	9.87E+03	0	4.61E+03	1.68E+04	0	0	1.16E+04	0	7.39E+03	4.20E+03	2.21E+04	1.38E+04	1.37E+04	1.18E+04	5.84E+03	1.54E+03	0	7.31E+03	4.84E+03	9.96E+03

legs + wings																													
days	30	31	32	33	34	35	36	37	38	39	40	41	42	43	44	45	46	47	48	49	50	51	52	53	54	55	56	57	58
0	0	0	0	0																									
3	2.90E+00	4.08E+00	4.85E+02	2.70E+00	0	0	0	0	0	0	0	0	0	0	0	0	0	0	0	0	0	0	0	0	0				
5	0	0	0	0	5.67E+00	0	2.34E+01	0	0	0	0	0	2.22E+01	0	0	5.14E+01	0	0	4.25E+02	0	0	0	1.87E+02	1.22E+00					
7	1.52E+03	8.73E+01	3.43E+00	1.02E+02	0	4.10E+02	4.80E+00	0	7.01E+02	0	3.57E+02	7.72E+02	1.99E+03	9.74E+02	2.18E+02	0	7.49E+02	1.98E+02	7.28E+02	0	0	0	0	7.95E+02	0	5.05E+02			
10	6.31E+02	0	5.45E+03	2.55E+03	7.29E+02	0	3.10E+03	5.20E+00	1.58E+03	9.47E+03	4.21E+03	1.79E+04	5.05E+02	0	7.37E+02	1.10E+03	2.03E+03	1.36E+03	3.28E+02	0	2.22E+03	0	4.11E+03	9.71E+03	1.44E+03	1.50E+03	5.20E+03	4.09E+03	
14	2.10E+04	3.42E+03	5.15E+03	1.49E+04	3.67E+03	3.58E+03	2.19E+04	0	3.44E+03	0	3.31E+03	0	4.14E+03	0	7.48E+02	8.65E+02													
21	1.98E+04	3.07E+03	6.50E+03	4.58E+03	3.98E+01	1.09E+04	1.47E+04	1.19E+04	4.46E+03	0	5.65E+04	1.26E+04	3.00E+04	7.60E+03	2.55E+04	2.53E+01													

Genome copies per Moskito

legs + wings																													
days	1	2	3	4	5	6	7	8	9	10	11	12	13	14	15	16	17	18	19	20	21	22	23	24	25	26	27	28	29
0	0	0	0	0	0	0	0	0	0	0	0	0	0	0	1.44E+03	0	0	0	0	0	3.00E+02	1.10E+02	0	7.46E+01	0	1.27E+02	0	1.24E+02	0
3	0	0	0	0	0	0	0	1.44E+02	0	0	0	0	1.71E+02	1.29E+02	1.68E+02	6.34E+04	0	0	0	9.60E+01	0	8.87E+01	1.08E+05	0	0	1.41E+02	0	1.50E+02	0
5	0	0	0	1.56E+02	1.11E+02	0	0	1.47E+02	0	0	1.53E+02	0	1.17E+02	0	7.59E+03	0	0	0	1.81E+02	0	1.34E+02	1.11E+02	7.80E+02	0	0	0	0	0	5.31E+02
7	4.59E+03	0	6.81E+03	6.43E+04	7.41E+03	0	0	0	2.45E+05	4.54E+03	0	0	2.53E+04	1.93E+03	1.27E+02	0	0	0	0	1.56E+04	0	3.13E+03	1.38E+02	2.64E+04	8.83E+03	1.75E+05	4.54E+03	8.40E+03	4.63E+02
10	7.03E+02	1.47E+05	4.71E+04	9.43E+03	0	1.10E+05	4.37E+04	1.12E+02	1.30E+05	0	8.57E+04	1.30E+05	9.56E+04	1.78E+05	7.71E+04	2.25E+04	2.49E+04	6.26E+04	1.02E+04	3.42E+04	7.71E+01	1.19E+05	0	9.69E+04	2.82E+05	6.09E+04	1.50E+05	3.34E+04	9.21E+02
14	6.56E+04	1.06E+05	3.42E+04	3.45E+05	0	8.19E+04	4.54E+05	0	0	1.30E+05	4.24E+05	1.85E+05	4.84E+05	1.12E+05	1.92E+05	2.98E+05	1.29E+05	1.59E+05	1.29E+05	5.61E+04	1.07E+05	5.27E+05	1.16E+05	0	1.46E+05	9.17E+04	1.21E+05	1.89E+05	1.49E+05
21	3.60E+05	8.83E+04	1.47E+06	2.77E+05	1.53E+05	0	1.89E+05	5.91E+05	4.59E+05	4.23E+05	0	1.98E+05	7.20E+05	0	0	4.97E+05	0	3.17E+05	1.80E+05	9.47E+05	5.91E+05	5.87E+05	5.06E+05	2.50E+05	6.60E+04	0	3.13E+05	2.07E+05	4.27E+05

legs + wings																													
days	30	31	32	33	34	35	36	37	38	39	40	41	42	43	44	45	46	47	48	49	50	51	52	53	54	55	56	57	58
0	0	0	0	0																									
3	1.24E+02	1.75E+02	2.08E+04	1.16E+02	0	0	0	0	0	0	0	0	0	0	0	0	0	0	0	0	0	0	0	0	0				
5	0	0	0	0	2.43E+02	0	1.00E+03	0	0	0	0	0	9.51E+02	0	0	2.20E+03	0	0	1.82E+04	0	0	0	8.01E+03	5.23E+01					
7	6.51E+04	3.74E+03	1.47E+02	4.37E+03	0	1.76E+04	2.06E+02	0	3.00E+04	0	1.53E+04	3.31E+04	8.53E+04	4.17E+04	9.34E+03	0	3.21E+04	8.49E+03	3.12E+04	0	0	0	0	3.41E+04	0	2.16E+04			
10	2.70E+04	0	2.34E+05	1.09E+05	3.12E+04	0	1.33E+05	2.23E+02	6.77E+04	4.06E+05	1.80E+05	7.67E+05	2.16E+04	0	3.16E+04	4.71E+04	8.70E+04	5.83E+04	1.41E+04	0	9.51E+04	0	1.76E+05	4.16E+05	6.17E+04	6.43E+04	2.23E+05	1.75E+05	
14	9.00E+05	1.47E+05	2.21E+05	6.39E+05	1.57E+05	1.53E+05	9.39E+05	0	1.47E+05	0	1.42E+05	0	1.77E+05	0	3.21E+04	3.71E+04													
21	8.49E+05	1.32E+05	2.79E+05	1.96E+05	1.71E+03	4.67E+05	6.30E+05	5.10E+05	1.91E+05	0	2.42E+06	5.40E+05	1.29E+06	3.26E+05	1.09E+06	1.08E+03													

Cap0

Genome copies per 3µl RNA

legs + wings																																					
days	1	2	3	4	5	6	7	8	9	10	11	12	13	14	15	16	17	18	19	20	21	22	23	24	25	26	27	28	29	30	31	32	33	34	35	36	37
0	0	0	0	0	0	0	0	0	0	0	0	0	0	0	0	0	0	0	0	0	0	0	0		0	0	0	0	0	0	0	0	0				
3	0	0	0	0	0	0	0	0	0	0	0	0	0	0	0	0	0	0	0	0	0	0	0	2.84E+00	0	0	0	0	1.88E+00	0	0	0	0				
5	0	0	0	0	0	0	0	0	0	0	0	0	0	0	0	0	0	0	0	0	0	0	0	0	0	0	0	0	0	0	0						
7	0	0	0	0	6.23E+00	0	0	0	0	0	0	0	0	0	0	0	0	0	0	0	0	0	0	0	0	0	0	0	0	0							
10	0	0	0	0	0	0	0	0	0	0	0	0	0	0	0	0	0	0	0	0	0	0	0	0	0	0	3.94E+02	0	0	0							
14	0	0	0	0	0	0	0	0	0	0	0	0	0	0	0	0	0	0	0	0	0	0	2.34E+02	0	0	0	0	1.72E+03	0	0	0						
21	0	0	0	1.78E+01	0	0	0	0	0	0	0	0	0	0	0	0	0	0	0	0	0	0	0	0	0	0	0	0	0	0	0	0	0	0	0	0	0

legs + wings																																					
days	1	2	3	4	5	6	7	8	9	10	11	12	13	14	15	16	17	18	19	20	21	22	23	24	25	26	27	28	29	30	31	32	33	34	35	36	37
0	0	0	0	0	0	0	0	0	0	0	0	0	0	0	0	0	0	0	0	0	0	0	0	0	0	0	0	0	0	0	0	0	0				
3	0	0	0	0	0	0	0	0	0	0	0	0	0	0	0	0	0	0	0	0	0	0	0	1.22E+02	0	0	0	0	8.06E+01	0	0	0	0				
5	0	0	0	0	0	0	0	0	0	0	0	0	0	0	0	0	0	0	0	0	0	0	0	0	0	0	0	0	0	0	0						
7	0	0	0	0	2.67E+02	0	0	0	0	0	0	0	0	0	0	0	0	0	0	0	0	0	0	0	0	0	0	0	0	0							
10	0	0	0	0	0	0	0	0	0	0	0	0	0	0	0	0	0	0	0	0	0	0	0	0	0	0	1.69E+04	0	0	0							
14	0	0	0	0	0	0	0	0	0	0	0	0	0	0	0	0	0	0	0	0	0	0	1.00E+04	0	0	0	0	7.37E+04	0	0	0						
21	0	0	0	7.63E+02	0	0	0	0	0	0	0	0	0	0	0	0	0	0	0	0	0	0	0	0	0	0	0	0	0	0	0	0	0	0	0	0	0

Fig. 6A

Cap1

Genome copies per 3µl RNA

midgut																								
days	1	2	3	4	5	6	7	8	9	10	11	12	13	14	15	16	17	18	19	20	21	22	23	24
0	3.08E+03	1.32E+04	1.14E+04	6.25E+03	1.26E+04																			
3	1.55E+03	8.52E+00	8.99E+03	3.67E+03	9.04E+02	2.04E+03	0	1.71E+03	2.62E+03	2.78E+03	4.05E+02	5.78E+02	1.19E+04	3.28E+03	6.32E+03	7.15E+03	1.80E+03							
7	1.70E+03	3.45E+03	2.44E+04	1.91E+03	4.77E+03	3.43E+04	4.71E+03	1.87E+04	3.39E+04	1.99E+04	1.09E+04	8.69E+03	1.61E+04	6.81E+03	7.84E+04	1.74E+03	6.81E+02	2.63E+04	0	4.24E+04	5.86E+03	8.57E+03	2.18E+02	7.87E+03
14	1.53E+03	3.30E+03	2.11E+03	1.97E+03	4.13E+03	1.47E+03	4.48E+03	4.64E+03	4.47E+03	9.12E+03	9.46E+01	4.53E+03	1.05E+03	4.86E+02	4.87E+03	4.23E+03	9.18E+03	4.77E+03	8.75E+03	4.78E+04	2.30E+03	9.08E+02	1.09E+03	

Genome copies per Moskito

midgut																								
days	1	2	3	4	5	6	7	8	9	10	11	12	13	14	15	16	17	18	19	20	21	22	23	24
0	1.32E+05	5.66E+05	4.89E+05	2.68E+05	5.40E+05																			
3	6.64E+04	3.65E+02	3.85E+05	1.57E+05	3.87E+04	8.74E+04	0	7.33E+04	1.12E+05	1.19E+05	1.74E+04	2.48E+04	5.10E+05	1.41E+05	2.71E+05	3.06E+05	7.71E+04							
7	7.29E+04	1.48E+05	1.05E+06	8.19E+04	2.04E+05	1.47E+06	2.02E+05	8.01E+05	1.45E+06	8.53E+05	4.67E+05	3.72E+05	6.90E+05	2.92E+05	3.36E+06	7.46E+04	2.92E+04	1.13E+06	0	1.82E+06	2.51E+05	3.67E+05	9.34E+03	3.37E+05
14	6.56E+04	1.41E+05	9.04E+04	8.44E+04	1.77E+05	6.30E+04	1.92E+05	1.99E+05	1.92E+05	3.91E+05	4.05E+03	1.94E+05	4.50E+04	2.08E+04	2.09E+05	1.81E+05	3.93E+05	2.04E+05	3.75E+05	2.05E+06	9.86E+04	3.89E+04	4.67E+04	

Cap0

Genome copies per 3µl RNA

midgut																														
days	1	2	3	4	5	6	7	8	9	10	11	12	13	14	15	16	17	18	19	20	21	22	23	24	25	26	27	28	29	30
0	1.67E+04	1.48E+04	1.62E+03	8.13E+03	7.41E+03																									
3	0	1.04E+02	1.27E+01	0	0	1.68E+02	0	0	8.83E+00	0	8.03E+01	2.17E+02	3.22E+01	4.39E+02	0	0	0	8.75E+00	6.99E+01	1.79E+02	0	0	2.38E+02	3.12E+00	1.79E+01					
7	0	2.11E+00	6.24E+00	9.90E+01	0	0	9.87E+01	0	0	3.11E+01	0	2.34E+01	1.31E+01	0	1.14E+02	2.27E+03	3.05E+03	0	6.69E+03	1.09E+01	0	2.25E+03	1.47E+03	0	4.39E+03					
14	9.45E+02	8.60E+00	3.97E+03	0	1.01E+01	0	2.93E+00	0	2.26E+03	2.64E+03	1.15E+02	0	5.24E+03	4.63E+03	3.81E+01	3.74E+01	3.08E+01	2.08E+00	1.67E+00	0	1.02E+03	4.40E+03	1.07E+03	5.92E+01	1.97E+00	0	0	2.59E+01	1.31E+00	1.68E+03

Genome copies per Moskito

midgut																														
days	1	2	3	4	5	6	7	8	9	10	11	12	13	14	15	16	17	18	19	20	21	22	23	24	25	26	27	28	29	30
0	7.16E+05	6.34E+05	6.94E+04	3.48E+05	3.18E+05																									
3	0	4.46E+03	5.44E+02	0	0	7.20E+03	0	0	3.78E+02	0	3.44E+03	9.30E+03	1.38E+03	1.88E+04	0	0	0	3.75E+02	3.00E+03	7.67E+03	0	0	1.02E+04	1.34E+02	7.67E+02					
7	0	9.04E+01	2.67E+02	4.24E+03	0	0	4.23E+03	0	0	1.33E+03	0	1.00E+03	5.61E+02	0	4.89E+03	9.73E+04	1.31E+05	0	2.87E+05	4.67E+02	0	9.64E+04	6.30E+04	0	1.88E+05					
14	4.05E+04	3.69E+02	1.70E+05	0	4.33E+02	0	1.26E+02	0	9.69E+04	1.13E+05	4.93E+03	0	2.25E+05	1.98E+05	1.63E+03	1.60E+03	1.32E+03	8.91E+01	7.16E+01	0	4.37E+04	1.89E+05	4.59E+04	2.54E+03	8.44E+01	0	0	1.11E+03	5.61E+01	7.20E+04

Fig. 6B

Cap1

Genome copies per 3µl RNA

legs + wings																								
days	1	2	3	4	5	6	7	8	9	10	11	12	13	14	15	16	17	18	19	20	21	22	23	24
3	0	0	0	7.09E+00	6.25E+00	0	0	2.19E+01	1.24E+02	0	0	0	3.38E+00	9.75E+00	0	0	0							
7	0	2.13E+02	1.74E+02	0	1.35E+02	1.24E+02	3.69E+00	1.63E+02	5.58E+02	2.21E+03	2.77E+02	4.80E+02	4.33E+02	0	2.99E+02	1.38E+02	0	1.03E+03	1.59E+02	6.97E+01	5.38E+01	2.12E+02	0	9.13E+01
14	1.85E+03	3.66E+00	1.37E+03	1.07E+03	2.75E+03	2.04E+03	2.40E+03	6.54E+02	8.49E+02	5.49E+02	1.43E+03	1.64E+03	4.97E+02	0	1.26E+03	0	2.93E+03	4.06E+03	2.32E+03	2.65E+03	3.47E+03	2.20E+02	2.33E+03	

Genome copies per Moskito

legs + wings																								
days	1	2	3	4	5	6	7	8	9	10	11	12	13	14	15	16	17	18	19	20	21	22	23	24
3	0	0	0	3.04E+02	2.68E+02	0	0	9.39E+02	5.31E+03	0	0	0	1.45E+02	4.18E+02	0	0	0							
7	0	9.13E+03	7.46E+03	0	5.79E+03	5.31E+03	1.58E+02	6.99E+03	2.39E+04	9.47E+04	1.19E+04	2.06E+04	1.86E+04	0	1.28E+04	5.91E+03	0	4.41E+04	6.81E+03	2.99E+03	2.31E+03	9.09E+03	0	3.91E+03
14	7.93E+04	1.57E+02	5.87E+04	4.59E+04	1.18E+05	8.74E+04	1.03E+05	2.80E+04	3.64E+04	2.35E+04	6.13E+04	7.03E+04	2.13E+04	0	5.40E+04	0	1.26E+05	1.74E+05	9.94E+04	1.14E+05	1.49E+05	9.43E+03	9.99E+04	

Cap0

Genome copies per 3µl RNA

legs + wings																														
days	1	2	3	4	5	6	7	8	9	10	11	12	13	14	15	16	17	18	19	20	21	22	23	24	25	26	27	28	29	30
3	0	0	0	0	0	0	0	0	0	3.76E+01	0	0	0	0	0	0	0	0	3.64E+00	0	0	0	0	1.22E+01	0					
7	0	0	0	0	0	0	0	0	0	0	0	0	0	0	0	1.36E+01	n.d.	0	2.64E+01	0	0	9.17E-01	0	0	2.35E+03					
14	6.64E+01	0	1.95E+02	0	0	0	0	0	3.67E+01	0	0	0	1.96E+02	7.75E+01	0	0	0	2.68E+00	0	0	0	4.38E+01	1.70E+02	0	0	0	0	0	0	0

Genome copies per Moskito

legs + wings																														
days	1	2	3	4	5	6	7	8	9	10	11	12	13	14	15	16	17	18	19	20	21	22	23	24	25	26	27	28	29	30
3	0	0	0	0	0	0	0	0	0	1.61E+03	0	0	0	0	0	0	0	0	1.56E+02	0	0	0	0	5.23E+02	0					
7	0	0	0	0	0	0	0	0	0	0	0	0	0	0	0	5.83E+02	0	0	1.13E+03	0	0	3.93E+01	0	0	1.01E+05					
14	2.85E+03	0	8.36E+03	0	0	0	0	0	1.57E+03	0	0	0	8.40E+03	3.32E+03	0	0	0	1.15E+02	0	0	0	1.88E+03	7.29E+03	0	0	0	0	0	0	0

Fig. 6C

Cap1

Genome copies per 3µl RNA

Carcass																								
days	1	2	3	4	5	6	7	8	9	10	11	12	13	14	15	16	17	18	19	20	21	22	23	24
3	0	2.82E+01	6.90E+02	7.67E+00	5.50E+00	5.45E+00	2.82E+03	8.23E+02	0	3.06E+00	1.62E+00	0	8.04E+01	1.01E-01	0	2.55E+00	0							
7	0	2.78E+03	8.88E+02	0	3.00E+03	8.08E+03	6.99E+01	1.35E+03	7.97E+03	2.15E+04	7.91E+03	3.07E+03	5.05E+03	1.06E-01	3.08E+04	2.24E+03	0	5.27E+04	3.52E+03	4.82E+03	1.45E+03	1.49E+03	0	5.30E+02
14	1.73E+04	2.35E+01	7.69E+03	8.86E+03	2.45E+04	1.61E+04	2.20E+04	6.10E+03	9.36E+03	1.80E+04	2.71E+04	2.84E+04	2.16E+04	0	3.03E+04	1.14E+00	2.48E+04	2.66E+04	3.62E+04	4.08E+04	3.27E+04	1.31E+04	1.60E+04	

Genome copies per Moskito

Carcass																								
days	1	2	3	4	5	6	7	8	9	10	11	12	13	14	15	16	17	18	19	20	21	22	23	24
3	0	1.21E+03	2.96E+04	3.29E+02	2.36E+02	2.34E+02	1.21E+05	3.53E+04	0	1.31E+02	6.94E+01	0	3.45E+03	4.33E+00	0	1.09E+02	0							
7	0	1.19E+05	3.81E+04	0	1.29E+05	3.46E+05	3.00E+03	5.79E+04	3.42E+05	9.21E+05	3.39E+05	1.32E+05	2.16E+05	4.54E+00	1.32E+06	9.60E+04	0	2.26E+06	1.51E+05	2.07E+05	6.21E+04	6.39E+04	0	2.27E+04
14	7.41E+05	1.01E+03	3.30E+05	3.80E+05	1.05E+06	6.90E+05	9.43E+05	2.61E+05	4.01E+05	7.71E+05	1.16E+06	1.22E+06	9.26E+05	0	1.30E+06	4.89E+01	1.06E+06	1.14E+06	1.55E+06	1.75E+06	1.40E+06	5.61E+05	6.86E+05	

Cap0

Genome copies per 3µl RNA

Carcass																														
days	1	2	3	4	5	6	7	8	9	10	11	12	13	14	15	16	17	18	19	20	21	22	23	24	25	26	27	28	29	30
3	0	0	2.16E+01	0	0	0	0	9.33E-01	0	2.47E+03	0	1.17E+00	0	0	0	0	0	0	9.04E-01	0	3.50E+00	0	0	0	8.61E+00					
7	0	0	0	0	0	0	0	0	0	0	4.61E-02	2.42E+00	0	0	0	7.84E+01	0	0	1.12E+02	0	0	0	9.04E-01	0	6.05E+04					
14	4.27E+03	0	2.57E+03	0	0	0	0	0	8.92E+02	0	0	1.24E+00	3.54E+03	1.82E+03	0	0	0	0	0	0	0	5.35E+02	5.13E+02	0	0	0	0	0	0	0

Genome copies per Moskito

Carcass																														
days	1	2	3	4	5	6	7	8	9	10	11	12	13	14	15	16	17	18	19	20	21	22	23	24	25	26	27	28	29	30
3	0	0	9.26E+02	0	0	0	0	4.00E+01	0	1.06E+05	0	5.01E+01	0	0	0	0	0	0	3.87E+01	0	1.50E+02	0	0	0	3.69E+02					
7	0	0	0	0	0	0	0	0	0	0	1.98E+00	1.04E+02	0	0	0	3.36E+03	0	0	4.80E+03	0	0	0	3.87E+01	0	2.59E+06					
14	1.83E+05	0	1.10E+05	0	0	0	0	0	3.82E+04	0	0	5.31E+01	1.52E+05	7.80E+04	0	0	0	0	0	0	0	2.29E+04	2.20E+04	0	0	0	0	0	0	0

Fig. 7A

Cap1

Genome copies per 3µl RNA

Carcass																	
**days**	**1**	**2**	**3**	**4**	**5**	**6**	**7**	**8**	**9**	**10**	**11**	**12**	**13**	**14**	**15**	**16**	**17**
0	3.59E+01	5.95E+00	8.49E+00	7.28E+00	1.12E+01	4.38E+00	2.47E+01	2.79E+01	1.04E+01	3.87E+01	4.43E+01	2.13E+01	3.47E+01				
3	2.59E+04	1.48E+04	3.68E+04	1.27E+04	3.62E+04	3.42E+04	1.29E+04	3.75E+04	1.66E+04	1.86E+04	1.15E+04	2.45E+04	1.92E+04	1.44E+04	1.63E+04	2.00E+04	
7	9.32E+04	6.14E+04	5.79E+04	3.24E+04	9.63E+04	7.84E+04	2.09E+04	1.23E+05	4.30E+04	4.40E+04	6.36E+04	2.29E+04	4.86E+04	2.26E+04	4.56E+04	3.10E+04	2.62E+04

Genome copies per Moskito

Carcass																	
**days**	**1**	**2**	**3**	**4**	**5**	**6**	**7**	**8**	**9**	**10**	**11**	**12**	**13**	**14**	**15**	**16**	**17**
0	1.54E+03	2.55E+02	3.64E+02	3.12E+02	4.80E+02	1.88E+02	1.06E+03	1.20E+03	4.46E+02	1.66E+03	1.90E+03	9.13E+02	1.49E+03				
3	1.11E+06	6.34E+05	1.58E+06	5.44E+05	1.55E+06	1.47E+06	5.53E+05	1.61E+06	7.11E+05	7.97E+05	4.93E+05	1.05E+06	8.23E+05	6.17E+05	6.99E+05	8.57E+05	
7	3.99E+06	2.63E+06	2.48E+06	1.39E+06	4.13E+06	3.36E+06	8.96E+05	5.27E+06	1.84E+06	1.89E+06	2.73E+06	9.81E+05	2.08E+06	9.69E+05	1.95E+06	1.33E+06	1.12E+06

Cap0

Genome copies per 3µl RNA

Carcass																
**days**	**1**	**2**	**3**	**4**	**5**	**6**	**7**	**8**	**9**	**10**	**11**	**12**	**13**	**14**	**15**	**16**
0	4.15E-01	5.39E+00	2.23E+01	6.12E+00	3.15E+01	3.16E+01	1.52E+01	5.55E+01	2.31E+01							
3	6.66E+02	6.51E-01	1.21E+00	3.88E+03	1.32E+03	2.99E+02	7.26E+03	1.22E+04	1.44E+03	4.48E+03						
7	6.64E+03	1.38E+04	2.32E+04	2.42E+03	5.03E+04	1.20E+04	8.31E+03	2.40E+04	3.10E+03	5.85E+03	4.69E+00	9.54E+03	2.04E+00	2.54E+04	1.22E+04	3.19E+03

Genome copies per Moskito

Carcass																
**days**	**1**	**2**	**3**	**4**	**5**	**6**	**7**	**8**	**9**	**10**	**11**	**12**	**13**	**14**	**15**	**16**
0	1.78E+01	2.31E+02	9.56E+02	2.62E+02	1.35E+03	1.35E+03	6.51E+02	2.38E+03	9.90E+02							
3	2.85E+04	2.79E+01	5.19E+01	1.66E+05	5.66E+04	1.28E+04	3.11E+05	5.23E+05	6.17E+04	1.92E+05						
7	2.85E+05	5.91E+05	9.94E+05	1.04E+05	2.16E+06	5.14E+05	3.56E+05	1.03E+06	1.33E+05	2.51E+05	2.01E+02	4.09E+05	8.74E+01	1.09E+06	5.23E+05	1.37E+05

Fig. 7B

Cap1

Genome copies per 3µl RNA

legs + wings																	
**days**	**1**	**2**	**3**	**4**	**5**	**6**	**7**	**8**	**9**	**10**	**11**	**12**	**13**	**14**	**15**	**16**	**17**
3	1.85E+03	1.46E+03	1.03E+03	1.14E+03	2.09E+03	8.00E+02	1.32E+03	1.58E+03	1.03E+03	1.15E+03	7.53E+02	1.59E+03	1.03E+03	1.99E+03	2.85E+03	1.64E+03	
7	3.93E+03	4.09E+03	3.22E+03	1.24E+03	4.69E+03	2.73E+03	2.95E+03	3.46E+03	3.44E+03	2.72E+03	6.21E+03	4.01E+03	2.88E+03	1.51E+03	2.89E+03	1.77E+03	9.80E+02

Genome copies per Moskito

legs + wings																	
**days**	**1**	**2**	**3**	**4**	**5**	**6**	**7**	**8**	**9**	**10**	**11**	**12**	**13**	**14**	**15**	**16**	**17**
3	7.93E+04	6.26E+04	4.41E+04	4.89E+04	8.96E+04	3.43E+04	5.66E+04	6.77E+04	4.41E+04	4.93E+04	3.23E+04	6.81E+04	4.41E+04	8.53E+04	1.22E+05	7.03E+04	
7	1.68E+05	1.75E+05	1.38E+05	5.31E+04	2.01E+05	1.17E+05	1.26E+05	1.48E+05	1.47E+05	1.17E+05	2.66E+05	1.72E+05	1.23E+05	6.47E+04	1.24E+05	7.59E+04	4.20E+04

Cap0

Genome copies per 3µl RNA

legs + wings																
**days**	**1**	**2**	**3**	**4**	**5**	**6**	**7**	**8**	**9**	**10**	**11**	**12**	**13**	**14**	**15**	**16**
3	0	3.20E+01	0	9.06E-01	1.51E+02	1.83E+02	0	1.58E+01	1.86E+02	5.00E+02	1.20E+02	4.30E+01				
7	2.79E+02	8.97E+01	1.65E+02	9.53E+01	1.08E+03	1.30E+02	1.20E+02	1.81E+02	1.56E+02	3.42E+01	1.62E+00	2.06E+02	0	9.76E+01	2.01E+02	2.84E+01

Genome copies per Moskito

legs + wings																
**days**	**1**	**2**	**3**	**4**	**5**	**6**	**7**	**8**	**9**	**10**	**11**	**12**	**13**	**14**	**15**	**16**
3	0	1.37E+03	0	3.88E+01	6.47E+03	7.84E+03	0	6.77E+02	7.97E+03	2.14E+04	5.14E+03	1.84E+03				
7	1.20E+04	3.84E+03	7.07E+03	4.08E+03	4.63E+04	5.57E+03	5.14E+03	7.76E+03	6.69E+03	1.47E+03	6.94E+01	8.83E+03	0	4.18E+03	8.61E+03	1.22E+03

S1A Fig

U4.4

days	YFV-17D cap1			YFV-17D cap0		
5	320000	135000	130000	36000	27000	37000

S1B Fig

CCL-125

days	YFV-17D cap1			YFV-17D cap0		
5	650	600	750	39	39	39

39 = below detection limit

S2A Fig

BHK-J

cap1	cap0
29500000	14500000
43500000	17500000
38500000	12500000
11500000	7500000
8500000	7500000

S2B Fig

A549 wild-type

cap1	cap0
350000	600
450000	1300
200000	900

S2C Fig

A549 IFIT k.o.

cap1	cap0
170000	29500
220000	77250
70000	35250

S3A Fig

days	cap1						cap0					
0	39	39	39	39	50	39	39	39	39	39	39	39
3	2300000	1500000	1050000	2400000	1750000	2000000	39	39	39	39	39	39
5	2850000	2800000	3350000	4100000	4050000	4350000	39	50	39	200	50	200
7	1450000	1050000	750000	900000	750000	1150000	650	200	150	700	700	550
10	120000	95000	85000	140000	135000	160000	450	350	450	1100	1450	950

39 = below detection limit

S3B Fig

days	cap1			cap0		
0	39	39	39	39	39	39
3	5000	5650	22500	39	39	39
5	1750000	1750000	5550000	39	39	39
7	6500000	6900000	10000000	39	39	39
10	3000000	2400000	3250000	39	39	39

39 = below detection limit

S3C Fig

days	cap1			cap0		
0	39	39	39	50	39	39
3	18500	11000	16000	39	39	39
5	850000	600000	450000	39	39	39
7	2700000	1750000	1400000	39	39	39
10	1400000	1650000	850000	39	39	39

39 = below detection limit

S3D Fig

days	cap1			cap0		
0	39	39	39	39	39	39
3	1350000	1500000	1450000	39	39	39
5	16500000	11000000	17000000	39	39	39
7	9500000	5000000	8000000	39	50	50
10	2600000	1800000	1850000	50	100	50

39 = below detection limit

S4A Fig

Asibi	Asibi/17D SP	17D/Asibi NS4A**-3'UTR	Asibi/17D NS5	17D/Asibi NS4A-NS5	17D/Asibi NS1-5
4200000	600000	485000	3300000	490000	2400000
3550000	700000	600000	2000000	580000	1750000
3750000	900000	350000	3650000	560000	2000000

17D	17D/Asibi SP	17D/Asibi NS1-4A*	17D/Asibi NS5	17D/Asibi 2K-NS5	17D/Asibi 2K-NS4B	17D/Asibi NS4A**	17D/Asibi NS1-4B
10500	235000	9500	41000	200000	1350000	18500	5000
8500	300000	11000	34000	145000	1500000	11000	5650
8500	260000	210000	44500	105000	1450000	16000	22500

S4B Fig

hours	YFV-Asibi			YFV-17D		
0	39	39	39	39	39	39
12	39	39	500	39	500	39
24	39	39	39	39	39	39
48	360000	295000	355000	6750	7050	13500
72	3400000	3800000	3850000	260000	175000	210000

39 = below detection limit

S5 Fig

	dsGFP			dsRenilla		
Aag2 AF5	927273	1341771	786332	62	48	55
Aag2 AF319	1888303	2151560	3078532	164247	182413	194105

S6 Fig

Aag2

hours	cap1								
4	3733	4014	4179	3578	3099	3302	4895	4000	3119
8	3756	4344	4488	3221	3026	3291	4387	4044	4369
22	3617	4064	3738	2445	2754	2952	4732	4021	3890

hours	cap0								
4	2789	2622	2446	1690	1585	1508	1939	2114	2426
8	2903	2857	2461	1600	1473	1568	1880	2112	1704
22	2711	3100	2542	1409	1299	1523	1693	1889	1630

p Value
<0,0001
<0,0001
0.0008

S7A Fig

cap1

PFU/ carcass

days																	
0	157	1065	1155	330	1155	1080											
3	0.75	420	180	45	105	15	15										
5	60	60	30	5	15	375	2850										
7	60	0.75	255	45	795	30	45	7500	75								
10	315	135	6300	10	945	0.75	285	240	390	0.75	435	540					
14	525	105	30	1545	0.75	15	225	300	360	150	90	75	150	195	390	135	60
21	180	30		1065	1035	195	11250	30	240	*0.75*	1215	150	2100	990	465	135	

cap0

PFU/carcass

days																
0	562	5400	495	780	435	3450										
3	0.75	0.75	0.75	0.75	0.75	0.75	0.75									
5	0.75	0.75	0.75	0.75	0.75	0.75	0.75									
7	0.75	0.75	0.75	0.75	0.75	0.75	0.75	0.75	0.75							
10	0.75	0.75	0.75	0.75	0.75	0.75	0.75	0.75	0.75	0.75	0.75	0.75	0.75	0.75	0.75	0.75
14	0.75	0.75	0.75	0.75	0.75	0.75	0.75	0.75	0.75	0.75	0.75	0.75	0.75	0.75		
21	0.75	0.75	0.75	0.75	0.75	0.75	0.75	0.75	0.75	0.75	0.75	0.75	0.75	0.75	0.75	0.75

0.75 = below detection limit

S7B Fig

cap1

PFU/ legs+wings

days																										
0	0.75	0.75	0.75	0.75	0.75	0.75																				
3	0.75	0.75	0.75	0.75	0.75	0.75	0.75																			
5	0.75	0.75	0.75	0.75	0.75	0.75	0.75																			
7	0.75	0.75	0.75	0.75	0.75	0.75	0.75	2.5																		
10	5	10	37.5	0.75	0.75	0.75	0.75	38	86	2	28	38														
14	0.75	0.75	0.75	0.75	5	*0.75*	0.75	7.5	0.75	7.5	46	0.75	0.75	0.75	6	2	4	10	18	6	2					
21	0.75	0.75	7.5	0.75	0.75	0.75	0.75	5	52	24	0.75	14	4	2	0.75	4	8	2	2	8	10	8	0.75	14	4	8

cap0

PFU/legs+wings

days																				
0	0.75	0.75	0.75	0.75	0.75	0.75														
3	0.75	0.75	0.75	0.75	0.75	0.75	0.75													
5	0.75	0.75	0.75	0.75	0.75	0.75	0.75													
7	0.75	0.75	0.75	0.75	0.75	0.75	0.75	0.75	0.75											
10	0.75	0.75	0.75	0.75	0.75	0.75	0.75	0.75	0.75	0.75	0.75	0.75	0.75	0.75	0.75	0.75				
14	0.75	0.75	0.75	0.75	0.75	0.75	0.75	0.75	0.75	0.75	0.75	0.75	0.75	0.75	0.75	0.75	0.75	0.75	0.75	0.75
21	0.75	0.75	0.75	0.75	0.75	0.75	0.75	0.75	0.75	0.75	0.75	0.75	0.75	0.75	0.75	0.75	0.75	0.75		

0.75 = below detection limit

S8 Fig

C6/36 (moi 0.1)

days	YFV-17D cap1		YFV-17D cap1	
0	39	39	39	39
3	195000	350000	39	39
5	1600000	2150000	39	39
7	1350000	1100000	39	39

39 = below detection limit
